# Tandem CTCF sites function as insulators to balance spatial chromatin contacts and topological enhancer-promoter selection

**DOI:** 10.1186/s13059-020-01984-7

**Published:** 2020-03-23

**Authors:** Zhilian Jia, Jingwei Li, Xiao Ge, Yonghu Wu, Ya Guo, Qiang Wu

**Affiliations:** 1 MOE Key Lab of Systems Biomedicine, Center for Comparative Biomedicine, State Key Lab of Oncogenes and Related Genes, Shanghai Cancer Institute, Joint International Research Laboratory of Metabolic & Developmental Sciences, Institute of Systems Biomedicine, Xin Hua Hospital, Shanghai Jiao Tong University, Shanghai, 200240 China; 2 The Third Affiliated Hospital of Guangzhou Medical University, Guangzhou, 510150 China

**Keywords:** CTCF, Insulator, Promoter/enhancer selection, 3D genome, Gene regulation, Loop extrusion, Cohesin, Chromatin polymer simulation, Bayesian networks, Topological spatial contacts

## Abstract

**Background:**

CTCF is a key insulator-binding protein, and mammalian genomes contain numerous CTCF sites, many of which are organized in tandem.

**Results:**

Using CRISPR DNA-fragment editing, in conjunction with chromosome conformation capture, we find that CTCF sites, if located between enhancers and promoters in the protocadherin (*Pcdh*) and *β-globin* clusters, function as an enhancer-blocking insulator by forming distinct directional chromatin loops, regardless whether enhancers contain CTCF sites or not. Moreover, computational simulation in silico and genetic deletions in vivo as well as dCas9 blocking in vitro revealed balanced promoter usage in cell populations and stochastic monoallelic expression in single cells by large arrays of tandem CTCF sites in the *Pcdh* and immunoglobulin heavy chain (*Igh*) clusters. Furthermore, CTCF insulators promote, counter-intuitively, long-range chromatin interactions with distal directional CTCF sites, consistent with the cohesin “loop extrusion” model. Finally, gene expression levels are negatively correlated with CTCF insulators located between enhancers and promoters on a genome-wide scale. Thus, single CTCF insulators ensure proper enhancer insulation and promoter activation while tandem CTCF topological insulators determine balanced spatial contacts and promoter choice.

**Conclusions:**

These findings have interesting implications on the role of topological chromatin insulators in 3D genome folding and developmental gene regulation.

## Background

Genetic studies have long described the phenomenon of position effect variegation (PEV) [1], suggesting that the spatial organization of chromatin domains has an important influence on gene expression [2–4]. Early studies revealed that boundary elements, also known as insulators, restrict promoter activity from the position effects of its chromatin contexts [5, 6]. In particular, through a series of transgenic experiments, Grosveld and colleagues have identified dominant boundary elements flanking the human *β-globin* locus, which determine its position-independent expression in transgenic mice [5]. It has since been established that insulators play an essential role in shielding the position effects of chromatin conformation and in blocking enhancers or silencers from improperly activating or repressing non-cognate promoters, respectively [2, 3, 6–8].

The mammalian CTCF is the best characterized genome architectural protein that binds to insulator elements [2, 8]. CTCF directionally and dynamically binds to tens of thousands of CTCF-binding sites (CBS elements) in mammalian genomes through the combinatorial usage of its 11 zinc fingers [9, 10]. CTCF, together with the associated cohesin, a ring-shaped complex embracing DNA, mediates genome-wide long-range chromatin interactions [2]. Interestingly, these interactions are preferentially formed between forward-reverse convergent CBS pairs [11–14]. The CBS elements in the boundaries between neighboring chromatin domains are configured in a reverse-forward divergent orientation, which are thought to restrict enhancer activity to promoters within each insulated neighborhood [12, 15, 16]. Thus, the boundary CBS elements may function as insulators to block cohesin loop extrusion [11, 12, 17–20]. However, whether and how internal CBS elements function as insulators remain incompletely understood.

Recent topologically associated domain (TAD) perturbations by targeted degradation of CTCF or cohesin revealed that loss of chromatin loops genome-wide differentially affects gene expression [21, 22]. Numerous studies have shown that CTCF/cohesin-mediated chromatin loop domains or TADs are important for gene regulation in specific loci [12, 15, 16, 23]. Insertion, mutation, deletion, inversion, or duplication of CBS elements alters chromatin topology and gene expression [12, 14–16, 18, 23–25]. Emerging evidence suggests that spatial control of genome topology by CTCF/cohesin regulates gene expression; however, how numerous CBS elements in mammalian genomes function as insulators to control proper promoter activation and its balanced usage remains obscure.

**Fig. 1:**
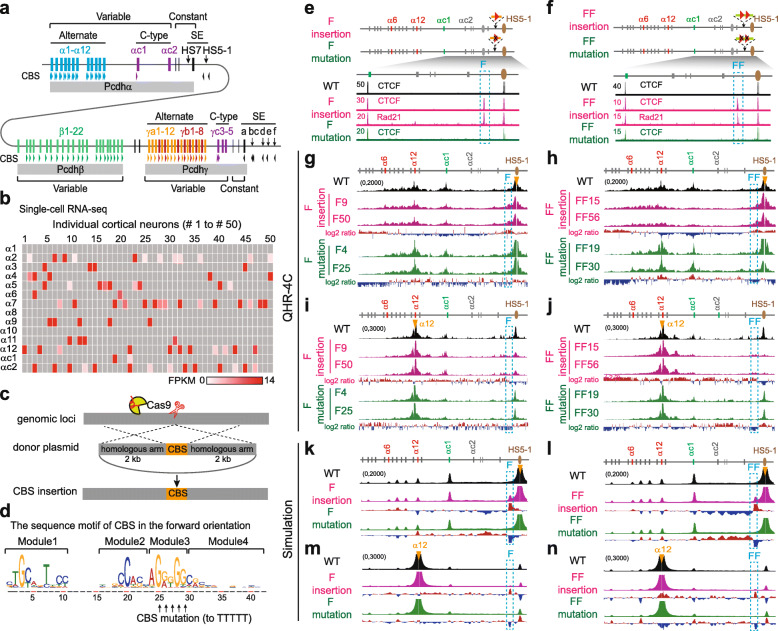
CTCF sites as insulators in *Pcdhα* through directional chromatin looping. **a** Schematic of the three mouse *Pcdh* clusters. Genomic organizations of the *Pcdh α* and *γ* clusters are similar. Both contain variable exons, each of which is preceded by its own promoter and spliced to respective downstream constant exons. The *Pcdhβ* cluster contains only variable exons. Each of the variable exons (except *αc2*, *β1*, *γc4*, and *γc5*) carries one or two forward-oriented CTCF sites (indicated by tandem arrowheads). Two distal enhancers, *HS7* (with no CBS) and *HS5-1* (flanked by two reverse-oriented CBS elements of *HS5-1a* and *HS5-1b*), are located between the *Pcdh α* and *β* clusters. The super-enhancer of the *Pcdh β* and *γ* clusters, which contains a tandem array of reverse-oriented CBS elements, is located downstream of *Pcdhγ*. CBS, CTCF binding site; HS, hypersensitive site; SE, super-enhancer. **b** Single-cell expression patterns of the *Pcdhα* genes in mouse cortical cells. **c** CRISPR insertion of CBS elements by homologous recombination. **d** Schematic of the sequence motif (CTCF binding site contains four modules in the order of modules 1–4 for the forward orientation) and its mutation. **e**, **f** CTCF and Rad21 ChIP-seq of single-cell CRISPR clones with one or two forward-oriented CBS elements inserted into the location between the *Pcdhα* cluster and its downstream *HS5-1* enhancer. **g**–**j** QHR-4C interaction profiles of four CRISPR clones (F9 and F50 for one-CBS insertion; FF15 and FF56 for two-CBS insertion) as well as their CBS mutations (F4 and F25; FF19 and FF30) with *HS5-1* (**g**, **h**) or *α12* (**i**, **j**) as a viewpoint (VP), represented by arrowheads. **k**–**n** Chromatin interaction profiles from an ensemble of 350,000 simulated conformations with *HS5-1* (**k**, **l**) or *α12* (**m**, **n**) as a viewpoint, corresponding to **g**–**j**, respectively. Log2 ratios (insertion vs wild-type or mutation vs insertion) are shown under the 4C profiles

Similar to the enormous diversity of DSCAM1 proteins in *Drosophila*, combinatorial *cis*- and *trans*-interactions between clustered cell surface protocadherin (Pcdh) proteins in mammals, encoded by the three closely linked *α*, *β*, and *γ* gene clusters (Fig. 1a in mice), endow individual neurons with a unique identity code and specific self-recognition module, which are required for neuronal migration and connectivity, dendrite self-avoidance and tiling, and axon outgrowth and even spacing in the brain [26–32]. The *Pcdh α* and *γ* clusters contain more than a dozen highly similar, tandem-arrayed, unusually large “alternate” variable exons and 2 or 3 divergent C-type variable exons, respectively (Fig. 1a). These variable exons are followed by 3 downstream small constant exons, reminiscent of the variable and constant genome organizations of immunoglobulin (*Ig*), T cell receptor (*Tcr*), and UDP-glucuronosyltransferase (*Ugt*) clusters [26, 28, 33]. Each of the *Pcdhα* “alternate” variable exons (*α1*-*α12* in mice) carries its own promoter, which is flanked by two forward-oriented CBS elements (Fig. 1a). However, the *αc1* promoter carries only one forward-oriented CBS, and the *αc2* promoter has no CBS element (Fig. 1a). Two distal *Pcdhα* enhancers, *HS7* and *HS5-1*, are located downstream, and one of which, *HS5-1*, is flanked by two reverse-oriented CBS (*HS5-1a* and *HS5-1b*) elements [34, 35]. Multiple long-distance chromatin interactions between these remote enhancers and *Pcdhα* target promoters form a transcription hub and determine the promoter choice, but the underlying mechanisms are unknown [35, 36].

Here, by a combination of CRISPR DNA-fragment editing [37, 38] and chromosome conformation capture [3] experiments as well as Bayesian modeling, we show that ectopic and endogenous CTCF sites function as topological insulators in an orientation-independent manner through CTCF-mediated directional chromatin looping throughout the mammalian genome. In addition, genetic experiments, in conjunction with computational polymer simulation of cohesin loop extrusion, demonstrate that tandem-arrayed CTCF sites ensure stochastic spatial accessibility of repertoires of promoters and their balanced usage.

## Results

### Exogenous directional CTCF sites function as protocadherin insulators in cellular model in vitro

To investigate the mechanisms of cell-specific *Pcdh* gene expression in the brain, we performed single-cell RNA-seq of mouse cortical neurons and found members of the *Pcdhα* cluster are expressed in single neurons in a combinatorial and stochastic manner (Fig. 1b), similar to the stochastic monoallelic expression patterns of *Pcdhα* in single Purkinje cells in the cerebellum [28, 39]. In addition, maximum likelihood modeling confirms the stochastic monoallelic expression patterns in single cells of the mouse neocortex (Additional file 1: Figure S1a,b) [40].

We next made use of the HEC-1-B cell line, which monoallelically expresses *α6* and *α12* (Additional file 1: Figure S1c-f; note that humans have 13 alternate variable exons), as a single-cell model system to investigate mechanisms of gene regulation [12]. We performed CBS insertions by DNA-fragment editing and screened for single-cell CRISPR clones (Fig. 1c, d) [37, 38]. We first inserted single (“F”) or tandem (“FF”) forward-oriented CBS elements into the location between the *Pcdhα* cluster and its *HS5-1* enhancer (Fig. 1d–f) and carried out quantitative high-resolution chromosome conformation capture copy followed by next-generation sequencing (QHR-4C) experiments (Additional file 1: Figure S2). QHR-4C revealed prominent long-distance chromatin interactions between *HS5-1* and the inserted CBS elements, and a concurrent decrease of chromatin interactions between *HS5-1* and the *Pcdhα* promoters (Fig. 1g–j and Additional file 1: Figure S3a,b). In addition, CBS mutations abolish these effects (Fig. 1g–j and Additional file 1: Figure S3a,b). Consistent with the decrease of enhancer-promoter interactions, RNA-seq revealed a significant decrease of *α6* and *α12* expression levels, and CBS mutations rescue their expression (Additional file 1: Figure S3c,d). In summary, the inserted forward-oriented CBS elements block the long-distance chromatin spatial contacts between the *HS5-1* enhancer and its target promoters and thus function as chromatin insulators by competing with the target *Pcdhα* promoters.

We next inserted three different reverse-oriented CBS elements each into distinct locations in the *Pcdhα* cluster (Additional file 1: Figures S3e-j and S4). We found that each competes with the *HS5-1* enhancer to form long-distance chromatin interactions with target promoters and thus functions as an insulator (Additional file 1: Figures S3e-j and S4). Finally, we inserted reverse-forward CBS pairs (“RF” or “RRFF”) into the location between the *Pcdhα* cluster and the *HS5-1* enhancer. We found that they also function as insulators (Additional file 1: Figures S5 and S6).

### Forward-reverse CTCF sites do not compromise insulation activity

Previous studies demonstrated that *Drosophila* paired insulators compromise the insulation activity of each other [41, 42]. To test the orientation of mammalian insulators, we inserted four tandem CBS elements in a forward-reverse configuration between the *Pcdhα* cluster and its *HS5-1* enhancer (Additional file 1: Figure S7a). We found, surprisingly, these inward forward-reverse CBS elements still function as insulators. Specifically, QHR-4C and RNA-seq revealed a significant decrease of chromatin interactions between *HS5-1* and the *Pcdhα* promoters as well as their decreased expression (Additional file 1: Figure S7b-f). This suggests that, different from fly insulators, the mammalian forward-reverse tandem CTCF sites do not compromise their insulation activities. As a control, the inserted outward reverse-forward boundary CBS elements function as insulators as expected (Additional file 1: Figures S5 and S6).

We conclude that both forward and reverse ectopic CBS elements function as insulators for the *Pcdhα* genes through CTCF-mediated directional looping (Fig. 1 and Additional file 1: Figures S3-S7), namely, CTCF insulators function in an orientation-independent manner. However, their insulation mechanisms are distinct. The forward or reverse CBS elements form long-distance chromatin interactions with the *Pcdhα* enhancers or promoters (presumably by cohesin sliding through the oncoming convergent CTCF sites, Additional file 1: Figure S7b,c), respectively, in an orientation-dependent manner. Thus, the relative locations and orientations of inserted CBS elements determine their insulation specificity through directional looping to distinct CTCF sites in the *Pcdhα* cluster.

### CTCF insulators enhance distal promoter usage

Interestingly, the inserted CTCF insulators mainly block enhancer contacts with the proximal *Pcdhα* promoters (Fig. 1g, h and Additional file 1: Figures S3f, S5b, S6b, and S7b). Surprisingly, the insertion of CTCF insulators augments long-distance chromatin interactions between the *HS5-1* enhancer and the distal *Pcdhα* promoters (Fig. 1g, h and Additional file 1: Figures S3f, S5b, S6b, and S7b). To understand this puzzling phenomenon, we simulated polymer conformation dynamics of the *Pcdhα* cluster by “two-headed” cohesin loop extrusion on a coarse-grained chromatin fiber (Additional file 1: Figure S7g), based on the locations and relative orientations of the CBS elements that are dynamically bound by CTCF proteins (Additional file 1: Figure S8a-c) [9, 10, 12, 18–20].

We assume that cohesin slides along the *Pcdhα* chromatin fiber until it encounters an opposite CBS element or another sliding cohesin (Additional file 1: Figure S7g) [18, 19, 43]. Remarkably, computational 3D polymer simulations revealed that, in addition to proximal *Pcdhα* promoter insulation, continuous cohesin extrusion of chromatin loops results in a significant increase of chromatin interactions between the *HS5-1* enhancer and the distal *Pcdhα* promoters upon insertions of various CTCF insulators (Fig. 1k, l and Additional file 1: Figures S3i, S5f, S6f, and S7e), consistent with the observed data from the QHR-4C experiments (Fig. 1g, h and Additional file 1: Figures S3f, S5b, S6b, and S7b). Finally, by applying the relative maximum entropy approach with independent Gaussian errors, we optimized our polymer simulations and obtained strong evidence that CTCF insulators promote distal chromatin interactions (Additional file 1: Figure S8d).

We next simulated chromosome conformation of the *Igh* cluster which also contains a large repertoire of tandem variable CTCF sites (Additional file 1: Figure S8e,f) [33] and found that, similar to that in the *Pcdhα* cluster, insertion of various CTCF insulators in different orientations also augments distal variable gene segment (*V*_*H*_) utilization (Additional file 1: Figure S8f,g). Thus, CTCF-mediated directional looping of tandem-arrayed CBS elements determines the promoter balance of both *Pcdhα* and *Igh* gene clusters.

### Topological looping of distal-to-distal CTCF sites in the *Pcdh β/γ* clusters

**Fig. 2:**
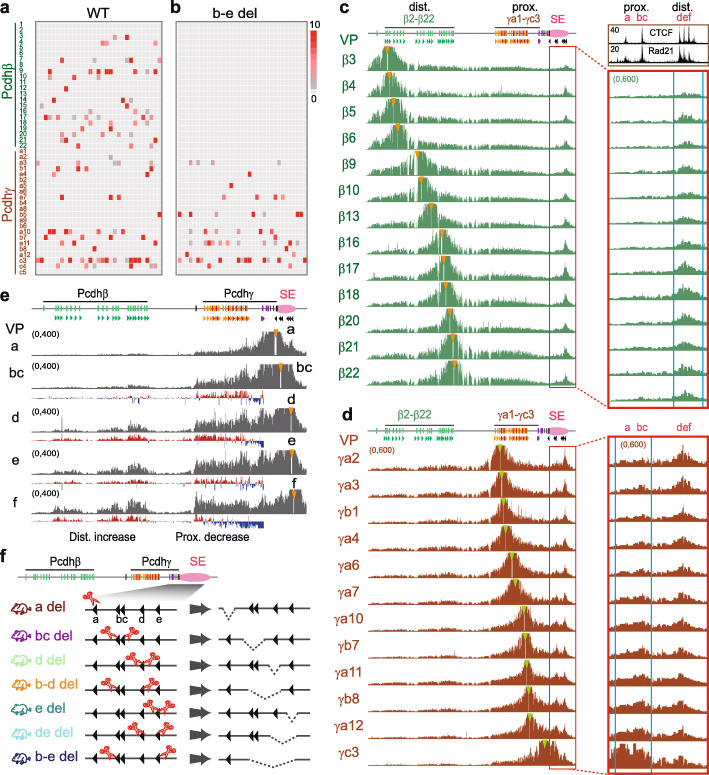
Tandem CTCF sites balance the usage of *Pcdh β* and *γ* promoters. **a**, **b** Single-cell RNA-seq of cortical neurons of the WT (**a**) and *CBS b-e* (**b**) deletion mice. Note that the absence of *Pcdhβ* expression in single cortical neurons of the CTCF sites *b-e* deletion mice. **c** QHR-4C profiles with a repertoire of the *Pcdhβ* promoters as a viewpoint show that they form spatial chromatin contacts with the distal CTCF sites *d-f*, but not proximal CTCF sites *a-c*, within the super-enhancer in the mouse cortical tissues. Inset in the upper right corner, ChIP-seq with a specific antibody against CTCF or Rad21. **d** QHR-4C profiles with a repertoire of the *Pcdhγ* promoters as a viewpoint show that, in addition to distal CTCF sites, they form gradually increased spatial chromatin contacts with the proximal CTCF sites *a-c* in the super-enhancer in the mouse cortical tissues. **e** QHR-4C interaction profiles with a repertoire of increasingly distal CTCF sites in the super-enhancer as a viewpoint show increased spatial chromatin contacts with the *Pcdhβ* cluster. **f** Schematic of the deletions of individual CTCF sites or their combinations in the *Pcdh β* and *γ* super-enhancer in mice. SE, super-enhancer; del, deletion

Similar to the *Pcdhα* cluster, the promoter of each member of the *Pcdh β* and *γ* clusters (except *β1*, *γc4*, and *γc5*) carries a forward CBS, and the downstream super-enhancer contains a tandem array of reverse-oriented CBS elements (Fig. 1a) [12, 44]. It is not clear how members of the *Pcdh β* and *γ* clusters are regulated by these tandem reverse CBS elements. Single-cell RNA-seq and maximum likelihood modeling demonstrated that single cortical neurons express random combinations of roughly up to 4 isoforms of the *Pcdhβ* family and 4 isoforms of the *Pcdhγ* family in the mouse brain (Fig. 2a and Additional file 1: Figure S9a). However, the deletion of CTCF sites *b-e* in the super-enhancer mainly impairs the expression of members of the *Pcdhβ* cluster in single cells in the mouse cortex (Fig. 2b compared with Fig. 2a).

To investigate whether tandem CTCF sites in the *Pcdh β* and *γ* clusters and their downstream super-enhancer also balance spatial chromatin contacts and promoter choice, we performed QHR-4C experiments with a repertoire of the *Pcdh β* and *γ* promoters as a viewpoint using mouse cortical tissues (Fig. 2c, d). Remarkably, the regulation of the *Pcdh β* and *γ* promoters appears topological. Namely, there are specific long-distance chromatin interactions between members of the *Pcdhβ* cluster and the distal CTCF sites *d-f*, but not proximal CTCF sites *a-c* (despite that all six CTCF sites *a-f* are bound by CTCF and cohesin, inset in the upper right corner of Fig. 2c), in the downstream super-enhancer (Fig. 2c). However, when using a repertoire of the *Pcdhγ* promoters as a viewpoint, in addition to the distal CTCF sites *d-f*, there appear increased spatial chromatin contacts with the proximal CTCF sites *a-c* of the downstream super-enhancer (Fig. 2d). Finally, to confirm this spatial regulation of the *Pcdh β* and *γ* promoters, we performed QHR-4C experiments with each of the super-enhancer CBS repertoire as a viewpoint and found increased long-range chromatin interactions between distal forward CTCF sites of the *Pcdh* variable promoters and distal reverse CTCF sites of the super-enhancer (Fig. 2e). Therefore, members of the *Pcdh β* and *γ* clusters are regulated topologically by the distal and proximal CTCF sites, respectively, within the downstream super-enhancer.

### Tandem CTCF sites balance usage of *Pcdh β* and *γ* promoters

**Fig. 3:**
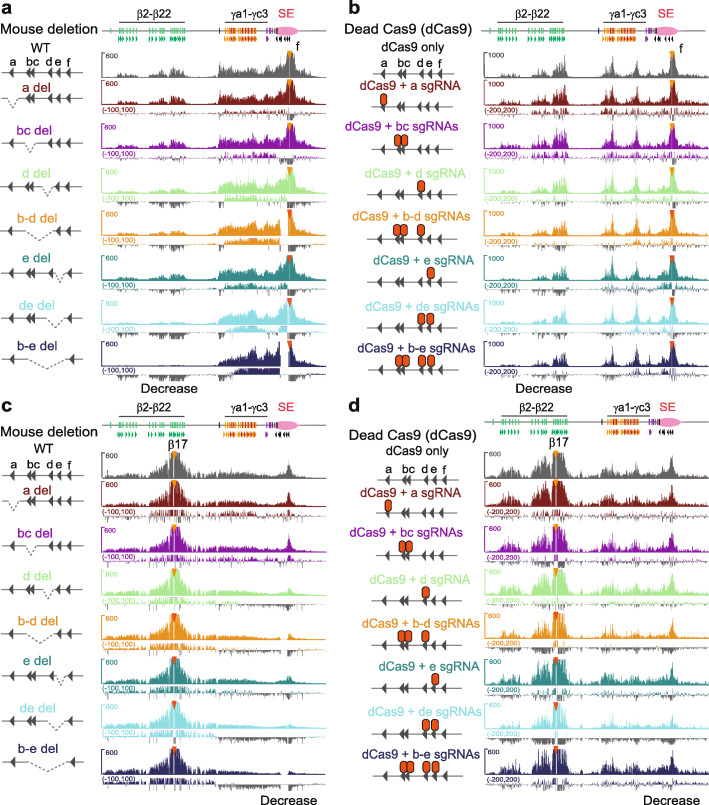
The topology of spatial chromatin contacts of super-enhancer with the *Pcdh β* and *γ* promoters. **a** QHR-4C interaction profiles with the CBS *f* of the downstream super-enhancer as a viewpoint in cortical tissues of mice with a series of deletions of individual CTCF sites or their combinations. **b** QHR-4C interaction profiles with the CBS *f* of the downstream super-enhancer as a viewpoint using cells transfected with dCas9 only or dCas9 with sgRNAs targeting individual CTCF sites or their combinations to pinpoint the effects to CTCF sites. **c**, **d** QHR-4C interaction profiles with the upstream *Pcdhβ17* promoter CBS as a viewpoint in mice with CTCF site deletions or in cells with dCas9-blocked CTCF sites confirm the decreased interactions with the downstream super-enhancer. WT, wild-type; del, deletion

To further investigate the mechanism of tandem-arrayed CBS function in the super-enhancer, we generated a series of deletions of individual CTCF sites or their combinations in mice (Fig. 2f and Additional file 1: Figure S9b). QHR-4C experiments revealed that deletions of these CTCF sites result in a significant increase of long-distance chromatin interactions between the *Pcdhγ* promoters and the super-enhancer, as well as a significant decrease of long-distance chromatin interactions between the *Pcdhβ* promoters and the super-enhancer (Fig. 3a and Additional file 1: Figures S10 and S11).

To pinpoint these topological effects to CTCF sites but not enhancers, we used catalytically inactive Cas9 (dCas9 for dead Cas9) CRISPR systems to specifically block each CBS within deletions without perturbing enhancers. QHR-4C experiments confirmed a significant increase with proximal *Pcdhγ* and a significant decrease with *Pcdhβ* (Fig. 3b). Finally, we confirmed this topological regulation in deletion mice and dCas9-blocking system by QHR-4C with the *Pcdhβ17* promoter as a viewpoint (Fig. 3c, d). We conclude that, similar to the *Pcdhα* and *Igh* clusters, endogenous tandem CTCF sites function as topological insulators to balance spatial enhancer contacts and promoter choice of the *Pcdh β* and *γ* clusters.

### Endogenous CTCF sites function as protocadherin insulators

**Fig. 4:**
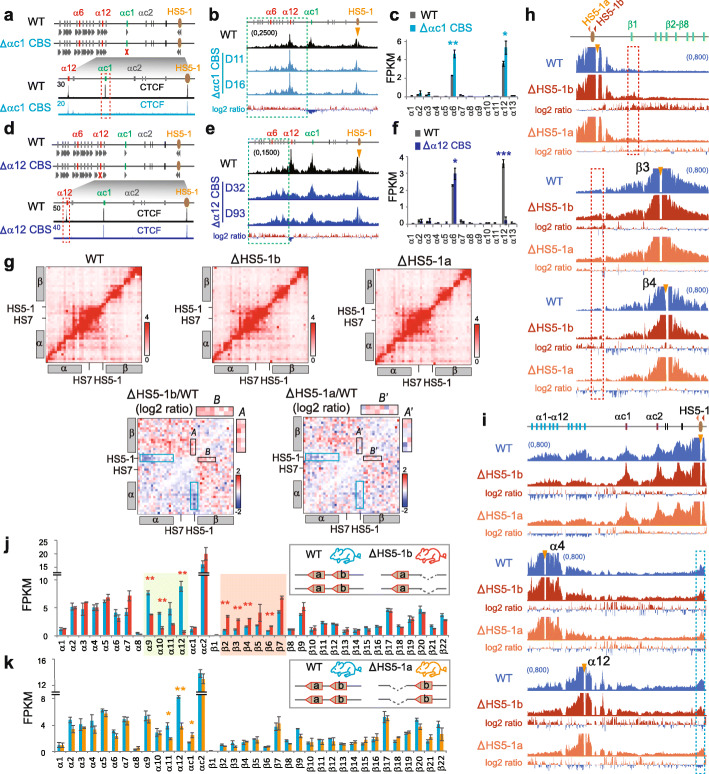
Endogenous CTCF sites as *Pcdh* insulators. **a** CTCF ChIP-seq of the *Pcdhαc1* CBS deletion CRISPR HEC-1-B cell clones. **b** QHR-4C profiles of the long-range chromatin contacts with *HS5-1* as a viewpoint in two single-cell CRISPR clones (D11, D16) with the deletion of the endogenous *αc1* CBS. Log2 ratios (deletion vs wild-type) are also shown. **c** RNA-seq of the WT and *αc1* CBS-deleted CRISPR clones. **d**–**f** Corresponding to **a**–**c**, respectively, but with *α12* CBS deletion in two single-cell CRISPR clones (D32, D93). **g** 5C interaction profiles of the *Pcdh α* and *β* clusters in cortical tissues of the *HS5-1b* or *HS5-1a* CBS deletion mice. The log2 ratios of chromatin interactions of *HS5-1* with *Pcdhα* or 5′ isoforms of *β* gene repertoire are highlighted by blue or black rectangles, respectively. Note the significant increase of chromatin interactions between *HS5-1* with 5′ isoforms of the *Pcdhβ* cluster upon the *HS5-1b* deletion as indicated by enlargement of Insets *A* and *B*, compared with no alteration upon the *HS5-1a* deletion as indicated by enlargement of Insets *A′* and *B′.***h**, **i** QHR-4C confirmed the increased interactions with 5′ isoforms of the *Pcdhβ* cluster and the decreased interactions with the *Pcdhα* cluster. **j**, **k** RNA-seq revealed increased expression levels of the 5′ isoforms of the *Pcdhβ* cluster in the homozygous CBS *HS5-1b* deletion (**j**) mice in comparison with the *HS5-1a* deletion (**k**) mice as controls. Data as mean ± SD, *p < 0.05, **p < 0.01, ***p < 0.001. One-tailed Student’s *t* test

We next tested whether each of the endogenous tandem arrays of the forward-oriented *Pcdh* CBS elements functions as an insulator. We found that the deletion of the *αc1* CBS element results in a significant increase of long-distance chromatin interactions between *HS5-1* and the *Pcdhα* genes upstream of *αc1* (Fig. 4a, b). In addition, this deletion results in a significant increase of *α6* and *α12* expression levels (Fig. 4c). Moreover, the deletion of the *α12* CBS element also results in a significant increase of chromatin interactions between *HS5-1* and the upstream *Pcdhα* genes (Fig. 4d, e) as well as of the *α*6 expression levels (Fig. 4f). Together, these data suggest that each endogenous CBS element functions as an insulator for its respective upstream *Pcdhα* genes.

To investigate whether each of the two reverse-oriented CBS elements (*HS5-1a* and *HS5-1b*) flanking the *HS5-1* enhancer also functions as an insulator, we deleted each of them in mice in vivo and performed 5C, QHR-4C, and RNA-seq experiments using mouse cortical tissues (Fig. 4g–k). Deletion of the *HS5-1b* CBS (Additional file 1: Figure S12a,b), which is at the boundary between the *Pcdhα* and *Pcdhβγ* subTADs [12], results in an aberrant increase of long-distance chromatin interactions between *HS5-1* and the 5′ isoforms of the *Pcdhβ* cluster (Fig. 4g, h) as well as an aberrant activation of their promoters (Fig. 4j). Remarkably, even for the *Pcdhβ1* promoter, which does not carry CBS, the long-distance chromatin interactions with *HS5-1* are still aberrantly increased, suggesting that *HS5-1b* CBS functions as an insulator to block the *HS5-1* enhancer from the improper activation of the *Pcdhβ1* promoter (Fig. 4h). By contrast, both the chromatin interactions of *HS5-1* with the proximal alternate *Pcdhα* genes as well as their expression levels are significantly decreased (Fig. 4g, i, j). This suggests that the boundary *HS5-1b* CBS element is an insulator that restricts the *HS5-1* enhancer activity from the aberrant activation of the *Pcdhβ* promoters. As a control, homozygous deletion of the internal *HS5-1a* CBS element (Additional file 1: Figure S12a,b) results in no expression alteration of the 5′ isoforms of the *Pcdhβ* cluster (Fig. 4k). Therefore, although both *HS5-1a* and *HS5-1b* CBS elements are required for bridging the *HS5-1* enhancer to the *Pcdhα* promoters (Fig. 4g, i–k), only the boundary *HS5-1b* CBS element functions as an insulator blocking the *HS5-1* enhancer activity from aberrantly activating the *Pcdhβ* genes.

Finally, to further investigate whether the insulation activity of the CBS *HS5-1b* is orientation-dependent, we generated a mouse line with the CBS *HS5-1b* inverted (Additional file 1: Figure S12a,b). Strikingly, neither the expression levels of 5′ isoforms of the *Pcdhβ* cluster nor their long-distance chromatin interactions with the *HS5-1* enhancer are significantly increased (Additional file 1: Figure S12c-e). By contrast, both expression levels of the proximal alternate *Pcdhα* genes and their long-distance chromatin interactions with the *HS5-1* enhancer are significantly decreased (Additional file 1: Figure S12c,f). Thus, the inverted CBS *HS5-1b* still functions as an insulator to block the *HS5-1* enhancer from improperly activating the *Pcdhβ* cluster but no longer is able to bridge the *HS5-1* enhancer with the proximal alternate *Pcdhα* genes. This again demonstrates that the insulation activity of CTCF insulators is orientation-independent, but the directional looping of CTCF sites is orientation-dependent. We conclude that both endogenous CTCF sites in the native genomic locations and inserted exogenous CTCF sites in ectopic locations function as insulators in an orientation-independent manner.

### Insulators for *Pcdh* and *β-globin* enhancers with no CTCF site

**Fig. 5:**
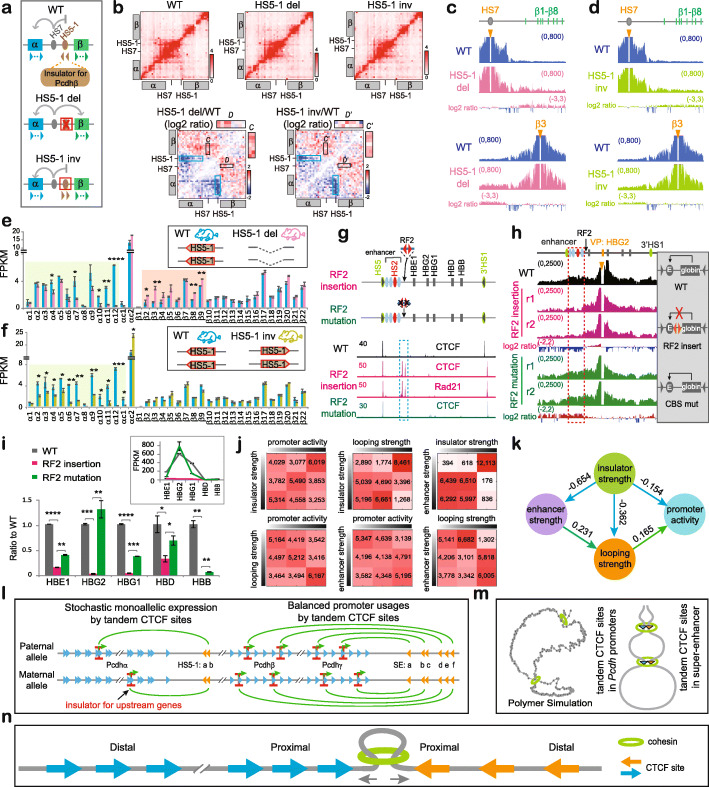
Insulators for enhancers with no CTCF site. **a** Schematic of the *HS5-1* CBS elements as an insulator of the *HS7* enhancer for the *Pcdhβ* genes. **b** 5C interaction profiles of the *Pcdh α* and *β* clusters in the *HS5-1* deletion (del) or inversion (inv) mice in vivo. The log2 ratios of chromatin interactions of *HS5-1* with *Pcdhα* and of *HS7* with 5′ isoforms of the *Pcdhβ* cluster are highlighted by blue or black rectangles, respectively. Note the significant increase of chromatin interactions between *HS7* with 5′ isoforms of the *Pcdhβ* cluster upon *HS5-1* deletion as indicated by the enlargement of insets *C* and *D*. **c** QHR-4C with *HS7* or *β3* as a viewpoint confirms the increased interactions between *HS7* and 5′ isoforms of the *Pcdhβ* cluster in homozygous *HS5-1* deletion mice. **d** QHR-4C with *HS7* or *β3* as a viewpoint confirms no significant alteration of interactions between *HS7* and 5′ isoforms of the *Pcdhβ* cluster in homozygous *HS5-1* inversion mice. **e** RNA-seq of cortical tissues of the WT and *HS5-1* deletion mice. **f** RNA-seq of cortical tissues of the WT and *HS5-1* inversion mice. **g** CTCF and Rad21 ChIP-seq of human single-cell *β-globin* CRISPR clones with insertion of a pair of reverse-forward CBS elements (“RF2”). **h** QHR-4C profiles with the human *β-globin HBG2* promoter as a viewpoint. **i** RNA-seq reveal decreased expression levels (normalized to WT) of the human *β-globin* repertoire. The actual expression levels are shown in the inset. Data as mean ± SD, *p < 0.05, **p < 0.01, ***p < 0.001, ****p < 0.0001. One-tailed Student’s *t* test. **j** The pairwise contingency tables showing interrelationships of each pair of variables among genome-wide insulator strength, promoter activity, and enhancer and looping strength. **k** The optimal structure of relationships among genome-wide insulator strength, promoter activity, enhancer and looping strength during human epidermal differentiation learned by Bayesian networks. **l** Directional CTCF looping underlies stochastic monoallelic *Pcdh α* and *βγ* gene expression and balanced promoter usage. In particular, stochastic and monoallelic CTCF-mediated directional chromatin looping underlies activation of one and only one variable promoter in each chromosome in the *Pcdhα* cluster, while up to 4 promoters are activated in each chromosome in the *Pcdhβ/γ* clusters. **m** A polymer simulated 3D Hulu (gourd) model of tandem CTCF sites topologically balancing spatial chromatin contacts and enhancer-promoter selection. **n** Mechanistic interpretation of the 3D Hulu model in the context of bidirectional cohesin “loop extrusion” through tandem CTCF sites

We next prepared mice with a deletion of the entire *HS5-1* fragment including the two flanking CBS elements of *HS5-1a* and *HS5-1b* (Fig. 5a–e and Additional file 1: Figure S12a,b). We found that the long-distance chromatin interactions between the *HS7* enhancer and the 5′ isoforms of the *Pcdhβ* cluster are significantly increased upon the *HS5-1* deletion (Fig. 5a–c). In addition, the expression levels of the 5′ isoforms of the *Pcdhβ* cluster are also significantly increased (Fig. 5e). This suggests that the two *HS5-1* CBS elements function as an insulator to block the activity of the *HS7* enhancer, which contains no CBS, from aberrantly activating the *Pcdhβ* promoters. As a control, we inverted in situ the same *HS5-1* fragment including the two reverse-oriented CTCF sites in mice in vivo (Fig. 5a and Additional file 1: Figure S12a,b)*.* In contrast to the *HS5-1* deletion, neither *HS7* chromatin looping interactions with nor expression levels of the 5′ isoforms of the *Pcdhβ* cluster are significantly increased (Fig. 5b, d, f). These remarkable differences between deletion and inversion of *HS5-1* clearly show that the two endogenous *HS5-1* CBS elements function as an insulator to block the *HS7* enhancer from aberrantly activating the *Pcdhβ* gene expression, and its insulation activity is orientation-independent in vivo, consistent with the insertions of exogenous CBS elements of either orientation in cell lines in vitro (Fig. 1 and Additional file 1: Figures S3-S7).

To further investigate whether this is true for the *β-globin* cluster, we next inserted a pair of reverse-forward CBS elements (designated “RF2” to be distinguished from the first “RF” in Additional file 1: Figure S5) into the location between the five globin promoters and the *HS2* enhancer, which also contains no CBS (Fig. 5g). ChIP-seq confirmed the binding of CTCF/cohesin to the inserted CBS pair but not its mutant sites (Fig. 5g). QHR-4C experiments with either the *HS2* enhancer or the *HBG2* promoter as a viewpoint demonstrated a significant decrease of the *β-globin* enhancer-promoter interactions (Fig. 5h and Additional file 1: Figure S13a). Consistently, the expression levels of all *β-globin* genes are significantly decreased, and the decrease is rescued by CBS mutations (Fig. 5i).

QHR-4C with *5’HS5* or *3’HS1* as a viewpoint, which contains a CBS element located outside of and beyond the *β-globin* enhancer and promoter regions, respectively, revealed opposite chromatin looping interactions with the inserted reverse-forward CBS pair (Additional file 1: Figure S13b-d). Thus, CBS elements, if inserted between enhancers and promoters with no CBS, also function as insulators by forming long-distance chromatin looping interactions with CBS elements located in the endogenous genome outside of and beyond respective enhancer and promoter regions. Finally, we inserted various combinations of CBS elements upstream and/or downstream of the *HS7* enhancer, which contains no CTCF site, of the *Pcdhα* cluster and found that the inserted CBS elements block long-distance chromatin interactions of the *HS7* enhancer (Additional file 1: Figure S13e-g). In conjunction with the data of the endogenous *Pcdh* CBS deletion, we conclude that CBS elements function as insulators for enhancers with no CBS.

### Genome-wide CTCF sites function as insulators

To see whether genome-wide CTCF-bound CBS elements function as insulators for enhancers, we analyzed developmental plasticity of insulators, promoters, and enhancers during human epidermal differentiation by Bayesian networks learned by the max-min hill-climbing algorithm [45] using categorized factors (Fig. 5j) inferred from previously published capture Hi-C data [46]. Remarkably, we find a direct inverse relationship between insulator strength and promoter activity (Fig. 5k). Moreover, insulators also regulate promoter activity indirectly through enhancers by perturbing the looping strength of their spatial chromatin contacts (Fig. 5k). Thus, these Bayesian network analyses, in conjunction with our deletion and inversion experiments in mice in vivo (Figs. 2, 3, 4, and 5), suggest that CTCF-bound CBS elements function as insulators through directional chromatin looping across the human genome.

## Discussion

Considerable progress has been made in understanding the stochastic expression of large repertoires of gene clusters by spatial regulation of chromatin contacts [12, 28, 33, 47]. In particular, the allelic insulation (Fig. 5l) by CTCF-mediated directional looping may be epigenetically regulated by methylation of CBS elements [35, 48]. Some CBS elements, such as the boundary *Pcdh HS5-1b* site, contain no CpG dinucleotides [35]. Consequently, the *HS5-1b* site has constitutive and cell-invariant CTCF/cohesin occupancy and functions as a chromatin insulator for the downstream *Pcdhβ* genes (Fig. 5l). Other CTCF sites are regulated by DNA methylation and have a cell-specific pattern of the CTCF/cohesin occupancy in single neurons [28]. For example, each CBS within the alternate variable promoter of members of the *Pcdh α*, *β*, and *γ* clusters contains a CpG dinucleotide that is methylated in specific subpopulations of neurons in the brain [28, 35, 49]. Therefore, the 5′ boundary CBS element of each *Pcdh* loop domain is cell-specific and distinct for single neurons, thus functions as a chromatin insulator for its respective upstream genes (Fig. 5l). Consistently, our computational modeling suggests that members of the three *Pcdh* families are expressed monoallelically in individual neurons (Additional file 1: Figures S1 and S9) [40].

This explains the long-standing puzzle of stochastic *Pcdhα* monoallelic expression in single cells (Additional file 1: Figure S1) [39]. Specifically, for any unmethylated promoter CBS element, it forms long-distance chromatin contacts with downstream enhancers and therefore is activated through chromatin looping. These chromatin looping interactions function as an insulator for all of its upstream *Pcdhα* promoters, resulting in inactivation or silencing of them in each chromosomal allele (Fig. 5l). In addition, all of its downstream *Pcdhα* promoters are not activated because by mathematical definition, if any downstream promoter is activated by enhancer looping, none of its upstream promoters could be activated (Fig. 5l). Similarly, for the more complex regulation of the *Pcdh β* and *γ* clusters, our genetic experiments demonstrate that they are topologically regulated by the tandem CTCF sites within the downstream super-enhancer (Figs. 2 and 3), as explained in the Hulu model of topological gene regulation (Fig. 5m). Therefore, only one isoform of the *Pcdhα* cluster (Additional file 1: Figure S1a,b) and up to 4 isoforms of the *Pcdh β* and *γ* clusters (Additional file 1: Figure S9a) are expressed from each chromosomal allele in individual neurons in the brain (Fig. 5l).

We posit a general mechanism for the Hulu model by which tandem directional CTCF sites function as topological insulators in the context of the cohesin “loop extrusion” (Fig. 5m, n). Because each CBS element has permeability for cohesin sliding [20], continuous active chromatin loop extrusion by cohesin in an ATP-dependent manner bridges inner convergent CTCF sites first (Fig. 5n). After sliding through the inner proximal CTCF sites, cohesin will then stall at the intermediate tandem CTCF sites (Fig. 5n). Finally, cohesin will reach the outer distal CTCF sites (Fig. 5n). Based on our experimental observation and mathematical prediction in the clustered *Pcdh* and *Ig* genes, the two “heads” of cohesin are stalled or anchored at CTCF sites in the two arrays of convergent tandem CTCF sites, resulting in long-range chromatin interactions between proximal-proximal CBS elements as well as between distal-distal CBS elements (Fig. 5m, n). In other words, two “heads” of cohesin complex anchor proximal-proximal or distal-distal CBSs through continuous active loop extrusion of chromatin fibers which are asymmetrically blocked by permeable CTCF insulators. The functional consequences of these interactions caused by tandem CTCF insulators are the decreased proximal chromatin interactions and increased distal chromatin interactions. Thus, tandem directional CTCF sites function as topological insulators to balance higher-order chromatin contacts and promoter choice, eliminating bias of spatial chromatin accessibilities between proximal and distal promoters by remote enhancers.

Overwhelming evidence suggests that the function of insulators is orientation-independent, but the chromatin looping of CBS elements is directional [7, 11, 12, 14, 24, 33, 50]. CTCF mediates specific directional loop formation through asymmetric anchoring of the ring-shaped cohesin complex, which slides along chromatin fibers to actively extrude loops [3, 12, 18, 19, 33, 51]. Our data are consistent with the predominant chromatin interactions between forward-reverse CBS pairs [11, 12]. In addition, there are numerous cases of tandem CTCF sites across mammalian genomes [12, 16, 33, 52]. Since the binding of CTCF to genome-wide CBS elements is not static but rather dynamic [9, 10] and there is variable permeability of CTCF extrusion barriers [20], this suggests that cohesin slides through the proximal CTCF sites within tandem CBS arrays to more distal sites (Fig. 5n). Curiously, our computational simulation in silico and genetic deletion in vivo revealed that tandem-arrayed CBS elements ensure balanced usage of associated promoters in specific and equal spatial chromatin contacts in general. Thus, our data on *Pcdh*, *β-globin*, and *Igh* clusters suggest that directional CTCF chromatin looping between convergent CBS elements underlies insulator function and that tandem CTCF sites ensure balanced promoter spatial accessibility in the 3D genome folding and regulation. However, since there are numerous gene clusters and hundreds of thousands CTCF sites in mammalian genomes, whether all tandem CTCF sites function in a similar manner in vivo waits further studies.

## Conclusion

In the present study, we show by CRISPR DNA-fragment editing, in conjunction with mathematic modeling and chromosome conformation capturing, that tandem directional CTCF sites function as topological insulators to enhance long-distance chromatin interactions with distal CTCF sites and to balance promoter-enhancer selections. Specifically, ectopic and endogenous CTCF sites function as insulators in an orientation-independent manner through CTCF-mediated directional chromatin looping. In addition, in combination with computational simulations of cohesin “two-headed” chromatin loop extrusion, we demonstrate that tandem CTCF sites ensure proper spatial accessibility of distal promoters by remote enhancers and balanced usage of target promoters. Finally, we report that tandem CTCF sites regulate long-distance chromatin looping in the mammalian genome in a topological manner.

## Methods

### Cell culture

Human endometrial HEC-1-B cells (ATCC) were cultured in MEM medium (Hyclone), supplemented with 10% (v/v) FBS (Gibco), 2 mM glutamine (Gibco), 1 mM sodium pyruvate (Sigma), and 1× penicillin-streptomycin (Gibco). Human K562 and mouse Neuro-2A cells (ATCC) were cultured in DMEM medium (Hyclone) supplemented with 10% (v/v) FBS and 1× penicillin-streptomycin. Cells were maintained at 37 °C in a humidified incubator containing 5% (v/v) of CO_2_ and were passaged every 3 days.

### In vitro transcription of sgRNA pairs and Cas9 mRNA for microinjection

The preparation of sgRNA pairs and Cas9 mRNA was recently described [38]. Briefly, to obtain sgRNAs for microinjection of zygotes, we performed in vitro transcription using DNA templates generated by PCR with a forward primer containing a T7 promoter followed by targeting sequences and a common reverse primer. In vitro transcription was performed with the MEGAshortscript Kit (Life Technologies) using T7 polymerase by incubating at 37 °C for 5 h. The template DNA was removed by digestion with DNaseI. The transcribed sgRNAs were purified with the MEGAclear Kit (Life Technologies) and eluted in TE buffer (0.2 mM EDTA). The sequences of primers used for preparing sgRNAs were listed in Additional file 2: Table S1.

To obtain Cas9 mRNA for the microinjection of zygotes, the Cas9 coding sequence was cloned into pcDNA3.1 plasmid under the control of the T7 promoter. The plasmid was then linearized by XbaI and used for in vitro transcription with the mRNA transcription system according to the manufacturer’s instructions (Life Technologies). After digestion of the DNA template, the transcribed Cas9 mRNA was purified with the MEGAclear Kit (Life Technologies).

### Generation of the CBS deletion and inversion mice by CRISPR DNA-fragment editing

Mice were maintained at 23 °C in a 12-h (7:00–19:00) light and 12-h (19:00–7:00) dark schedule in an SPF mouse facility. For each CRISPR deletion or inversion of CBS elements, Cas9 mRNA (100 ng/μl) and a pair of sgRNAs (50 ng/μl each) targeting the region flanking the CBS elements were injected into the cytoplasm of one-cell embryos of the C57BL/6 mice. After recovering for 2 h at 37 °C incubator, the embryos were then implanted into the oviducts of the pseudo-pregnant ICR mice. The newborn F0 mice (Additional file 2: Table S2) were then screened for targeted deletions or inversions by PCR using specific primer pairs (Additional file 2: Table S1). The amplified PCR products were then cloned and confirmed by Sanger sequencing. The F0 mice with targeted deletions or inversions were maintained and crossed to obtain F1 mice. F1 mice were genotyped again for heterozygous deletion or inversion. Heterozygous F1 mice were then crossed to obtain homozygous F2 mice. For all of the 5C and RNA-seq experiments, only the wild-type littermates were used as controls.

### Single-cell RNA-seq

Single-cell RNA-seq experiments were performed as previously described [40]. Briefly, for neurons, the P0 mouse brain was dissected, and the tissue from the cerebral cortex was digested with 0.013% of collagenase in Neurobasal Medium (Gibco) at 37 °C for 3 min. The collagenase was neutralized by adding an excess amount of Neurobasal Medium. A single-cell suspension was made by gentle pipetting and then filtered through 100-μm cell strainers (BD Biosciences). For HEC-1-B cells, trypsin was added to the culture dish, and the single cells were suspended in the culture medium. Single cells were then picked under the microscope by using a microcapillary pipette into the thin-walled PCR tube containing 2 μl of cell lysis buffer, 1 μl of oligo-dT primer, and 1 μl of dNTP mix. After reverse transcription, the cDNA was pre-amplified by PCR. The cDNA library was then purified, tagmented, and ligated with adapters using the Nextera XT DNA Library Preparation kit (Illumina FC-131-1096). Finally, the adapter-ligated fragments were further amplified by PCR and purified with AMPure XP beads (Beckman). The single-cell RNA-seq libraries were pooled and sequenced using an Illumina Hiseq 2500 platform.

### Plasmid construction

The plasmids of sgRNAs for cell transfection experiments were constructed as previously described [37, 38]. Briefly, pairs of complementary oligonucleotides for generating sgRNAs (Additional file 2: Table S1) were annealed with 5′ overhangs of “ACCG” and “AAAC,” and cloned into a BsaI-linearized pGL3 vector under the control of the U6 promoter. To insert CBS elements into distinct genomic regions, circular donor plasmids with about 2-kb homologous arms flanking the inserted sequence were used as donors for CRISPR-based homologous recombination. To construct donor plasmids, we amplified the CBS elements, as well as the genomic sequences flanking the insertion site by PCR. CBS elements and the two homologous arms with 20 bp of overlapping sequences were jointed together with the EcoRI and HindIII digested Puc19 vector using the multi-fragment recombination system (Vazyme). All of the plasmids constructed were confirmed by Sanger sequencing. The primer sequences used for the construction of sgRNAs and the donor plasmids were shown in Additional file 2: Table S1.

### Screening CBS insertion and deletion single-cell clones by CRISPR DNA-fragment editing

Generation of the CRISPR single-cell clones with CBS element insertions and deletions was performed as previously described [12, 38]. Briefly, cells were transfected with a plasmid mix using Lipofectamine 3000 reagents (Thermo) in a 12-well plate. For CBS insertions and mutations, Cas9 (0.3 μg) and donor plasmids (0.5 μg) were co-transfected with one sgRNA construct (0.2 μg) targeting the insertion site. For CBS deletions, Cas9 plasmids (0.4 μg) were co-transfected with two sgRNA constructs (0.3 μg each) targeting the two ends of the deletion fragments. The sgRNA constructs contained a puromycin-resistant gene which can be used for selection. Forty-eight hours after transfection, puromycin (Sigma) was added to the culture medium at a final concentration of 2 μg/ml. The culture medium was replaced every day with puromycin for a total of 3 days. Puromycin was then removed, and cells were cultured in normal culture medium for 2 days. The cells were then suspended into a single-cell solution and plated into 96-well plates at the concentration of about one cell per well. Two weeks after plating, single-cell clones were marked manually under a microscope and replaced with fresh culture medium. Four weeks after plating, the single-cell clones were screened for insertion, mutation, or deletion by PCR. At least two individual clones for each insertion, mutation, or deletion were obtained and analyzed. We screened for a total of 1948 single-cell clones, and 80 homozygous clones were obtained and analyzed (Additional file 2: Table S3). Single-cell clones for each editing were confirmed by Sanger sequencing. The primers used for genotyping were listed in Additional file 2: Table S1.

### Targeted blocking of CTCF sites by dCas9

We used a well-established method to block CBS functions by dCas9 [53–55]. We first mutated sequences encoding the RuvC and HNH domains of Cas9 to generate a pcDNA3.1 plasmid encoding a catalytically dead Cas9 (dCas9) which lacks the endonuclease activity. The plasmid backbone contains a puromycin-resistance gene which is suitable for puromycin selection. In addition, we chose the sgRNA sequence to target module 2 and module 3 of CTCF sites according to the molecular structures of CTCF-DNA complexes [56, 57]. The sgRNA expression plasmids were constructed by annealing two overlapping primers and inserting the annealed dsDNA into the plasmid backbone as previously described [37]. The primers used for generating sgRNA plasmids were listed in Additional file 2: Table S1.

We make use of the mouse neuroblastoma cell line Neuro-2A as an established model system to investigate the role of CBS in the regulation of the clustered *Pcdh* genes [12]. The Neuro-2A cells cultured to 70% confluency in 6-well plates were transiently transfected with 1.25 μg dCas9 and 1.25 μg sgRNA plasmids using Lipofectamine 3000 transfection reagent (Invitrogen) with the protocol recommended by the manufacturers. We transfected dCas9 with the plasmid targeting Gal4 for the control group. Forty-eight hours after transfection, cells were selected with 2 μg/ml of puromycin diluted in culture medium for 4 days. The survival cells were cultured for another 3 days in normal culture medium without puromycin and harvested for QHR-4C experiments.

### ChIP-seq experiments

ChIP experiments were performed as previously described [35] with modifications. Briefly, 4 × 10^6^ of cells were cross-linked by 1% formaldehyde in 10% FBS/PBS for 10 min at room temperature. Cells were then lysed twice with ice-cold lysis buffer (20 mM Tris-HCl, 2 mM EDTA, 1% Triton X-100, 0.1% SDS, 0.1% sodium deoxycholate, and 1× protease inhibitors, pH 7.5) for 10 min with slow rotations. The lysed cells were then sonicated to obtain DNA fragments of about 200–500 bp using the Bioruptor system (high energy, with working time of 30 s and resting time of 30 s, 30 cycles). After removal of the insoluble debris, the lysate was incubated with specific antibodies against CTCF (07-729; Millipore), RAD21 (ab992; Abcam), or NIPBL (A301-779A; Bethyl Laboratories) and purified by protein A-agarose beads (16-157; Millipore). NIPBL and CTCF ChIP-seq for the *Igh* locus were recently published [58]. ChIP DNA was extracted and prepared for high-throughput sequencing using a DNA library preparation kit for Illumina (NEB). ChIP-seq libraries were sequenced on a HiSeq X Ten platform (Illumina).

### Quantitative high-resolution chromosome conformation capture copy (QHR-4C)

We developed a QHR-4C method to detect genomic elements that are close to any viewpoint of interest with high efficiency and specificity. This method is conceptually similar to UMI-4C and HTGTS [59]. We used this method to study chromatin conformation of the clustered *Pcdh* and *β-globin* loci from as few as 50,000 cells. After the cells were harvested and crosslinked, chromatins within the nuclei were digested in situ by a restriction enzyme. The chromosome conformation is then captured by proximal ligation. After fragmentation by sonication, a linearized amplification step is applied to enrich ligation events associated with a specific viewpoint using a single primer tagged with biotin. The amplified single-stranded biotin-tagged DNA fragments were purified with streptavidin beads and ligated with a staggered adapter. Finally, QHR-4C libraries were generated by PCR.

Compare to the regular 4C, QHR-4C has several advantages. First, the chosen viewpoint is much more flexible in QHR-4C. In the regular 4C, the size of the viewpoint fragments should be at least 200 bp to allow for efficient self-circulating in the second ligation step. In addition, there must be at least one restriction enzyme cutting site within the viewpoint fragment to allow for self-circulation. By contrast, the only requirement for viewpoint selections in QHR-4C is the matching of a linearized amplification primer. Second, the regular 4C could not detect chromatin interactions of the fragments that do not contain the second restriction enzyme cutting site. However, QHR-4C, which does not require the second digestion step, is able to detect these chromatin interactions and allows for better coverage of genomic regions of interests. Third, since the ends of the captured DNA fragments are generated by sonication, the captured dsDNA ends are random and unique, and thus can be used as an identifier for quantifying the long-range chromatin interactions. Finally, multiplexing QHR-4C is much easier than the regular 4C experiments.

Briefly, single cells from various CRISPR single-cell clones and mouse cortical tissues were centrifuged at 500*g* for 5 min, and the pellets were used for QHR-4C experiments. The cell pellets were suspended for crosslinking in 900 μl 2% formaldehyde at room temperature for 10 min. The crosslinking reaction was stopped by adding and mixing with 100 μl of 2 M glycine for a final concentration of 200 mM. The fixed cells were spun down at 800*g* at 4 °C for 5 min and washed twice by suspending briefly in 1 ml ice-cold PBS. Cells were then permeabilized twice with 200 μl ice-cold 4C permeabilization buffer each for 10 min (50 mM Tris-HCl pH 7.5, 150 mM NaCl, 5 mM EDTA, 0.5% NP-40, 1% Triton X-100, and 1× protease inhibitors). After centrifugation, the pellet was resuspended in 73 μl water, 10 μl of 10× DpnII buffer (we used DpnII enzyme as an example, using the recommended buffer for other enzymes), and 2.5 μl of 10% SDS. The reaction was performed at 37 °C for 1 h with constant shaking at 900 rpm. 12.5 μl of 20% Triton X-100 was added into the reaction to quench SDS and incubated at 37 °C for 1 h with shaking at 900 rpm. The cells were then digested in situ overnight at 37 °C with 2 μl of DpnII (10 U/μl) while shaking at 900 rpm. After the inactivation of DpnII at 65 °C for 20 min, the pellets of the nuclei were collected by centrifuging at 1000*g* for 1 min, and the supernatant was removed completely, which ensures the subsequent ligation reaction can be performed in a small volume. Proximity ligation was carried out for 24 h at 16 °C with 1 μl T4 DNA ligase (400 unit/μl) in 100 μl 1× T4 ligation buffer. The ligated product was then reverse cross-linked by heating to 65 °C for 4 h in the presence of 1 μl proteinase K (10 mg/ml) to digest proteins. The DNA was then extracted using phenol-chloroform. One microliter glycogen (20 mg/ml) was added to facilitate DNA precipitation. The precipitated DNA was dissolved in 50 μl water. We sonicated the ligated DNA using the Bioruptor system (with low energy setting at a train of 30-s sonication with 30-s interval for 12 cycles) to obtain DNA fragments ranging from 200 to 600 bp.

After fragmentation, a linearized amplification step is applied to enrich the ligation events associated with a specific viewpoint, using a 5′ biotin-tagged primer (Additional file 2: Table S1) complementary to the viewpoint fragment in 100 μl of PCR system for a total of 60 cycles. This primer should be neither too close to the DpnII site to facilitate the nested PCR at the final amplification step nor too far away from the DpnII site to maximize the product amount. The amplification products were denatured by incubating at 95 °C for 5 min and immediately chilled on ice to obtain ssDNA. The ssDNA was then enriched and purified with Streptavidin Magnetic Beads (Invitrogen) according to the manufacturer’s instructions.

The ssDNA on beads was then ligated in 15 μl ligation buffer with 0.1 μM of adapters (Additional file 2: Table S1) at 16 °C for 24 h. We chose the adapter sequence that matched the 3′ end of the Illumina P7 sequence so that one PCR step can produce sequencing libraries. The adapters were generated by annealing two complementary primers in annealing buffer (25 mM NaCl, 10 mM Tris-HCl pH 7.5, 0.5 mM EDTA). After ligation, free adapters were removed by washing the beads twice with the B/W buffer (5 mM Tris-HCl, 1 M NaCl, 0.5 mM EDTA, pH 7.5). The DNA on beads was resuspended in 10 μl water. Finally, the QHR-4C libraries were generated by one-step PCR amplification (94 °C, 2 min; 94 °C, 10 s; 60 °C, 15 s; 72 °C, 1 min for 19 cycles; and a final extension at 72 °C, 5 min) with captured DNA on beads as the template and a pair of PCR primers. The forward primer matches the Illumina P5 and the viewpoint sequence adjacent to the DpnII site with barcodes, and the reverse primer matches Illumina P7 with indexes (primer sequences are listed at Additional file 2: Table S1). The PCR products were purified with a PCR purification kit (Qiagen). About 100 QHR-4C libraries with different combinations of barcodes and indexes were pooled and sequenced on an Illumina HiSeq X Ten platform. All of the QHR-4C experiments for each CRISPR clone and CRISPR mouse lines were performed with two biological replicates.

### Circularized chromosome conformation capture

The circularized chromosome conformation capture (4C) experiments were performed as previously described [12, 14]. Briefly, cells were counted, and about 2 × 10^6^ cells were used for each 4C experiment. After cross-linking with 2% formaldehyde, cells were lysed twice with cold lysis buffer, digested with DpnII, and ligated with T4 DNA ligase. The ligated samples were purified using the High-Pure PCR Product Purification kit (Roche). The 4C-seq libraries were generated by PCR using a high-fidelity DNA polymerase (Vazyme). All of the 4C experiments were performed with biological replicates. 4C-seq libraries were sequenced on the HiSeq X Ten platform. 4C primers used were listed in Additional file 2: Table S1.

### Chromosome conformation capture carbon copy

Chromosome conformation capture carbon copy (5C) experiments were performed as previously described [60, 61]. Briefly, a total of 46 forward and 46 reverse primers covering the mouse *Pcdh α* and *β* clusters were designed by My5C tools (http://my5c.umassmed.edu) [62]. These primers are a subset of the 5C primer set covering all three *Pcdh* gene clusters [63]. All forward primers contain a 5′ end T7 universal primer sequence (CGGTA ATACG ACTCA CTATA GCC) preceding a unique sequence which is followed by AAG at the 3′ end. All reverse primers contain CTT at 5′ end followed by a unique sequence and a complementary T3 universal sequence (TCCCT TTAGT GAGGG TTAAT A). All reverse primers were 5′-phosphorylated.

### Generation of 5C libraries for sequencing

The P0 mouse cortical tissues were dissociated to obtain single-cell suspension as described above in the single-cell RNA-seq experiments. A total of 10^7^ cells were cross-linked and digested with HindIII (NEB). After inactivating HindIII, the digested DNA was ligated with T4 DNA ligase and purified. As a control, DNA of six bacterial artificial chromosomes (BACs) covering the three *Pcdh* clusters was also digested, ligated, and purified. The purified mouse cortical DNA was mixed with 1 μg of salmon sperm DNA (Sigma). The control BAC DNA (5 ng) was mixed with 1.5 μg of salmon sperm DNA. These samples were then each mixed with 1.7 fmol of each 5C primer and 1 μl of 10 × 5C annealing buffer (20 mM Tris-acetate pH 7.9, 50 mM potassium acetate, 10 mM magnesium acetate, 1 mM DTT) in a total volume of 10 μl and denatured at 95 °C for 5 min. Annealing was performed by incubation at 48 °C for 16 h. The annealed DNA was ligated by adding Taq DNA ligase (NEB) in the 5C ligation buffer (25 mM Tris-HCl pH 7.6, 31.25 mM potassium acetate, 12.5 mM magnesium acetate, 1.25 mM NAD, 12.5 mM DTT and 0.125% Triton X-100). The ligation reaction was performed for 1 h at 48 °C followed by incubation for 10 min at 65 °C to stop the ligation reaction. The ligated products were amplified by PCR with Illumina primer pairs. The amplified libraries were purified with a PCR purification kit (QIAGEN) for high-throughput sequencing.

### 5C reads mapping

The 5C libraries were sequenced with the 90-bp pair-end mode by the Hi-seq 2500 platform of Illumina. All 5C experiments were performed with two biological replicates. The read depth of each sample was equal to about 2 million (Additional file 2: Table S4). Pearson correlation coefficients between the two biological replicates range from 0.967081 to 0.99251 (Additional file 2: Table S5). We used 56-bp reads for mapping. Each of the paired-end reads was independently mapped using the local mapping mode of Bowtie2 with default parameters. Only both of the paired-end reads uniquely mapped to a single 5C interaction were used for downstream analyses. We found that about 96% of paired-end reads can be uniquely mapped (Additional file 2: Table S4). The read count was then normalized to 1 million for each sample to correct the difference in sequencing depth.

### 5C bias correction

Bias may be introduced in many steps in 5C experiments including, but not limited to, differences in the crosslinking efficiency, differences in restriction enzyme digestion efficiency, differences in ligation efficiency, differences in 5C primer and PCR amplification efficiency, and differences in DNA sequencing efficiency. All of these potential biases are shared by all experimental groups as we used the same sets of primers and investigated the same genomic region. As a result, the bias can be partially neutralized as we focused on the differences between each sample. In addition, we performed BAC control experiments to reduce 5C primer and PCR amplification bias. Finally, we filtered primers by a statistical method known as Loess.

### Locally estimated scatterplot smoothing

Locally estimated scatterplot smoothing (Loess) locally fits the response *y*_*i*_ (5C interaction frequency) to the predictor *x*_*i*_ (genomic distance) for *i* ∈ [1, *n*] by a function from a specific parametric class, say polynomials of degree 1 or 2, which provide an estimate $\hat{g}(x)$. A function ${w}_{\hat{x}}(x)$ with local support is used to weight the predictors around $\hat{x}$.

$$ {w}_{\hat{x}}(x)=\left\{\begin{array}{c}{\left(1-{\left(\frac{\left|x-\hat{x}\right|}{d}\right)}^3\right)}^3,\left|x-\hat{x}\right|\le d,\\ {}0,\left|x-\hat{x}\right|>d,\end{array}\right. $$

where *d* is the distance from $\hat{x}$ to the ⌈*αn*⌉th closest predictor in {*x*_1_, *x*_2_, ⋯, *x*_*n*_}, and *α* is the percentage of data points used to calculate the response for $\hat{x}$. Under the assumption that the errors ${\epsilon}_i:= {y}_i-\hat{g}\left({x}_i\right)$ are independent Gaussian random variables with 0 means and constant variances *σ*^2^, Loess does weighted least squares, i.e., $\hat{g}\left({\boldsymbol{x}}_{\hat{x}}\right)=X{\left({X}^T WX\right)}^{-1}{X}^TW{\boldsymbol{y}}_{\hat{x}}$, where ${\boldsymbol{x}}_{\hat{x}}={\left({x}_{i_1},{x}_{i_2},\cdots, {x}_{i_m}\right)}^T$ such that $\left\{{x}_{i_1},{x}_{i_2},\cdots, {x}_{i_m}\right\}=\left\{{x}_i|{w}_{\hat{x}}\left({x}_i\right)>0,1\le i\le n\right\}$, ${\boldsymbol{y}}_{\hat{x}}$ = $\left({y}_{i_1},{y}_{i_2},\cdots, {y}_{i_m}\right)$, $W=\operatorname{diag}\left({w}_{\hat{x}}\left({x}_{i_1}\right),{w}_{\hat{x}}\left({x}_{i_2}\right),\cdots, {w}_{\hat{x}}\left({x}_{i_m}\right)\right)$, and *X* depends on the parametric class used for the local regression. In the case of a polynomial of degree 2:

$$ X=\left(\begin{array}{ccc}1& {x}_{i_1}& {x}_{i_1}^2\\ {}1& {x}_{i_2}& {x}_{i_2}^2\\ {}\vdots & \vdots & \vdots \\ {}1& {x}_{i_m}& {x}_{i_m}^2\end{array}\right). $$

Denote *L* ≔ *X*(*X*^*T*^*WX*)^−1^*X*^*T*^*W*. Then, the covariance matrix of the errors ${\boldsymbol{\epsilon}}_{\hat{x}}:= {\boldsymbol{y}}_{\hat{x}}-\hat{g}\left({\boldsymbol{x}}_{\hat{x}}\right)$ is ${\sigma}^2\left(I-L\right){\left(I-L\right)}^T\approx \frac{{\boldsymbol{\epsilon}}_{\hat{x}}^T{\boldsymbol{\epsilon}}_{\hat{x}}\left(I-L\right){\left(I-L\right)}^T}{\mathrm{tr}\left(I-L\right){\left(I-L\right)}^T}$, which gives the standard deviation SD_*i*_ for each data point.

### Primer filtering

We performed data correction using locally estimated scatterplot smoothing (Loess) to calculate *Z* scores (a measurement of the number of standard deviations a data point is from the average value) of each 5C chromatin interaction. First, we calculated the global average relationship $\hat{g}$ between the interaction frequency and genomic distance via Loess smoothing for each sample. We used Loess [64] implemented in R to calculate the *Z* score ${Z}_i:= \left({y}_i-\hat{g}\left({x}_i\right)\right)/{\mathrm{SD}}_i$ with default setting and the span of *α* = 0.01. In this equation, *y*_*i*_ and *x*_*i*_ are the interaction frequency and genome distance of pair *i*, respectively. In addition, SD_*i*_ is the standard deviation of ${y}_i-\hat{g}\left({x}_i\right)$. The overall interaction profile of each primer is then compared to the global average. If the individual Loess of a primer is higher or lower than 0.85 of the global average, it is flagged as problematic. If a primer is flagged in more than 40% of the datasets from all samples, it is removed from the downstream analyses from all datasets [65–67]. Using this threshold, we removed 7 primers (mpcdh-for-2, mpcdh-for-8, mpcdh-for-21, mpcdh-rev-6, mpcdh-rev-12, mpcdh-rev-17, mpcdh-rev-25) from the downstream analyses.

### Singleton removal

In 5C experimental data, there are instances that 5C interactions resulting from aberrant PCR amplifications were much higher than neighboring interactions by more than an order of magnitude. These abnormal interactions may be caused by PCR over-amplification, the so-called PCR “blowouts” or abnormal singletons. To remove these singletons, we calculated the *Z* score for each 5C interaction. If the *Z* score of a 5C singleton is larger than 12, the singleton is removed [65–67]. In total, three singletons (mpcdh_for_14 - mpcdh_rev_30, mpcdh_for_25 - mpcdh_rev_39, and mpcdh_for_28 - mpcdh_rev_22) have been removed.

After data correction, we normalized 5C interactions by dividing the BAC sample. The mean ratio of two biological replicates is shown as heatmaps. To compare the interaction profiles, the log2 ratio between mutant and wild-type groups is calculated and shown as heatmaps.

### RNA-seq experiments

RNA-seq experiments were performed as previously described [12] with modifications. Briefly, total RNA from mouse cortical tissues or cultured cells was extracted using TRIzol reagents (Life Technologies) following the manufacturer’s instructions. Total mRNA was prepared from 1 μg total RNA using poly(A) mRNA magnetic isolation reagents (NEB) and fragmented at 94 °C for 15 min. RNA was then reverse-transcribed into cDNA with random primers. After end repairing and A-tailing, cDNA was ligated with adapters and amplified by PCR with Illumina sequencing primers. All RNA-seq experiments were performed with biological replicates. RNA-seq libraries were sequenced on a HiSeq X Ten platform.

### High-throughput sequencing and data analyses

High-throughput analyzing pipelines were the same as previously described [12, 35] with some modifications. Briefly, reads that passed the Illumina quality filter were considered for alignments. For 4C-seq data, reads were aligned to the reference human (GRCh37/hg19) or mouse (NCBI37/mm9) genome using the Bowtie2 program. The reads per million (RPM) value was calculated using the r3Cseq program (version 1.20) in the R package (version 3.3.3). For QHR-4C data, duplicated paired-end reads were removed by FastUniq (version 1.1) program, and only the unique reads were used for analyses using the Bowtie and r3Cseq program. For ChIP-seq data analyses, reads were mapped to the reference genome (human GRCh37/hg19 or mouse NCBI37/mm9) or the modified genome with insertions using the Bowtie2 program. Peaks were called by the MACS program [68] (version 1.4.2) with a cutoff *p* value of 10^−5^. For RNA-seq and single-cell RNA-seq data, reads were aligned using Hisat2 (version 2.0.4) to the human genome (GRCh38/hg38) or mouse genome (GRCm38/mm10), and the FPKM value was calculated using the Cufflinks program (version2.1.1).

### Maximum likelihood modeling of *Pcdh* stochastic expression

Since single-cell RNA-seq data of each neuron are resulted from the combined expression of two sets of paternal and maternal chromosomes, upon the assumption that two chromosomal sets express independently, we first decomposed the RNA-seq data of single cells from the anterior lateral motor and primary visual cortices [40] into the expression of each chromosomal sets.

Let *G* be the total number of considered genes (for example, *G* = 12 in the mouse *Pcdhα* cluster). Define the whole gene set as $\mathcal{G}=\left\{g|1\le g\le G\right\}$. Because there are 81.56% and 86.64% single cells from anterior lateral motor and primary visual cortices express no more than 2 *Pcdhα* isoforms (Additional file 1: Figure S1), respectively, we assume that the *Pcdhα* cluster on a single chromosomal allele expresses at most *H* genes (*H* = 2 here in the *Pcdhα* cluster). Define ${\mathbbm{G}}_H:= \left\{\mathcal{S}|\mathcal{S}\subset \mathcal{G},\left|\mathcal{S}\right|\le H\right\}$, where $\left|\mathcal{S}\right|$ is the total number of elements in the set $\mathcal{S}$, as the set of all the subsets of $\mathcal{G}$ that contain less than *H* elements. In other words, ${\mathbbm{G}}_H$ gives all possible gene sets that can be expressed from a single chromosomal allele.

Define the Cartesian product ${\mathbbm{G}}_H^2:= \left\{\left(\mathcal{S},\mathcal{T}\right)|\mathcal{S},\mathcal{T}\in {\mathbbm{G}}_H\right\}$ as all possible combinatorial expression sets from both chromosomal alleles. For $\mathcal{R}\subset \mathcal{G}$, let ${N}_{\mathcal{R}}$ be the number of single cells that express the gene set $\mathcal{R}$. Define ${\mathbbm{G}}_{H,\mathcal{R}}^2:= \left\{\left(\mathcal{S},\mathcal{T}\right)|\left(\mathcal{S},\mathcal{T}\right)\in {\mathbbm{G}}_H^2,\mathcal{S}\cup \mathcal{T}=\mathcal{R}\right\}$. ${\mathbbm{G}}_{H,\mathcal{R}}^2=\varnothing$ if and only if $\left|\mathcal{R}\right|>2H$. $\mid {\mathbbm{G}}_{H,\mathcal{R}}^2\mid >1$ means that there are more than one way of the *Pcdhα* isoforms to be expressed from both chromosomal alleles to achieve the total expressed gene set $\mathcal{R}$. Define ${N}_{\mathcal{S},\mathcal{T}}$ as the number of single cells that the first chromosomal allele expresses gene set $\mathcal{S}$ and the second chromosomal allele expresses gene set $\mathcal{T}$. ${N}_{\mathcal{S},\mathcal{T}}$ is hidden. By definition, ${N}_{\mathcal{R}}={\sum}_{\left(\mathcal{S},\mathcal{T}\right)\in {\mathbbm{G}}_{H,\mathcal{R}}^2}{N}_{\mathcal{S},\mathcal{T}}$. Define ${\left({N}_{\mathcal{S},\mathcal{T}}\right)}_{{\mathbbm{G}}_H^2}:= \left\{{N}_{\mathcal{S},\mathcal{T}}|\left(\mathcal{S},\mathcal{T}\right)\in {\mathbbm{G}}_H^2\right\}$ and ${\left({N}_{\mathcal{R}}\right)}_{\mathcal{G}}:= \left\{{N}_{\mathcal{R}}|\mathcal{R}\subset \mathcal{G}\right\}$ under the independent assumption ${P}_{{\mathbbm{G}}_H^2}\left(\mathcal{S},\mathcal{T}\right)={P}_{{\mathbbm{G}}_H}\left(\mathcal{S}\right){P}_{{\mathbbm{G}}_H}\left(\mathcal{T}\right)$, where ${P}_{{\mathbbm{G}}_H^2}$ and ${P}_{{\mathbbm{G}}_H}$ are distributions (probability measures) on ${\mathbbm{G}}_H^2$ and ${\mathbbm{G}}_H$. We choose ${P}_{{\mathbbm{G}}_H}$ which maximizes the likelihood $P\left[{\left({N}_{\mathcal{R}}\right)}_{\mathcal{G}}|{P}_{{\mathbbm{G}}_H}\right]$. This is achieved by alternately maximizing the complete likelihood $P\left[{\left({N}_{\mathcal{S},\mathcal{T}}\right)}_{{\mathbbm{G}}_H^2}|{P}_{{\mathbbm{G}}_H}\right]$ over ${P}_{{\mathbbm{G}}_H}$, and calculating the conditional expectation [69] as ${E}_{\left.{\left({N}_{\mathcal{S},\mathcal{T}}\right)}_{{\mathbbm{G}}_H^2}\right|{\left({N}_{\mathcal{R}}\right)}_{\mathcal{G}},{P}_{{\mathbbm{G}}_H^2}}{\left({N}_{\mathcal{S},\mathcal{T}}\right)}_{{\mathbbm{G}}_H^2}$. To be exact, do ${P}_{{\mathbbm{G}}_H}\left(\mathcal{S}\right)\propto {\sum}_{\mathcal{T}\in {\mathbbm{G}}_H}{N}_{\mathcal{S},\mathcal{T}}$ and ${N}_{\mathcal{S},\mathcal{T}}\propto {P}_{{\mathbbm{G}}_H}\left(\mathcal{S}\right){P}_{{\mathbbm{G}}_H}\left(\mathcal{T}\right)$ until convergence. Note that the second equation is done under the constraint ${N}_{\mathcal{R}}={\sum}_{\left(\mathcal{S},\mathcal{T}\right)\in {\mathbbm{G}}_{H,\mathcal{R}}^2}{N}_{\mathcal{S},\mathcal{T}}$. We initially assume that ${N}_{\mathcal{S},\mathcal{T}}={N}_{\mathcal{R}}/\left|{\mathbbm{G}}_{H,\mathcal{R}}^2\right|$ for $\left(\mathcal{S},\mathcal{T}\right)\in {\mathbbm{G}}_{H,\mathcal{R}}^2$.

### Polymer simulation of tandem-arrayed CTCF sites

We used a method to simulate long-distance chromatin interactions based on cohesin loop extrusion on a coarse-grained DNA fragment [19]. The modeled DNA fragment is divided into roughly equal bins. Long-distance chromatin interactions between one bin and all other bins are determined by their 3D distances according to the polymer simulation of cohesin loop extrusion.

### “Two-headed” cohesin loop extrusion

Cohesin complex may extrude chromatin fiber individually [70] and asymmetrically [71, 72], or may even use the “inchworm” model [72, 73]. For clarity, we assume that cohesin loop extrusion with “two heads” as previously proposed [19]. Cohesin can be loaded stochastically on any location or in a specific position by NIPBL and start to extrude chromatin fibers in opposite directions. The extrusion process is continuous until blocked by oriented CBS which bound CTCF protein in an antiparallel manner [10].

### Coarse-grained polymer simulations

Based on the loop extrusion model, we simulate QHR-4C long-distance chromatin interactions according to the previous polymer modeling method, which is pioneered by the Mirny and Dekker laboratories, and assume the chromatin fiber as a polymer of 10-nm monomers each contains roughly three nucleosomes (about 600 bp) with excluded volume interactions and without topological constraints [19, 74]. We first divide the human *Pcdh* locus (chr5:140160700-140920300 of the GRCh37/hg19 assembly) into *L* = 1266 bins (monomers) each of about 600 bp in length for coarse-grained simulations [19, 75]. Thus, the entire *Pcdh* locus is considered as a polymer containing 1266 monomers. The simulation consists of both 1D (one dimensional) lattice loop extrusion processes and 3D (three dimensional) polymer simulations with molecular dynamics.

### 1D lattice loop extrusion

In the 1D lattice loop extrusion, “two heads” of the cohesin (loop-extrusion factor) independently extrude a DNA loop in opposite directions in an ATP-dependent manner until blocked by CTCF insulators asymmetrically or dropping off from the coarse-grained chromatin fiber (Additional file 1: Figure S7g) [19, 76]. In addition, the cohesin ring cannot pass through each other during extrusion. Finally, CBS can block cohesin sliding in an orientation-dependent manner [11, 18, 19].

The concepts of cohesin separation and processivity are introduced to characterize loop extrusion [19, 20]. Accordingly, cohesin separation is the mean distance between consecutive sliding cohesin complexes on a chromatin fiber, and cohesin processivity *λ* is the mean size of the extruded loops. Specifically, for *L* bins and separation *d*, the number of cohesins on the *Pcdh* locus is calculated as ⌊*L*/*d*⌋. The initial locations of these cohesins are determined according to the loading probabilities inferred from the NIPBL ChIP-seq data. Both heads of a cohesin either occupy the same bin or two adjacent bins with a probability of 0.5 for each. Different cohesins cannot occupy the same bin. At each step, a cohesin may drop off from the chromatin fiber or polymer with the probability 2/*λ*, where *λ* is the processivity. If one cohesin drops off, a new one will be immediately loaded to the polymer according to the loading probabilities from the NIPBL ChIP-seq data but avoiding existing ones. This keeps the number of cohesin complexes unchanged for the *Pcdh* locus. Finally, both “heads” of a cohesin complex can extrude through a bin if it is unoccupied by CTCF or another cohesin.

We determine cohesin loading by calculating the coverage of the NIPBL ChIP-seq for each bin. Eighty percent of cohesins load to the chromatin fiber according to the probabilities proportional to NIPBL coverages of bins, and 20% load randomly [77]. We design the following two methods to calculate CBS permeability or CTCF occupancy for cohesin loop extrusion. The first one is based on ChIP-seq experimental data for CTCF occupancy. The second one is based on dynamic interactions between CTCF and its genomic target sites [9, 10, 56, 57].

### Estimation of permeability of bins based on CTCF ChIP-seq data

Since cohesin accumulates at CBS only when it is occupied by CTCF proteins [78], it has been established that CTCF binding strength of a site can be translated into cohesin permeability of that site [19]. The orientations of bins are determined by CTCF sites within the bins. The CTCF sites are called by the FIMO program [79] from the experimental CTCF ChIP-seq data in the *Pcdh* locus. We first map CTCF ChIP-seq reads to the *Pcdh* cluster by Bowtie2 [80]. The CTCF occupancies (cohesin stalling probability) are called by MACS2 [68]. Each has a fold enrichment value *x*.

If a bin contains CTCF sites in only one orientation, it stalls opposite cohesins with the probability $\mathcal{T}=\frac{1}{1+\exp \left(-\frac{x}{\zeta }-\mu \right)}$ for *x* > 0 and 0 for *x* = 0, where *ζ* = 40, *μ* = 4, and *x* is the CTCF enrichment [19]. If a bin contains CTCF sites in both orientations, it stalls cohesins in both directions with stalling probabilities calculated separately. The CTCF occupancy and cohesin permeability of the *Pcdh* locus in the CBS-inserted clones are estimated similarly according to their ChIP-seq data.

### Estimation permeability of cohesin sliding through oriented CTCF array with no ChIP-seq data available

It was recently reported that CTCF binding to dsDNA is much more dynamic than cohesin and that the residence time of cohesin on DNA fiber is at least 10-fold more than CTCF [9]. The dynamic binding of CTCF to oriented CTCF sites provides hindrance for cohesin sliding [9, 10, 56, 57]. In this scenario, the permeability is calculated as follows. If there are *n* consecutive CTCF sites *c*_1_, *c*_2_, ⋯, *c*_*n*_, from distal to proximal, with a permeability of *p*_1_, *p*_2_, ⋯, *p*_*n*_, respectively, we want to know the mean attempting times *x*_*n*_ for cohesin ring to slide through the entire CBS array from proximal to distal. For the first attempt, the proximal CBS has a probability *p*_*n*_ to allow cohesin ring to pass through. Thus, the cohesin needs *x*_*n* − 1_ attempting times on average to slide through the remaining CBS array *c*_1_, *c*_2_, ⋯, *c*_*n* − 1_. Otherwise, the proximal CBS *c*_*n*_ has the probability (1 − *p*_*n*_) to block cohesin ring passing through. Thus, one attempting time has been used and cohesin still needs *x*_*n*_ attempting times on average to slide through the entire CBS array *c*_1_, *c*_2_, ⋯, *c*_*n*_. In summary:

$$ {x}_n={p}_n{x}_{n-1}+\left(1-{p}_n\right)\left(1+{x}_n\right). $$

Since *x*_0_ = 1, by mathematical induction, ${x}_n={\sum}_{i=1}^n1/{p}_i-n+1$. Then, one obtains the overall permeability 1/*x*_*n*_ of CTCF sites *c*_1_, *c*_2_, ⋯, *c*_*n*_.

### 3D polymer simulations

#### Lennard-Jones (LJ) reduced units

Bins are considered as monomers with diameter *σ* and mass *m*. The Langevin equation:

$$ m\frac{d^2r}{d{t}^2}=-\nabla U-\gamma \frac{dr}{dt}+\sqrt{2{k}_B T\gamma}\eta (t) $$

is rescaled to:

$$ \frac{d^2r}{d{t}^2}=-\nabla U-\alpha \gamma \frac{dr}{dt}+\sqrt{2\alpha \gamma}\eta (t) $$

by LJ reduced units [81] that *m*, *σ*, *k*_*B*_*T*, and (*σ*^2^*m*/*k*_*B*_*T*)^1/2^ are units of mass, distance, energy, and time, respectively, where $\alpha =\frac{\sigma }{{\left(m{k}_BT\right)}^{1/2}}$. We set *m* = 100 Da, *σ* = 1 nm, *T* = 300 K, and *γ* = 0.01 ps^−1^m according to previous reports [19].

#### Bonds in the reduced units

The repulsive potential is defined as previously described [19].

$$ {U}_{\mathrm{REP}}=\mathrm{REPe}\left\{1+{\left(\frac{r\mathrm{REPrmin}}{\mathrm{REP}\mathrm{sigma}}\right)}^{12}\left[{\left(\frac{r\mathrm{REPrmin}}{\mathrm{REP}\mathrm{sigma}}\right)}^2-1\right]/\mathrm{REPemin}\right\}, $$

where REPe = 1.5, $\mathrm{REPrmin}=\sqrt{6/7}$, REPsigma = 1.05, and $\mathrm{REPemin}=\frac{46656}{823543}$. Harmonic bond *U*_HAR_ = *k*(*r* − *d*)^2^ is used between adjacent monomers with *k* = 100 and *d* = 1, and cohesin-bounded monomers with *k* = 25 and *d* = 0.5. The polymer stiffness is described by *U*_STI_ = 2(1 − cos *θ*).

### Langevin velocity Verlet algorithm

The time step *Δt* = 80 ts [19]. The velocities *v*, forces *f*, and positions *r* of monomers are updated by the Langevin velocity Verlet algorithm [82].

$$ v=v+\frac{\varDelta t}{2f}+ b\varDelta w, $$

$$ r=r+ cv, $$

$$ f=f(r), $$

$$ v= av+ b\varDelta w+\frac{\varDelta t}{2f}, $$

where $$ a:= \frac{2-\alpha \gamma \varDelta t}{2+\alpha \gamma \varDelta t} $$, $$ b:= \sqrt{\alpha \gamma \varDelta t/2} $$, and $$ c:= \frac{2\varDelta t}{2+\alpha \gamma \varDelta t} $$.

### Simulation of QHR-4C data process

We simulated long-distance chromatin interaction profiles between a viewpoint of interest and its target genomic regions by coarse-grained modeling. We first transform the experimental contact frequencies from restriction fragments to coarse-grained bins of 600 bp.

Unlike Hi-C and 5C data, 4C data with different viewpoints, even for the same cell types, cannot be compared directly because of their inconsistent scales. Assume viewpoint *i* ∈ [1, *I*] forms *J*_*i*_ valid pairs (*i*, *j*) for *j* ∈ [1, *J*_*i*_]. Let *u*_*ij*_ be the contact frequency of pair (*i*, *j*). We choose *k*_*i*_ for *i* ∈ [1, *I*] and *α* minimizing the geometric standard deviation of $\frac{u_{ij}}{k_i{s}_{ij}^{-\alpha }}$ (the contact frequency decreases with the 1D distance roughly in power law [19])

$$ \mathrm{GSD}:= \exp \left\{\sqrt{\frac{\sum_{i=1}^I{\sum}_{j=1}^{J_i}{\left[\log \left(\frac{u_{ij}}{k_i{s}_{ij}^{-\alpha }}\right)-\beta \right]}^2}{J}\ }\right\}, $$

where *β* is the mean of $\log \left(\frac{u_{ij}}{k_i{s}_{ij}^{-\alpha }}\right)$ and $J:= {\sum}_{i=1}^I{J}_i$. *α* and log*k*_*i*_ solve the linear algebra:

$$ \frac{\partial \log \mathrm{GSD}}{\partial \alpha }=\sum \limits_{i=1}^I\sum \limits_{j=1}^{J_i}2\log {u}_{ij}\left(\log {s}_{ij}-\beta \right)+\alpha \sum \limits_{i=1}^I\sum \limits_{j=1}^{J_i}2\log {s}_{ij}\left(\log {s}_{ij}-\beta \right)+\sum \limits_{i=1}^I\log {k}_i\sum \limits_{j=1}^{J_i}\left(-2\right)\left(\log {s}_{ij}-\beta \right)=0, $$

$$ \frac{\partial \log \mathrm{GSD}}{\partial \log {k}_w}=\sum \limits_{i=1}^I\sum \limits_{j=1}^{J_i}2\log {u}_{ij}\left(-{\delta}_{i,w}+{J}_w/J\right)+\alpha \sum \limits_{i=1}^I\sum \limits_{j=1}^{J_i}2\log {s}_{ij}\left(-{\delta}_{i,w}+{J}_w/J\right)+\sum \limits_{i=1}^I\log {k}_i\sum \limits_{j=1}^{J_i}\left(-2\right)\left(-{\delta}_{i,w}+{J}_w/J\right)=0. $$

Without loss of generality, fix *k*_1_ = 1 to remove the redundancy among the equations of log*k*_*i*_ for *i* ∈ [1, *I*]. Finally, divide *u*_*ij*_ by *k*_*i*_ to obtain comparable 4C contact frequencies.

Since there are data of two biological replicates available, both mean and variance of contact frequencies are calculated for each pair. Finally, pairs with 0 mean contact frequency are excluded from the fitting of 4C simulation by the approach of relative maximum entropy.

### Relative maximum entropy approach to correct polymer simulations by rescaled QHR-4C data

In statistical mechanics, the 3D conformations of the polymer are microstates, which cannot be observed directly in experiments. In single-cell experiments, some macroscopic variables, such as contact strength between monomers, can be observed for single microstates. In multiple-cell experiments, only the mean of macroscopic variables over an ensemble of microstates can be observed. As an inverse problem, inferring the distribution of microstates from the macroscopic variables can be achieved in two different ways. The first is the maximum entropy approach. One searches for the best in all microstate distributions which coincide with the observed macroscopic variables, and choose the distribution with the maximum entropy. The justification for this is that one should introduce as little information as possible other than that from the direct observation. The second is the model-based simulation. One sets up a computation model, such as the cohesin loop extrusion, and simulates many microstates. The difficulty comes from the parameter choice. Generally, novel methods are used to optimize parameters by minimizing the differences between the macroscopic variables calculated from the simulated microstates and those observed from experiments. Depending on the problem, the optimization process can be extremely hard and achieve very limited improvements.

The advantage of the maximum entropy approach is that the predicted distribution of microstates resulting in the same macroscopic variables as the observations. The disadvantage is that it abandons all known central mechanisms, such as the cohesin loop extrusion. On the contrary, the model-based simulation includes known mechanisms to set up the model but predicts the macroscopic variables usually deviating from the observations. We use the relative maximum entropy approach [83] that combines the advantages of both the maximum entropy approach and the model-based simulations. The basic idea is quite similar to the maximum entropy approach. One searches the best in all microstate distributions which coincide with the observed macroscopic variables. The difference is that the distribution with the maximum entropy relative to that determined by the underlying model, namely the relative entropy instead of the entropy, is chosen. The entropy is actually the relative entropy to the uniform distribution, which is the least informative distribution. If an underlying model is set up based on new information, then the least informative distribution will be determined by the underlying model. The relative entropy, or the negative Kullback-Leibler divergence, is a measurement of the similarity between two distributions. The relative maximum entropy approach actually selects a distribution closest to the least informative one among those satisfying the experimental observations.

We apply the relative maximum entropy approach [83] to correct polymer simulations by the rescaled QHR-4C data. Pairs with 0 contact frequencies in both replicates are excluded. In simulations, long-distance chromatin interactions between the bin of viewpoint and all other bins are determined by their spatial distances in the 3D conformation and the capture radius *ℂ* (the distance at which two monomers are determined to be in contact). Let $k:= \arg \underset{k^{\prime }>0}{\min }{\sum}_{i=1}^I{\sum}_{j=1}^{J_i}{\left({\overline{u}}_{ij}-\frac{\max \left(0,{p}_{ij}-\hat{p}\right)}{k^{\prime }}\right)}^2$ be a multiplier transforming the contact frequencies to the contact probabilities, where ${\overline{u}}_{ij}$ is the mean contact frequencies over the rescaled 4C replicates, *p*_*ij*_ is the contact probabilities of simulations, and $\hat{p}$ is the median contact probabilities over all pairs in the *Pcdh* locus. We subtract $\hat{p}$ to remove the abnormally high background contact probabilities due to the small period box for simulations. Specifically, we initialize the polymer of length *L* = 1266 by a compact conformation [84] (a cubic lattice) in a period box of size ${\left(\frac{L}{\rho}\right)}^{\frac{1}{3}}\approx 18.4984$ with the monomer density *ρ* = 0.2. Even for the relatively short capture radius of 2, the background contact probabilities in such a small box are much higher than those observed in 4C data.

Let *P*_0_(*q*) be the distribution of the 3D conformation *q* of the *Pcdh* locus determined by the underlying loop extrusion model. Let *c*_*ij*_(*q*) = 1 if monomers *i* and *j* are within the capture radius, and *c*_*ij*_(*q*) = 0 otherwise. To prevent overfitting, we assume independent Gaussian errors ${\epsilon}_{ij}\sim \mathcal{N}\left({\epsilon}_{ij};0,{\sigma}_{ij}^2\right)$ with variance ${\sigma}_{ij}^2:= \max \left[{\sigma}_{\mathrm{min}}^2,{\overset{\sim }{\sigma}}_{ij}^2\right]$ for the contact probabilities of (*i*, *j*), where ${\overset{\sim }{\sigma}}_{ij}^2$ is the variance of *ku*_*ij*_ over experimental replicates, and ${\sigma}_{\mathrm{min}}^2$ is the minimally allowed variance. Then the union distribution *Q*_0_(*q*, *ϵ*) is:

$$ {Q}_0\left(q,\epsilon \right)={P}_0(q)\prod \limits_{j=1}^{J_i}\mathcal{N}\left({\epsilon}_{ij};0,{\sigma}_{ij}^2\right). $$

Force *Q*(*q*, *ϵ*) to reproduce the experimentally observed mean contact probabilities, i.e.:

$$ \int \left({c}_{ij}(q)+{\epsilon}_{ij}\right)Q\left(q,\epsilon \right) dqd\epsilon ={\xi}_{ij}:= \min \left(1,k{\overline{u}}_{ij}+\min \left({p}_{ij},\hat{p}\right)\right), $$

while maximizing the relative entropy:

1 $$ \mathcal{S}\left[Q\right]\left[{Q}_0\right]:= -\int Q\left(q,\epsilon \right)\log \left[Q\left(q,\epsilon \right)/{Q}_0\left(q,\epsilon \right)\right] dqd\epsilon . $$

By the variational methods [83]:

2 $$ Q\left(q,\epsilon \right)\propto Q\left(q,\epsilon; \lambda \right):= \exp \left(-\sum \limits_{i=1}^I\sum \limits_{j=1}^{J_i}{\lambda}_{ij}\left[{c}_{ij}(q)+{\epsilon}_{ij}\right]\right){Q}_0\left(q,\epsilon \right), $$

where λ is determined by Eq. (1) and the normalization restraint ∫*Q*(*q*, *ϵ*)*dqdϵ* = 1. This *λ* must minimize [83]:

$$ \Gamma \left(\lambda \right)=\log \left[\int Q\left(q,\epsilon; \lambda \right) dq d\epsilon \right]+\sum \limits_{i=1}^I\sum \limits_{j=1}^{J_i}{\lambda}_{ij}{\epsilon}_{ij}=\log \left[\int \exp \left(-\sum \limits_{i=1}^I\sum \limits_{j=1}^{J_i}{\lambda}_{ij}{c}_{ij}(q)\right){P}_0(q) dq\right]+\sum \limits_{i=1}^I\sum \limits_{j=1}^{J_i}{\lambda}_{ij}{\epsilon}_{ij}+\frac{1}{2}\sum \limits_{i=1}^I\sum \limits_{j=1}^{J_i}{\lambda}_{ij}{\sigma}_{ij}^2. $$

The gradient and Hessian of *Γ*(*λ*) are:

$$ \frac{\mathrm{\partial \Gamma }}{\partial {\lambda}_{ij}}={\epsilon}_{ij}-\left\langle {c}_{ij}(q)\right\rangle +{\lambda}_{ij}{\sigma}_{ij}^2, $$

$$ \frac{\partial^2\Gamma}{\partial \lambda \_\left({i}_1{j}_1\right)\partial {\lambda}_{i_2{j}_2}}=\left\langle {c}_{i_1{j}_1}(q){c}_{i_2{j}_2}(q)\right\rangle -\left\langle {c}_{i_1{j}_1}(q)\right\rangle \left\langle {c}_{i_2{j}_2}(q)\right\rangle +{\updelta}_{{\mathrm{i}}_1{\mathrm{i}}_2}{\delta}_{j_1{j}_2}{\sigma}_{i_1{j}_1}^2, $$

where for arbitrary function *f*(q), define:

$$ \left\langle f(q)\right\rangle := \frac{\int f(q)\exp \left(-{\sum}_{i=1}^I{\sum}_{j=1}^{J_i}{\lambda}_{ij}{c}_{ij}(q)\right){P}_0(q) dq}{\int \exp \left(-{\sum}_{i=1}^I{\sum}_{j=1}^{J_i}{\lambda}_{ij}{c}_{ij}(q)\right){P}_0(q) dq}. $$

Sample conformations *q*_1_, *q*_2_, *q*_3_, ⋯, *q*_*N*_ from the distribution *P*_0_(*q*) determined by the underlying model by simulations. Then:

3 $$ \left\langle f(q)\right\rangle \approx \frac{\sum_{n=1}^Nf\left({q}_n\right)\exp \left(-{\sum}_{i=1}^I{\sum}_{j=1}^{J_i}{\lambda}_{ij}{c}_{ij}\left({q}_n\right)\right)}{\sum_{n=1}^N\exp \left(-{\sum}_{i=1}^I{\sum}_{j=1}^{J_i}{\uplambda}_{ij}{c}_{ij}\left({q}_n\right)\right)}. $$

${\sigma}_{\mathrm{min}}^2>0$ promises the strictly positive definition of Hessian, thereby the optimization is strictly convex. Increasing ${\sigma}_{\mathrm{min}}^2$ not only speeds up the convergence, but also keeps |*λ*_*ij*_| small, thereby avoiding overfitting. However, it also extracts less information from the experiments. Therefore, we set ${\sigma}_{\mathrm{min}}^2=0.01$ and solve *λ* by the trust region algorithm.

### Optimization of processivity, separation, and capture radius

We set both processivity and separation to 100, 200, or 400 [19]. For each pair of processivity and separation, we do the following simulations. First, we anneal the loop extrusion dynamics by 1,000,000 1D simulation time steps. We then anneal the 3D dynamics by 2000 blocks, each of which contains one 1D simulation time step and 1250 3D simulation time steps. Finally, we simulate 50,000 blocks and obtain 50,000 conformations.

The above process is repeated twice to obtain 100,000 conformations for each pair of processivity and separation. We then use the relative maximum entropy approach to calculate $\underset{\lambda }{\min}\varGamma \left(\lambda \right)$ for each pair of processivity and separation and each capture radius of 2, 3, or 4. The pair of processivity 400 and separation 200, which maximizes the average $\underset{\lambda }{\min}\varGamma \left(\lambda \right)$ for capture radius of 2, 3, or 4, is considered as optimal because by Eq. (2):

$$ \mathcal{S}\left[Q\right]\left[{Q}_0\right]=-\int Q\left(q,\epsilon \right)\left\{\log \left[\frac{Q\left(q,\epsilon; \lambda \right)}{Q_0\left(q,\epsilon \right)}\right]-\log \left[\int Q\left({q}^{\prime },{\epsilon}^{\prime };\lambda \right)d{q}^{\prime }d{\epsilon}^{\prime}\right]\right\} dqd\epsilon =\int \sum \limits_{i=1}^I\sum \limits_{j=1}^{J_i}{\lambda}_{ij}\left[{c}_{ij}(q)+{\epsilon}_{ij}\right]Q\left(q,\epsilon \right) dq d\epsilon +\log \left[\int Q\left(q,\epsilon; \lambda \right) dq d\epsilon \right]=\log \left[\int \exp \left(-\sum \limits_{i=1}^I\sum \limits_{j=1}^{J_i}{\lambda}_{ij}{c}_{ij}(q)\right){P}_0(q) dq\right]+\sum \limits_{i=1}^I\sum \limits_{j=1}^{J_i}{\lambda}_{ij}\left(\left\langle {c}_{ij}(q)\right\rangle -{\lambda}_{ij}{\sigma}_{ij}^2\right)+\frac{1}{2}\sum \limits_{i=1}^I\sum \limits_{j=1}^{J_i}{\lambda}_{ij}^2{\sigma}_{ij}^2\approx \log \left[\sum \limits_{n=1}^N\exp \left(-\sum \limits_{i=1}^I\sum \limits_{j=1}^{J_i}{\lambda}_{ij}{c}_{ij}\left({q}_n\right)\right)\right]-\log N+\sum \limits_{i=1}^I\sum \limits_{j=1}^{J_i}{\lambda}_{ij}\left(\left\langle {c}_{ij}(q)\right\rangle -{\lambda}_{ij}{\sigma}_{ij}^2\right)+\frac{1}{2}\sum \limits_{i=1}^I\sum \limits_{j=1}^{J_i}{\lambda}_{ij}^2{\sigma}_{ij}^2. $$

As convergence, $\frac{\mathrm{\partial \Gamma }}{\partial {\lambda}_{ij}}={\xi}_{ij}-\left\langle {c}_{ij}(q)\right\rangle +{\lambda}_{ij}{\sigma}_{ij}^2=0$, thereby $\mathcal{S}\left[Q\right]\left[{Q}_0\right]\approx \Gamma \left(\lambda \right)$. The optimal parameter set is repeated 26 times for each sample to obtain 1,300,000 conformations. $\hat{\lambda}:= \underset{\lambda }{\mathrm{argmin}}\varGamma \left(\lambda \right)$ obtained from the wild-type sample by the relative maximum entropy is then used to weight conformations of the mutant samples by Eq. (3) to obtain final entropy-corrected contact probabilities $\tilde{p}_{ij}$.

### Hulu model

Consider a simple genomic region containing four convergent CBS elements (two forward CBS elements followed by two reverse ones) with spaces of 100 bins and stalling probabilities $\mathcal{T}=0.97$. We assume that cohesins mainly load between the inner convergent CBS pair (30 times faster than other locations), two heads of a cohesin advance in the same speed, and the processivity is large enough. We simulate 210,000 conformations for this model region with processivity 400 and separation 400. The contact map *ℍ* is generated by capture radius 2. To give an intuitive expression of the Hulu structure, we transformed *ℍ* to a distance matrix $\mathbbm{D}$ by ${d}_{ij}={h}_{ij}^{-1}$ and applied the non-metric multidimensional scaling with Kruskal’s normalized stress-1 criterion.

### Genome-wide insulator analyses by Bayesian networks

Bayesian networks are a powerful and widely used probabilistic model to infer the underlying conditional dependency of factors shared by a group of instances. In our case, each instance is a promoter, which has four factors: the enhancer strength, the insulator strength, the loop strength, and the promoter activity. Bayesian networks are a non-cyclic directed graph which uses nodes to represent factors and arrows to connect them. The networks are learned by maximizing the posterior likelihood. An arrow from the insulator strength to the promoter activity means it is a direct dependence. We analyzed 207,663 enhancer-promoter contacts of the capture Hi-C data for the genome-wide relationship between insulators, enhancers, and promoters [46]. For each bait promoter fragment, containing a promoter whose activity is represented by the expression level *f*_*i*_, with starting chromosomal coordinate *s*_*i*_ and terminating coordinate *t*_*i*_, we denote it by [*s*_*i*_, *t*_*i*_]. It forms long-distance chromatin contacts, measured as loop counts *l*_*ij*_ in the capture Hi-C experiments, with a putative enhancer fragment [*s*_*j*_, *t*_*j*_]. The enhancer strength *e*_*j*_ of the fragment [*s*_*j*_, *t*_*j*_] is defined as its total H3K27ac signals from ChIP-seq experiments. The insulator strength *u*_*ij*_ of the loop is defined as the total CTCF ChIP signals in the interval [min(*t*_*i*_, *t*_*j*_) + *a*, max(*s*_*i*_, *s*_*j*_) − *a*] (*a* = 500 bp to exclude the rare cases that promoters or enhancers themselves contain CTCF binding sites) if min(*t*_*i*_, *t*_*j*_) + *a* ≤ max(*s*_*i*_, *s*_*j*_) − *a*, and zero otherwise. Let ${\mathcal{T}}_i$ be the set of enhancer fragments which have chromatin contacts with the bait promoter fragment [*s*_*i*_, *t*_*i*_]. The total enhancer strength for the bait promoter [*s*_*i*_, *t*_*i*_] is defined as ${E}_i:= {\sum}_{j\in {\mathcal{T}}_i}{e}_j$. The mean chromatin looping strength is defined as ${L}_i:= \frac{\sum_{j\in {\mathcal{T}}_i}{e}_j{l}_{ij}}{E_i}$. The mean insulator strength is defined as ${U}_i:= \frac{\sum_{j\in {\mathcal{T}}_i}{e}_j{u}_{ij}}{E_i}$. Finally, we use the ranking of the above variables on day 0, day 3, and day 6 to discrete them. For example, let ${f}_i^0$, ${f}_i^3$, and ${f}_i^6$ be the expression levels of the promoter [*s*_*i*_, *t*_*i*_] in days 0, 3, and 6, respectively, with ${f}_i^3<{f}_i^0<{f}_i^6$. Then, we set ${f}_i^0=2$, ${f}_i^3=1$, and ${f}_i^6=3$ to learn the structure of the Bayesian network by the following method.

Let **X** ≔ {*X*_1_, *X*_2_, ⋯, *X*_*n*_} be the set of *n* discrete random variables. **x** ≔ {*x*_1_, *x*_2_, ⋯, *x*_*n*_} is the specific value of **X**. ${x}_i^k$ for 1 ≤ *k* ≤ *r*_*i*_ are the *r*_*i*_ possible values of *X*_*i*_. Given a network structure *S*, $\mathbf{P}{\mathbf{a}}_i^S\subset \mathbf{X}$ are the parents of *X*_*i*_, and $\mathbf{p}{\mathbf{a}}_i^S\subset \mathbf{x}$ are the corresponding specific value. $\mathbf{p}{\mathbf{a}}_i^{S,j}$ for $1\le j\le {q}_i^S$ are the ${q}_i^S$ possible values of $\mathbf{P}{\mathbf{a}}_i^S$. Define:

$$ {\boldsymbol{\theta}}^S:= {\bigcup}_{i=1}^n{\boldsymbol{\theta}}_i^S:= {\bigcup}_{i=1}^n{\bigcup}_{j=1}^{q_i^S}{\boldsymbol{\theta}}_{ij}^S:= {\bigcup}_{i=1}^n{\bigcup}_{j=1}^{q_i^S}{\bigcup}_{k=1}^{r_i}\left\{{\theta}_{ij k}^S\right\}, $$

$$ {\boldsymbol{\alpha}}^S:= \bigcup \limits_{i=1}^n{\boldsymbol{\alpha}}_i^S:= \bigcup \limits_{i=1}^n\bigcup \limits_{j=1}^{q_i^S}{\boldsymbol{\alpha}}_{ij}^S:= \bigcup \limits_{i=1}^n\bigcup \limits_{j=1}^{q_i^S}\bigcup \limits_{k=1}^{r_i}\left\{{\alpha}_{ij k}^S\right\}, $$

where ${\theta}_{ijk}^S>0$, ${\alpha}_{ijk}^S>0$, ${\sum}_{k=1}^{r_i}{\theta}_{ijk}^S=1$. Introduce the independence assumption.

$$ p\left({\boldsymbol{\theta}}^S|{\boldsymbol{\alpha}}^S,S\right)=\prod \limits_{i=1}^n\prod \limits_{j=1}^{q_i^S}p\left({\boldsymbol{\theta}}_{ij}^S|{\boldsymbol{\alpha}}_{ij}^S,S\right). $$

Assume $p\left({x}_i^k|\mathbf{p}{\mathbf{a}}_i^{S,j},{\boldsymbol{\theta}}_i^S,S\right)={\theta}_{ijk}^S$ and $p\left({\boldsymbol{\theta}}_{ij}^S|{\boldsymbol{\alpha}}_{ij}^S,S\right)=\mathcal{D}\left({\boldsymbol{\theta}}_{ij}^S|{\boldsymbol{\alpha}}_{ij}^S\right)$, where $\mathcal{D}\left({\boldsymbol{\theta}}_{ij}^S|{\boldsymbol{\alpha}}_{ij}^S\right)$ is the Dirichlet distribution of ${\boldsymbol{\theta}}_{ij}^S$ with parameter ${\boldsymbol{\alpha}}_{ij}^S$.

*D* ≔ {*d*_*l*_| 1 ≤ *l* ≤ *m*} are *m* samples. *d*_*li*_ and $\mathbf{p}{\mathbf{a}}_{li}^S$ are the values of variable *i* and its parents in sample *l*, respectively. Define:

$$ {\delta}_{ijk}^{S,l}:= \left\{\begin{array}{c}1,\mathbf{p}{\mathbf{a}}_{li}^S=\mathbf{p}{\mathbf{a}}_i^{S,j},{d}_{li}={x}_i^k,\\ {}0,\mathrm{otherwise},\end{array}\right.\ {N}_{ijk}^{S,l}:= \sum \limits_{l^{\prime }=1}^{l-1}{\delta}_{ijk}^{S,{l}^{\prime }}, $$

$$ {\mathbf{N}}_{ij}^{S,l}=\left\{{N}_{ij k}^{S,l}|1\le k\le {r}_i\right\},{D}_l:= \left\{{d}_{l^{\prime }}|1\le {l}^{\prime }<l\right\}. $$

${N}_{ijk}^{S,l}$ is the number of samples in *D*_*l*_ with variable *i* taking the *k*th value ${x}_i^k$ and its parents taking the *j*th value $\mathbf{p}{\mathbf{a}}_i^{S,j}$. Then, it is well known that $p\left({\boldsymbol{\theta}}_{ij}^S|{D}_l,{\boldsymbol{\alpha}}_{ij}^S,S\right)=\mathcal{D}\left({\boldsymbol{\theta}}_{ij}^S|{\boldsymbol{\alpha}}_{ij}^S+{\mathbf{N}}_{ij}^{S,l}\right)$. Also:

$$ p\left({\boldsymbol{\theta}}_i^S|{D}_l,{\boldsymbol{\alpha}}^S,S\right)=\frac{p\left({D}_l|{\boldsymbol{\theta}}_i^S,S\right)p\left({\boldsymbol{\theta}}_i^S|{\boldsymbol{\alpha}}^S,S\right)}{p\left({D}_l|{\boldsymbol{\alpha}}^S,S\right)}=\frac{\left[{\prod}_{j=1}^{q_i^S}{\prod}_{k=1}^{r_i}{\left({\theta}_{ij k}^S\right)}^{N_{ij k}^{S,l}}\right]\left[{\prod}_{j=1}^{q_i^S}p\left({\boldsymbol{\theta}}_{ij}^S|{\boldsymbol{\alpha}}^S,S\right)\right]}{p\left({D}_l|{\boldsymbol{\alpha}}^S,S\right)}=\frac{\left[{\prod}_{j=1}^{q_i^S}p\left({D}_l|{\boldsymbol{\theta}}_{ij}^S,S\right)\right]\left[{\prod}_{j=1}^{q_i^S}p\left({\boldsymbol{\theta}}_{ij}^S|{\boldsymbol{\alpha}}^S,S\right)\right]}{p\left({D}_l|{\boldsymbol{\alpha}}^S,S\right)}=\frac{\left[{\prod}_{j=1}^{q_i^S}p\left({D}_l,{\boldsymbol{\theta}}_{ij}^S|{\boldsymbol{\alpha}}^S,S\right)\right]}{p\left({D}_l|{\boldsymbol{\alpha}}^S,S\right)}=\prod \limits_{j=1}^{q_i^S}\mathcal{D}\left({\boldsymbol{\theta}}_{ij}^S|{\boldsymbol{\alpha}}_{ij}^S+{\mathbf{N}}_{ij}^{S,l}\right). $$

Thus:

$$ p\left(D|{\boldsymbol{\alpha}}^S,S\right)=\prod \limits_{l=1}^m\prod \limits_{i=1}^n\int d{\boldsymbol{\theta}}_i^Sp\left({d}_{li}|\mathbf{p}{\mathbf{a}}_{li}^S,{\boldsymbol{\theta}}_i^S,S\right)p\left({\boldsymbol{\theta}}_i^S|{D}_l,{\boldsymbol{\alpha}}^S,S\right)=\prod \limits_{l=1}^m\prod \limits_{i=1}^n\int d{\boldsymbol{\theta}}_i^S\left[\prod \limits_{j=1}^{q_i^S}\prod \limits_{k=1}^{r_i}{\left({\theta}_{ij k}^S\right)}^{\delta_{ij k}^{S,\mathrm{l}}}\right]\left[\prod \limits_{j=1}^{q_i^S}\mathcal{D}\left({\boldsymbol{\theta}}_{ij}^S|{\boldsymbol{\alpha}}_{ij}^S+{\mathbf{N}}_{ij}^{S,l}\right)\right]=\prod \limits_{l=1}^m\prod \limits_{i=1}^n\int \left[\prod \limits_{j=1}^{q_i^S}d{\boldsymbol{\theta}}_{ij}^S\right]\left[\prod \limits_{j=1}^{q_i^S}\prod \limits_{k=1}^{r_i}{\left({\theta}_{ij k}^S\right)}^{\delta_{ij k}^{S,l}}\right]\left[\prod \limits_{j=1}^{q_i^S}\frac{\Gamma \left(\left|{\boldsymbol{\alpha}}_{ij}^S+{\mathbf{N}}_{ij}^{S,l}\right|\right)}{\prod_{k=1}^{r_i}\Gamma \left({\alpha}_{ij k}^S+{N}_{ij k}^{S,l}\right)}\prod \limits_{k=1}^{r_i}{\left({\theta}_{ij k}^S\right)}^{\alpha_{ij k}^S+{N}_{ij k}^{S,l}-1}\right]=\prod \limits_{l=1}^m\prod \limits_{i=1}^n\left[\prod \limits_{j=1}^{q_i^S}\frac{\Gamma \left(\left|{\boldsymbol{\alpha}}_{ij}^S+{\mathbf{N}}_{ij}^{S,l}\right|\right)}{\prod_{k=1}^{r_i}\Gamma \left({\alpha}_{ij k}^S+{N}_{ij k}^{S,l}\right)}\right]\prod \limits_{j=1}^{q_i^S}\int d{\boldsymbol{\theta}}_{ij}^S\prod \limits_{k=1}^{r_i}{\left({\theta}_{ij k}^S\right)}^{\delta_{ij k}^{S,1}+{\alpha}_{ij k}^S+{N}_{ij k}^{S,l}-1}=\prod \limits_{l=1}^m\prod \limits_{i=1}^n\prod \limits_{j=1}^{q_i^S}\frac{\Gamma \left(\left|{\boldsymbol{\alpha}}_{ij}^S+{\mathbf{N}}_{ij}^{S,l}\right|\right)}{\Gamma \left(\left|{\boldsymbol{\alpha}}_{ij}^S+{\mathbf{N}}_{ij}^{S,l+1}\right|\right)}\prod \limits_{k=1}^{r_i}\frac{\Gamma \left({\alpha}_{ij k}^S+{N}_{ij k}^{S,l+1}\right)}{\Gamma \left({\alpha}_{ij k}^S+{N}_{ij k}^{S,l}\right)}=\prod \limits_{i=1}^n\prod \limits_{j=1}^{q_i^S}\frac{\Gamma \left(\left|{\boldsymbol{\alpha}}_{ij}^S\right|\right)}{\Gamma \left(\left|{\boldsymbol{\alpha}}_{ij}^S+{\mathbf{N}}_{ij}^S\right|\right)}\prod \limits_{k=1}^{r_i}\frac{\Gamma \left({\alpha}_{ij k}^S+{N}_{ij k}^S\right)}{\Gamma \left({\alpha}_{ij k}^S\right)}, $$

where $$ {N}_{ijk}^S:= {\sum}_{l=1}^m{\delta}_{ijk}^{S,l} $$ and $$ {\mathbf{N}}_{ij}^S=\left\{{N}_{ij k}^S|1\le k\le {r}_i\right\} $$.

The independence assumption is valid by assuming prior modularity, marginal likelihood equivalence, and Dirichlet More importantly, there exists $\boldsymbol{\alpha} := \left\{{\alpha}_{\mathbf{x}}|\mathbf{x}\in \mathcal{X}\right\}$ independent of *S*, such that ${\alpha}_{ijk}^S={\sum}_{\mathbf{x}\in {\mathcal{X}}_{ijk}^S}{\alpha}_{\mathbf{x}}$ ∀*S*, where $\mathcal{X}:= {\bigotimes}_{i=1}^n{\mathcal{X}}_i$, ${\mathcal{X}}_i:= \left\{{x}_i^k|1\le k\le {r}_i\right\}$ and ${\mathcal{X}}_{ijk}^S:= \left\{\mathbf{x}\in \mathcal{X}|{x}_i={x}_i^k,\mathbf{p}{\mathbf{a}}_i^S=\mathbf{p}{\mathbf{a}}_i^{S,j}\right\}$. So, *p*(*D*| ***α***^*S*^, *S*) = *p*(*D*| ***α***, *S*). For simplicity, we assume *α*_**x**_**≡***α* (uniform priors) and ***α*** = 1 (limited prior information). The best structure is defined as *S*^∗^ ≔ argmax_*S*_*p*(*S*| *D*, ***α***) = argmax_*S*_*p*(*D*| ***α***, *S*)*p*(*S*)/*p*(*D*| ***α***). For simplicity, assume that *p*(*S*) is uniformly distributed over all possible structures. To find *S*^∗^, we first transform the data into an all-dimensions tree and then apply the max-min hill-climbing (MMHC) algorithm [45].

### Statistics and reproducibility

All statistical tests used were performed using R 3.5 and Microsoft Excel. All of the statistical tests used are described in the relevant text. *p* values are provided as exact values where possible and otherwise are reported as a range. All of QHR-4C, 5C, and RNA-seq experiments were performed with at least two biological replicates. Single-cell CRISPR CBS insertion clones and their corresponding mutant clones were screened for at least two clones for each genotype.

## Supplementary information

**Additional file 1: Figure S1.** Stochastic and monoallelic expression of the *Pcdhα* genes in single cells. **Figure S2.** A sensitive QHR-4C method for one-to-all capture of chromosome conformations. **Figure S3.** Both forward and reverse CBS elements inserted between the *Pcdhα* cluster and its downstream *HS5-1* enhancer function as insulators. **Figure S4.** Reverse CBS elements inserted between *Pcdh α13* and *αc1* function as an insulator for the upstream genes. **Figure S5.** Reverse-forward CBS pair as an insulator for the *Pcdhα* genes. **Figure S6.** Reverse-forward tandem CBS pairs as an insulator for the *Pcdhα* genes. **Figure S7.** Forward-reverse convergent CTCF sites do not compromise their insulation activity. **Figure S8.** Polymer simulations of the chromatin looping interaction profiles upon CBS insertions or their mutations in the *Pcdh* and *Igh* clusters. **Figure S9.** Tandem CTCF sites ensure stochastic and balanced *Pcdh* gene expression. **Figure S10.** Topology of spatial chromatin contacts between the *Pcdh β* and *γ* clusters and the downstream super-enhancer. **Figure S11.** Topology of spatial chromatin contacts between the *Pcdhγ* clusters and the downstream super-enhancer. **Figure S12.** Genotyping of the mouse lines of various *HS5-1* CBS deletions and inversions. **Figure S13.** Tandem CTCF sites function as insulators for enhancers with no CBS.

**Additional file 2: Table S1.** Oligonucleotides in this study. **Table S2.** CRISPR deletion and inversion mice. **Table S3.** CRISPR single-cell clones. **Table S4.** Mapping statistics of the 5C data. **Table S5.** Pearson correlations between 5C replicates.

**Additional file 3:** Review history.

## Data Availability

High-throughput sequencing files (QHR-4C, RNA-seq, and ChIP-seq) have been deposited into the NCBI Gene Expression Omnibus (GEO) database with the accession number GSE138646 [85]. 5C data are available from the Sequence Read Archive (SRA) under the accession number PRJNA576991 [86]. The codes for 1D lattice and 3D polymer simulations of tandem-arrayed CTCF sites, maximum-likelihood modeling of *Pcdh* stochastic and monoallelic expression, and genome-wide insulator analyses by Bayesian networks are available at GitHub (https://github.com/ljw20180420/balance_codes) [87].
