# Supplementary material for: Tandem CTCF sites function as insulators to balance spatial chromatin contacts and topological enhancer-promoter selection
Source: Genome Biol. 2020 Mar 23;21:75. doi: 10.1186/s13059-020-01984-7 (PMC7087399; doi:10.1186/s13059-020-01984-7)
Supplement: Supplementary file 1 — Additional file 1: Figure S1. Stochastic and monoallelic expression of the Pcdhα genes in single cells. Figure S2. A sensitive QHR-4C method for one-to-all capture of chromosome conformations. Figure S3. Both forward and reverse CBS elements inserted between the Pcdhα cluster and its downstream HS5-1 enhancer function as insulators. Figure S4. Reverse CBS elements inserted between Pcdh α13 and αc1 function as an insulator for the upstream genes. Figure S5. Reverse-forward CBS pair as an insulator for the Pcdhα genes. Figure S6. Reverse-forward tandem CBS pairs as an insulator for the Pcdhα genes. Figure S7. Forward-reverse convergent CTCF sites do not compromise their insulation activity. Figure S8. Polymer simulations of the chromatin looping interaction profiles upon CBS insertions or their mutations in the Pcdh and Igh clusters. Figure S9. Tandem CTCF sites ensure stochastic and balanced Pcdh gene expression. Figure S10. Topology of spatial chromatin contacts between the Pcdh β and γ clusters and the downstream super-enhancer. Figure S11. Topology of spatial chromatin contacts between the Pcdhγ clusters and the downstream super-enhancer. Figure S12. Genotyping of the mouse lines of various HS5-1 CBS deletions and inversions. Figure S13. Tandem CTCF sites function as insulators for enhancers with no CBS. [file 13059_2020_1984_MOESM1_ESM.docx]

**Additional file 1: Figures S1-S13**

**TABLE OF CONTENT**

1. **Figure S1** Stochastic and monoallelic expression of the *Pcdhα* genes in single cells.
2. **Figure S2** A sensitive QHR-4C method for one-to-all capture of chromosome conformations.
3. **Figure S3** Both forward and reverse CBS elements inserted between the *Pcdhα* cluster and its downstream *HS5-1* enhancer function as insulators.
4. **Figure S4** Reverse CBS elements inserted between *Pcdh α13* and *αc1* function as an insulator for the upstream genes.
5. **Figure S5** Reverse-forward CBS pair as an insulator for the *Pcdhα* genes.
6. **Figure S6** Reverse-forward tandem CBS pairs as an insulator for the *Pcdhα* genes.
7. **Figure S7** Forward-reverse convergent CTCF sites do not compromise their insulation activity.
8. **Figure S8** Polymer simulations of the chromatin looping interaction profiles upon CBS insertions or their mutations in the *Pcdh* and *Igh* clusters.
9. **Figure S9** Tandem CTCF sites ensure stochastic and balanced *Pcdh* gene expression.
10. **Figure S10** Topology of spatial chromatin contacts between the *Pcdh β* and *γ* clusters and the downstream super-enhancer.
11. **Figure S11** Topology of spatial chromatin contacts between the *Pcdhγ* clusters and the downstream super-enhancer.
12. **Figure S12** Genotyping of the mouse lines of various *HS5-1* CBS deletions and inversions.
13. **Figure S13** Tandem CTCF sites function as insulators for enhancers with no CBS.

**
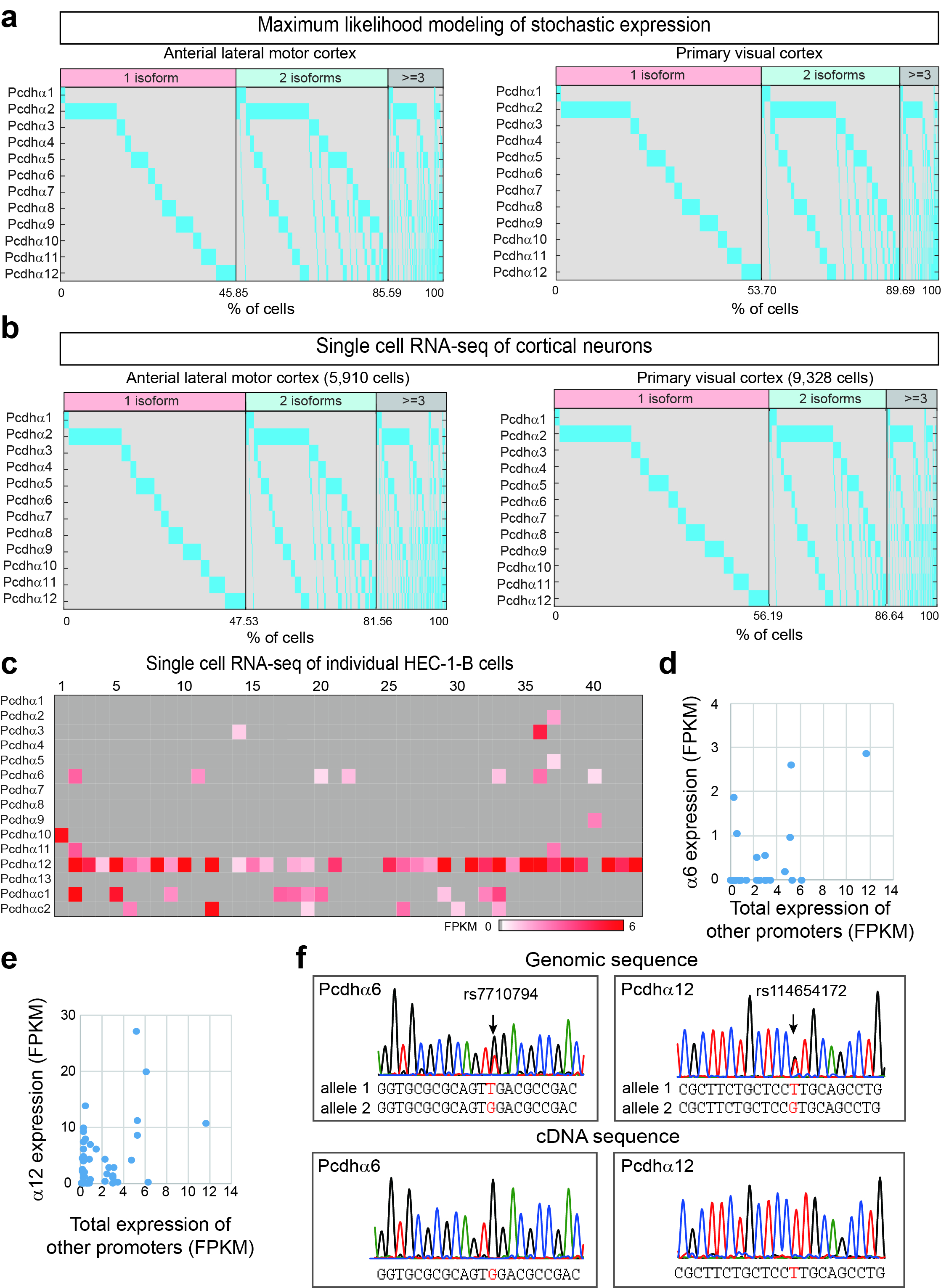
**

**Figure S1** Stochastic and monoallelic expression of the *Pcdhα* genes in single cells. **a** Maximum likelihood modeling of *Pcdhα* stochastic gene expression patterns in single cells. **b** Stochastic expression patterns of the *Pcdhα* genes in single cells from different mouse cortical regions, with most cells express one or two isoforms. Note that the expression patterns are similar between modeling and experimental data. **c** Shown are stochastic expression patterns of the *Pcdhα* genes in individual HEC-1-B cells by single-cell RNA-seq. **d,e** Scatterplots of *Pcdh* *α6* (**d**) and *α12* (**e**) expression versus total expression of other promoters per cell. **f** Shown are Sanger sequencing traces for genomic DNA versus cDNA. Note that two SNPs are detected in *α6* and *α12* variable exons, but only one allele is expressed in single HEC-1-B cells.

**
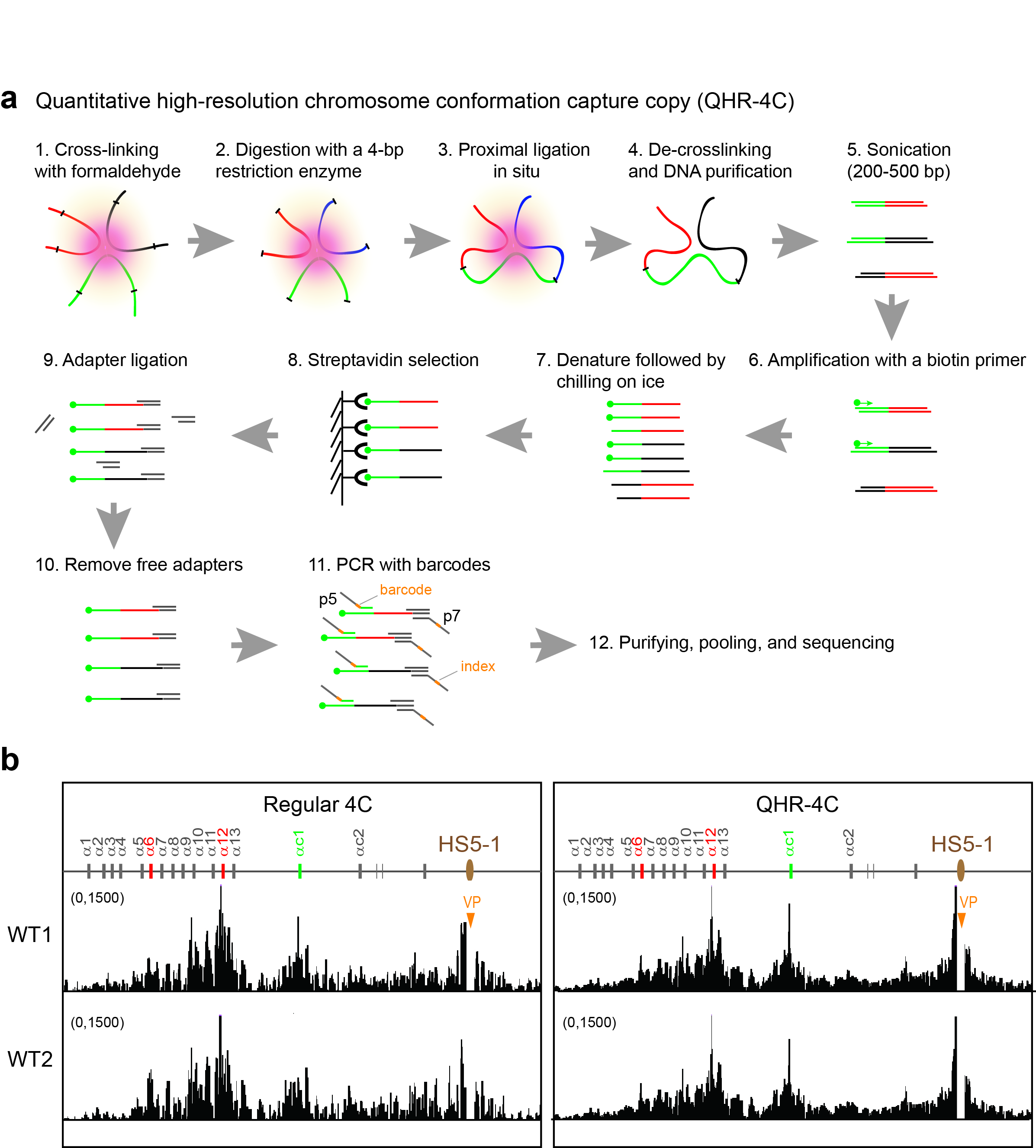
**

**Figure S2** A sensitive QHR-4C method for one-to-all capture of chromosome conformations. **a** Schematic of the QHR-4C method for detecting long-distance chromatin interaction profiles of a viewpoint (see QHR-4C section in Methods for details). **b** Shown are a comparison between the regular-4C (5 million reads) and QHR-4C (half million reads) methods of chromatin interaction profiles. VP: viewpoint.

**
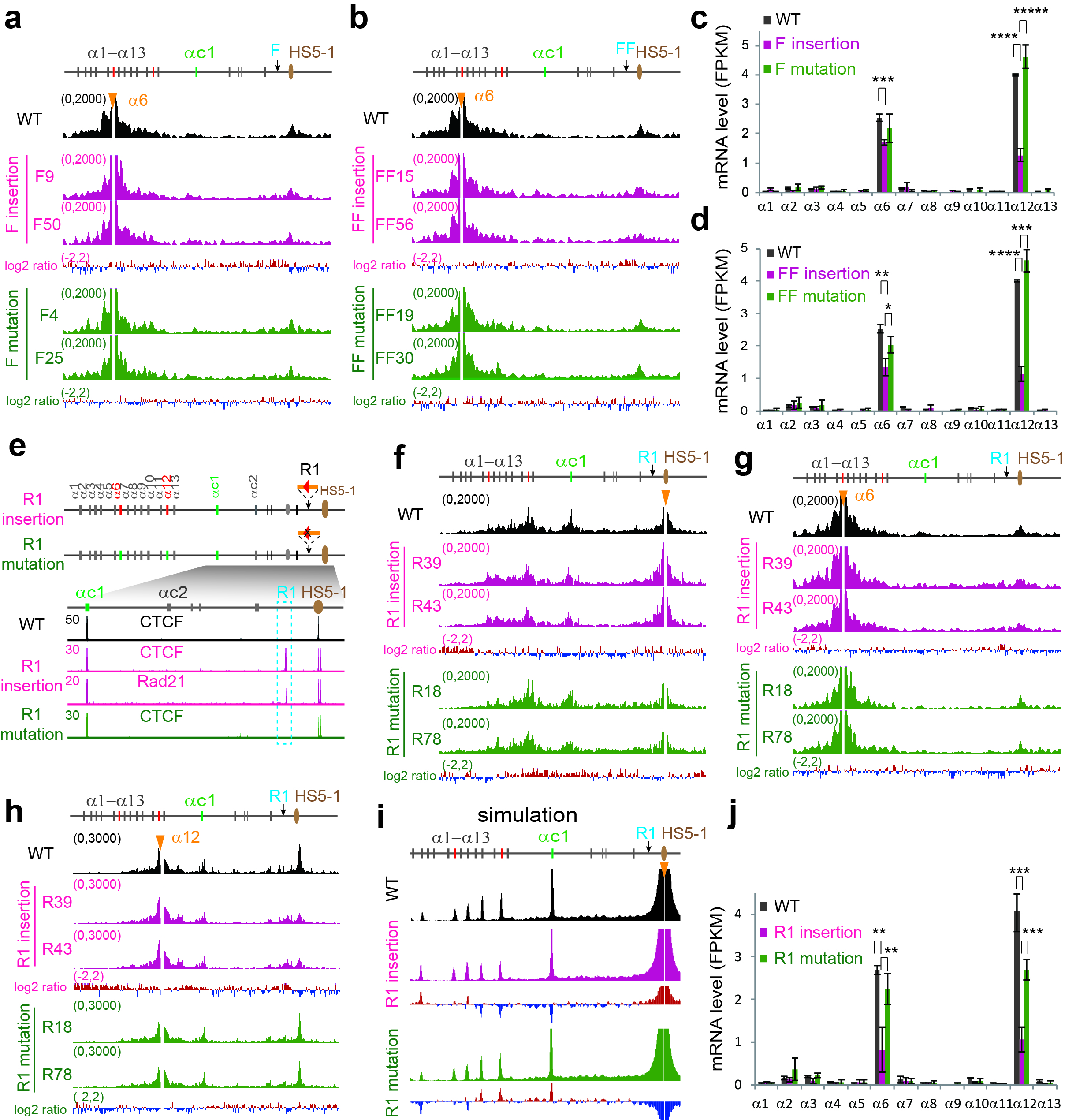
**

**Figure S3** Both forward and reverse CBS elements inserted between the *Pcdhα* cluster and its downstream *HS5-1* enhancer function as insulators. **a,b** 4C profiles with *α6* as a viewpoint in individual CRISPR single-cell clones with insertions of one forward CBS (**a**) (F9,F50) or two forward CBS elements (**b**) (FF15,FF56) and their mutation clones (F4,F25 and FF19,FF30 ). **c,d** Gene expression levels measured by RNA-seq. Data as mean ± SD, **p* < 0.05, ***p* < 0.01, ****p* < 0.001, *****p* < 0.0001, ******p* < 0.00001, one-tailed Student’s *t* test. **e** Schematic showing the insertion of the reverse CBS (“R1”) or its mutation into the location between *Pcdhα* cluster and its downstream *HS5-1* enhancer. CTCF and Rad21 ChIP-seq confirmed their binding to the inserted CBS. For clarity, only regions around the insertions are shown. **f** QHR-4C profiles with *HS5-1* as a viewpoint for CRISPR single-cell clones (WT clones: R39,R43; mutation clones: R18,R78) with the insertion of wildtype CBS or its mutation. **g** QHR-4C profiles with the *α6* promoter as a viewpoint. **h** 4C profiles with the *α12* promoter as a viewpoint. Log2 ratios (insertion vs wildtype or mutation vs insertion) are shown under the 4C profiles. **i** Simulation of chromatin interaction profiles with *HS5-1* as a viewpoint. **j** Gene expression patterns in CRISPR single-cell clones as measured by RNA-seq. Data as mean ± SD, ***p* < 0.01, ****p* < 0.001; one-tailed Student’s *t* test.

**
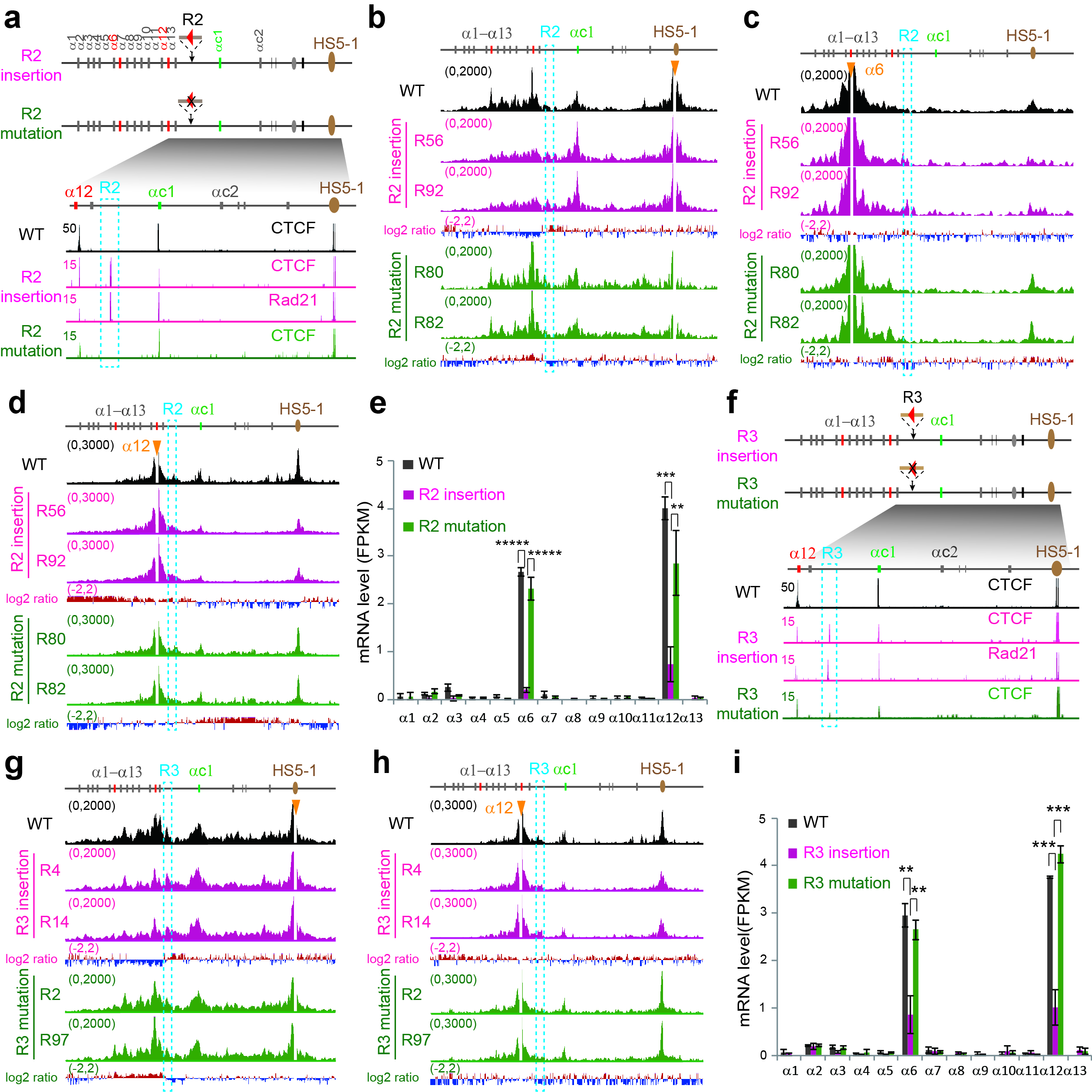
**

**Figure S4** Reverse CBS elements inserted between *Pcdh α13* and *αc1* function as an insulator for the upstream genes. **a** Schematic showing the insertion of the reverse CBS (“R2”) into the location between *α13* and *αc1* within the *Pcdhα* cluster. CTCF and Rad21 ChIP-seq are shown below. **b** 4C profiles with *HS5-1* as a viewpoint. **c** 4C profiles with the *α6* promoter as a viewpoint. **d** 4C profiles with the *α12* promoter as a viewpoint. **e** RNA-seq of the CRISPR single-cell clones. **f** Schematic showing the insertion of the reverse CBS (“R3”) into the location between *α13* and *αc1* within the *Pcdhα* cluster. CTCF and Rad21 ChIP-seq are shown below. **g** 4C profiles with *HS5-1* as a viewpoint. **h** 4C profiles with the *α12* promoter as a viewpoint. **i** Gene expression levels measured by RNA-seq. Data as mean ± SD, ***p* < 0.01, ****p* < 0.001, ******p* < 0.00001; one-tailed Student’s *t* test.

**
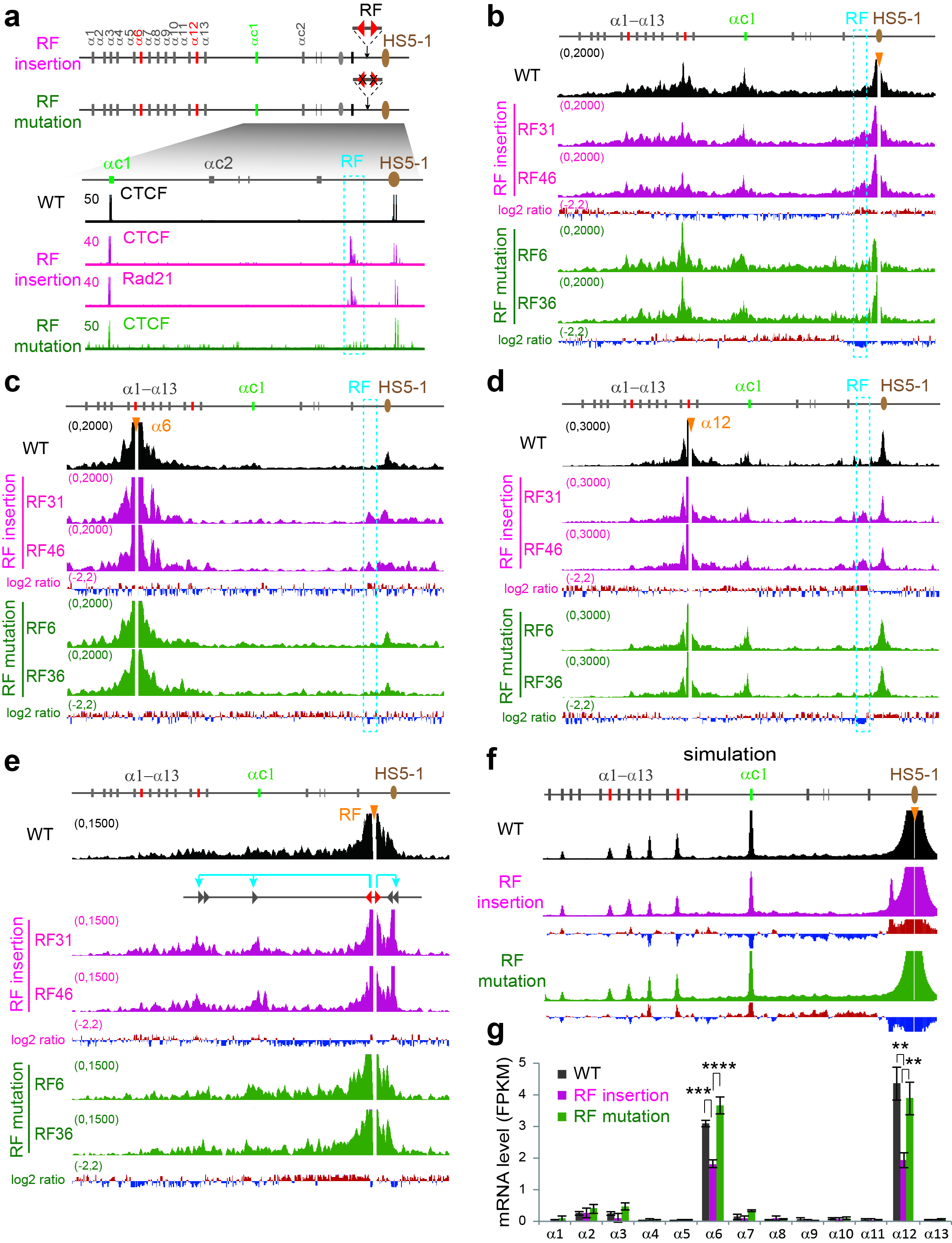
**

**Figure S5** Reverse-forward CBS pair as an insulator for the *Pcdhα* genes. **a** Schematic showing the insertion of reverse-forward CBS elements (“RF”) into the location between the *Pcdhα* cluster and its downstream *HS5-1* enhancer. CTCF and Rad21 ChIP-seq are shown below. **b** QHR-4C profiles with *HS5-1* as a viewpoint for single-cell CRISPR clones of wildtype (RF31,RF46) and its mutation (RF6,RF36). **c** QHR-4C profiles with the *α6* promoter as a viewpoint. **d** QHR-4C profiles with the *α12* promoter as a viewpoint. **e** QHR-4C profiles with the inserted CBS elements as a viewpoint. **f** Simulation of chromatin interaction profiles with *HS5-1* as a viewpoint. **g** RNA-seq of CRISPR insertion clones. WT: wildtype. Data as mean ± SD, ***p* < 0.01, ****p* < 0.001, *****p* < 0.0001; one-tailed Student’s *t* test.

**
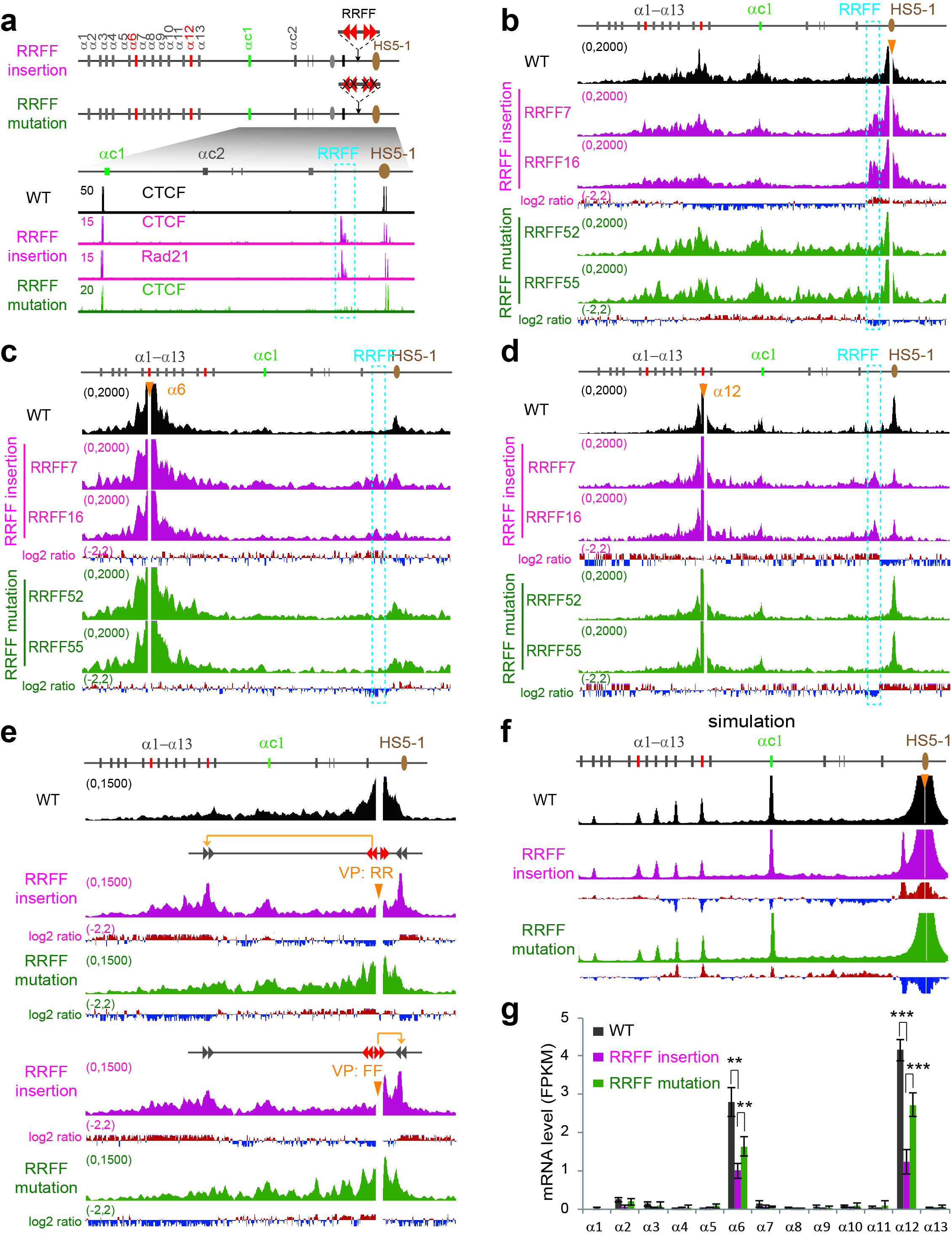
**

**Figure S6** Reverse-forward tandem CBS pairs as an insulator for the *Pcdhα* genes. **a** Shown are the insertions of reverse-forward CBS elements (“RRFF”) into the location between the *Pcdhα* cluster and its downstream *HS5-1* enhancer. CTCF and Rad21 ChIP-seq are shown below. **b** QHR-4C profiles with *HS5-1* as a viewpoint for single-cell CRISPR clones of wildtype (RRFF7,RRFF16) and its mutation (RRFF52,RRFF55). **c** QHR-4C profiles with the *α6* promoter as a viewpoint. **d** QHR-4C profiles with the *α12* promoter as a viewpoint. **e** QHR-4C profiles with the inserted region as a viewpoint. **f** Simulation of chromatin interaction profiles with *HS5-1* as a viewpoint. **g** RNA-seq of CRISPR insertion clones. Data as mean ± SD, ***p* < 0.01, ****p* < 0.001; one-tailed Student’s *t* test.

**
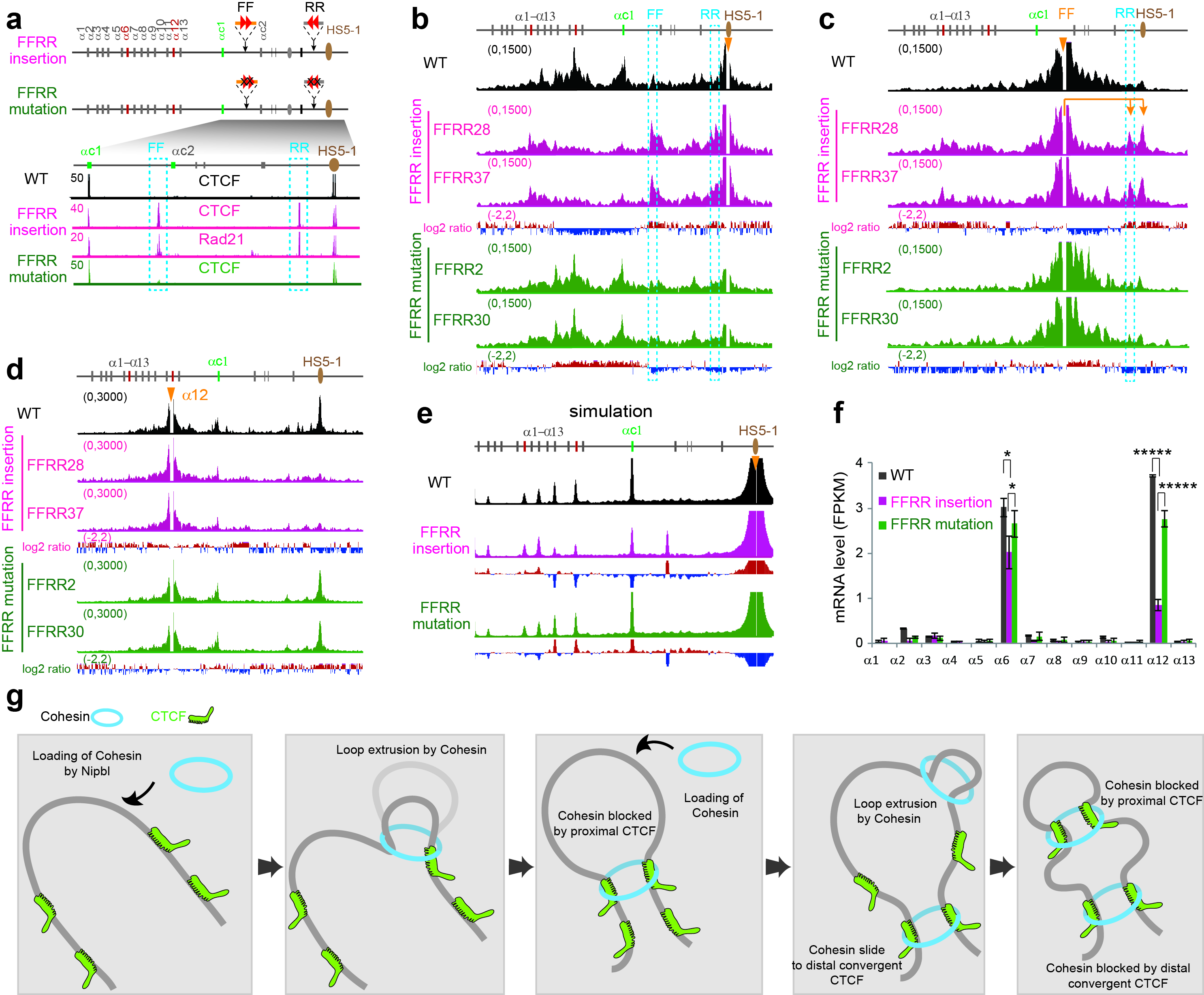
**

**Figure S7** Forward-reverse convergent CTCF sites do not compromise their insulation activity. **a** Schematic showing the insertions of forward (“FF”) and reverse (“RR”) CBS elements into the *Pcdhα* cluster. CTCF and Rad21 ChIP-seq are shown below. **b** QHR-4C profiles with *HS5-1* as a viewpoint for single-cell CRISPR clones of wildtype (FFRR28, FFRR37) and its mutation (FFRR2, FFRR30). **c** QHR-4C profiles with the inserted forward CBS elements as a viewpoint in these CRISPR single-cell clones. **d** QHR-4C profiles with the *α12* promoter as a viewpoint. **e** Simulation of chromatin interaction profiles with *HS5-1* as a viewpoint. **f** RNA-seq of CRISPR insertion clones. Data as mean ± SD, **p* < 0.05, ******p* < 0.00001; one-tailed Student’s *t* test. **g** A “two-headed” extrusion model for cohesin-mediated loop formation. We proposed that, upon non-topological loading, the cohesin ring reels chromatin fibers until reaching CTCF-bound forward-reverse convergent CBS elements which have certain permeability. The active cohesin extrusion process needs energy and depends on ATP hydrolyses. Extrusion is continuous until blocked by CTCF-bound CBS elements which anchor the two cohesin “heads” in an orientation-dependent but permeable manner.


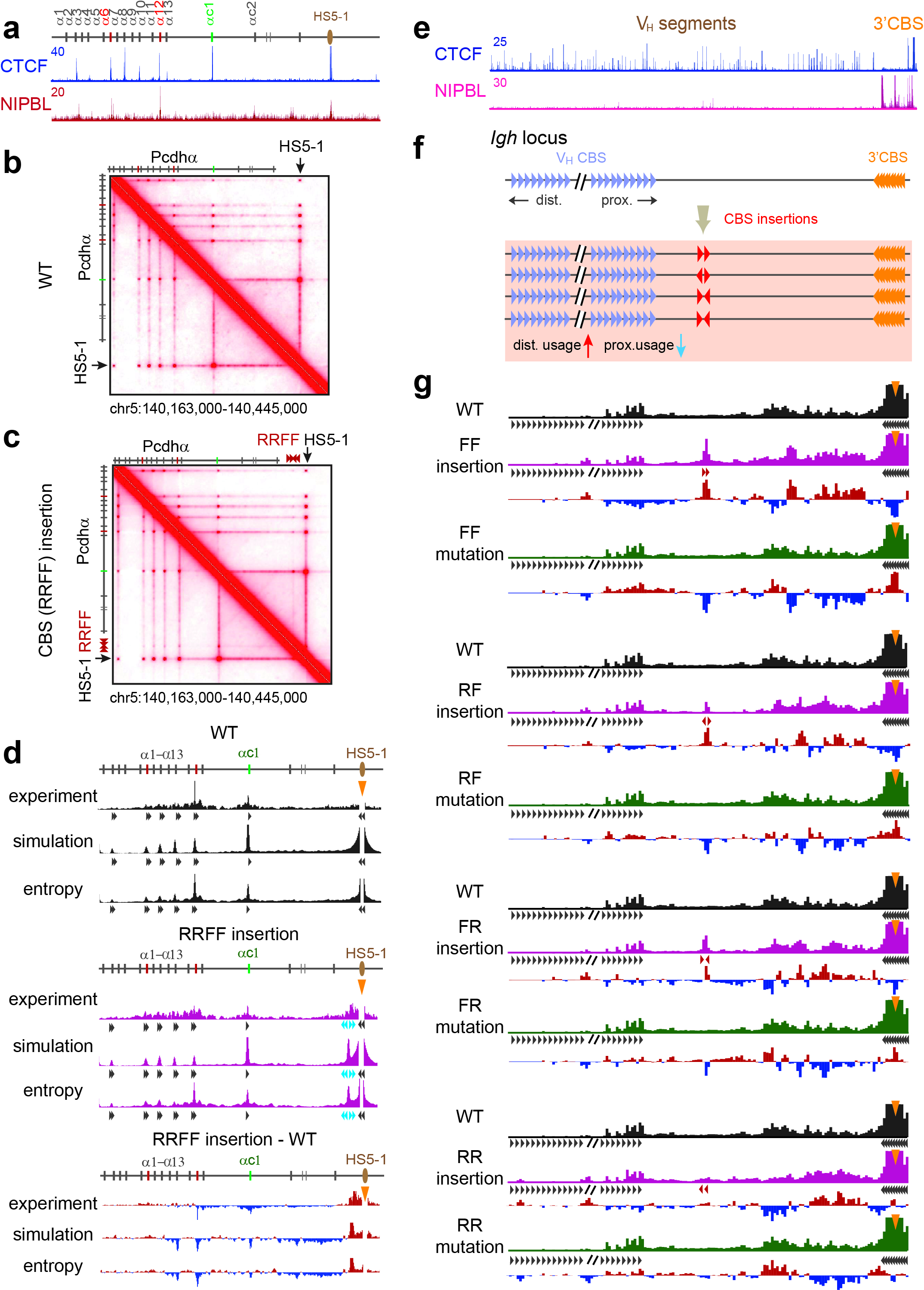


**Figure S8** Polymer simulations of the chromatin looping interaction profiles upon CBS insertions or their mutations in the *Pcdh* and *Igh* clusters. **a** ChIP-seq of CTCF and NIPBL at the *Pcdhα* cluster in HEC-1-B cells. **b,c** Contact maps of ensembles of 1.3 million chromosome conformations of simulated wildtype and CBS insertional *Pcdhα* clones. **d** Improvement of polymer simulation by relative maximum entropy. **e** ChIP-seq of CTCF and NIPBL at the *Igh* cluster. **f** Schematics of increased distal and decreased proximal variable gene segment (*V_H_*) usage upon insertion of various CBS elements in different orientations in the *Igh* cluster. **g** Computational simulation of chromatin interaction profiles with 3’CBS as a viewpoint with insertion of various CBS elements in different orientations and their corresponding mutations revealed similar CBS-dependent augmentation of distal promoter usage in the *Igh* cluster.

**
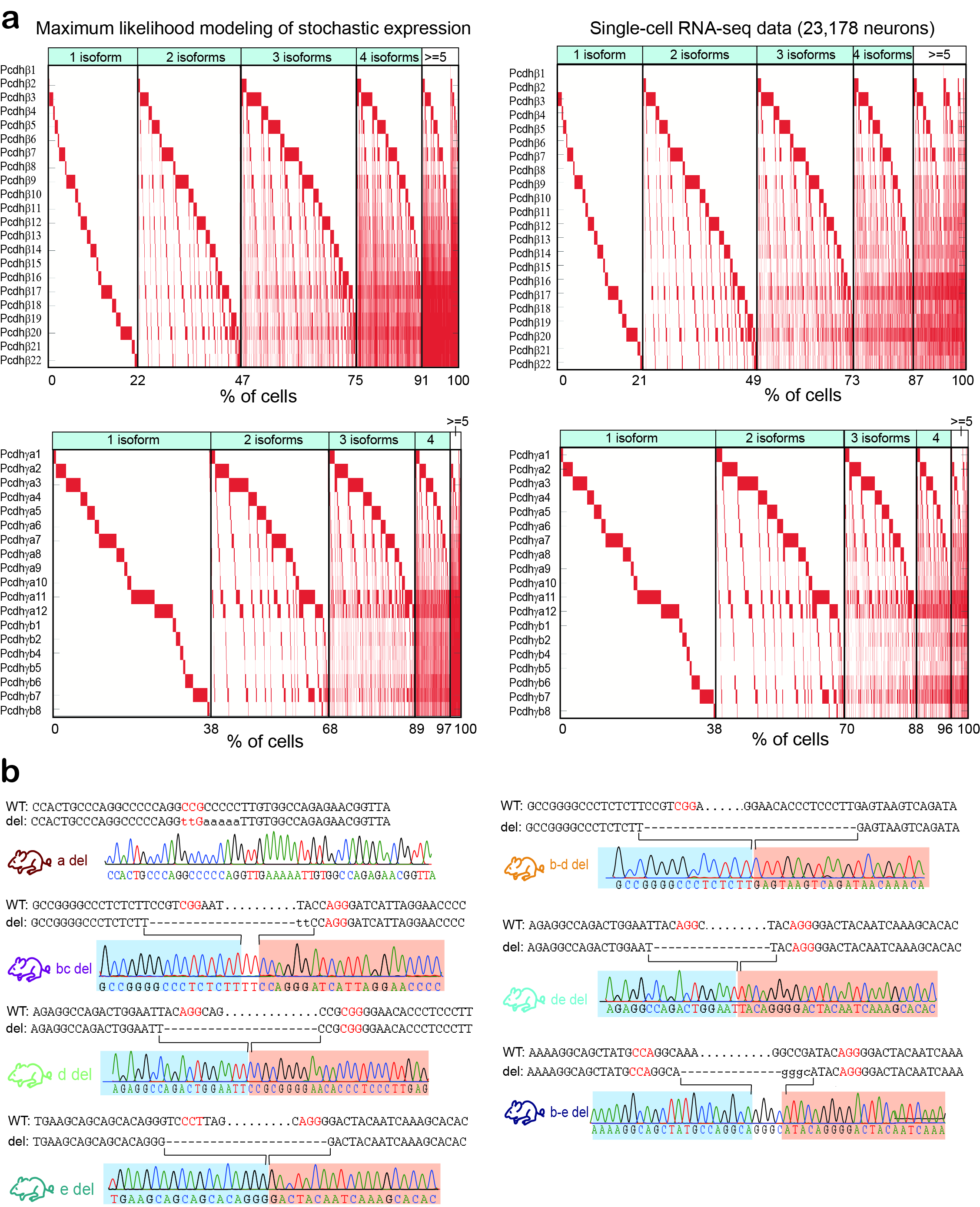
**

**Figure S9** Tandem CTCF sites ensure stochastic and balanced *Pcdh* gene expression. **a** Maximum likelihood modeling and single-cell RNA-seq showed stochastic expression of members of the *Pcdhβ/γ* clusters. Note that vast majority of single neurons express up to 4 *Pcdhβ* isoforms and 4 *Pcdhγ* isoforms. **b** Genotyping of the CBS deletion mouse lines generated by CRISPR DNA-fragment editing. The PAM sites are highlighted.

**
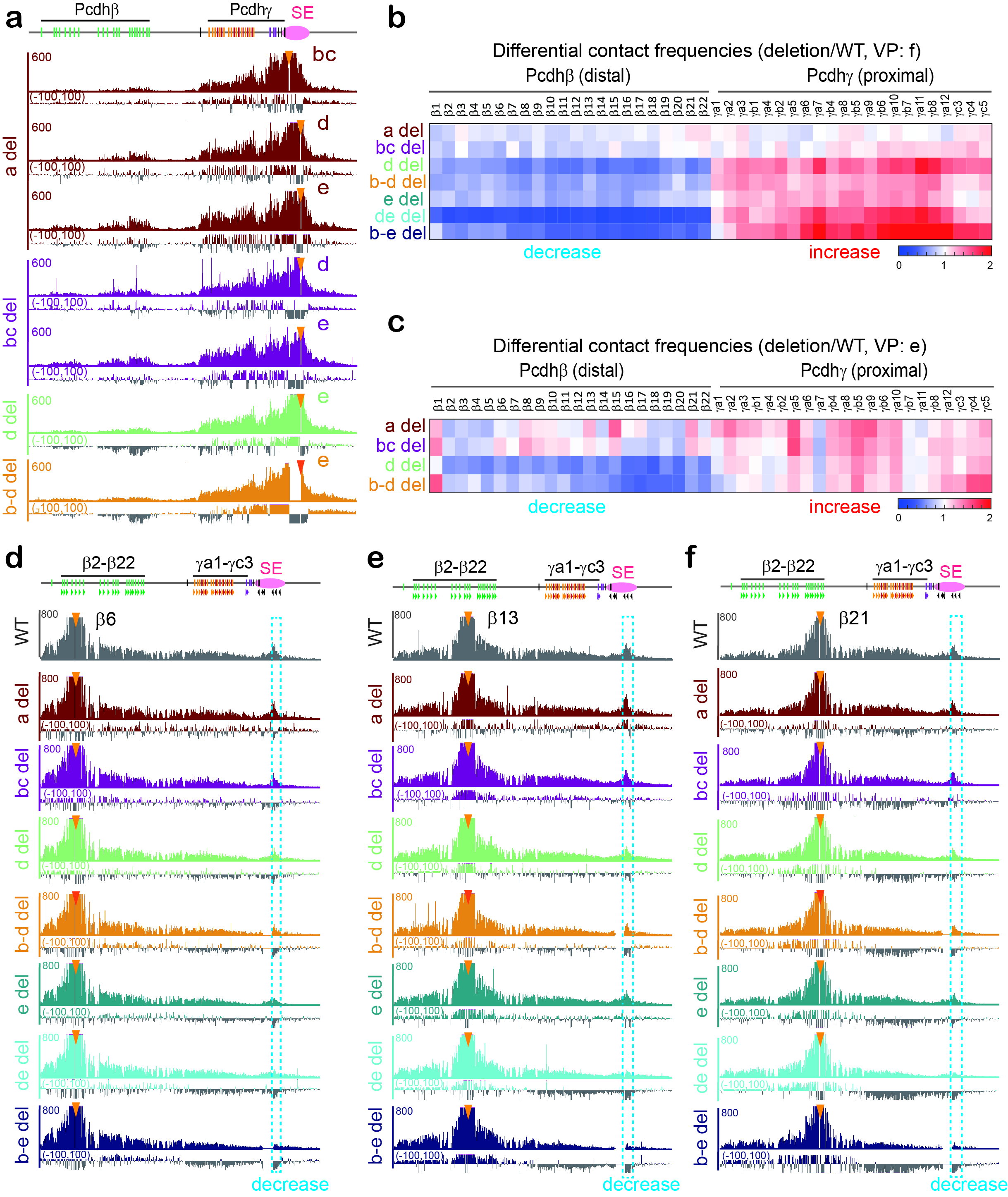
**

**Figure S10** Topology of spatial chromatin contacts between the *Pcdh* *β* and *γ* clusters and the downstream super-enhancer. **a** QHR-4C interaction profiles with a repertoire of the downstream CBS elements as a viewpoint in cortical tissues of mice with various deletions. **b,c** Quantification of differential contact frequencies of the QHR-4C profiles in deletion mice reveals decreased interactions with *Pcdhβ* and increased interactions with *Pcdhγ.* **d-f** QHR-4C interaction profiles with *β6*, *β13*, or *β21* as a viewpoint confirm decreased interactions with the super-enhancer.

**
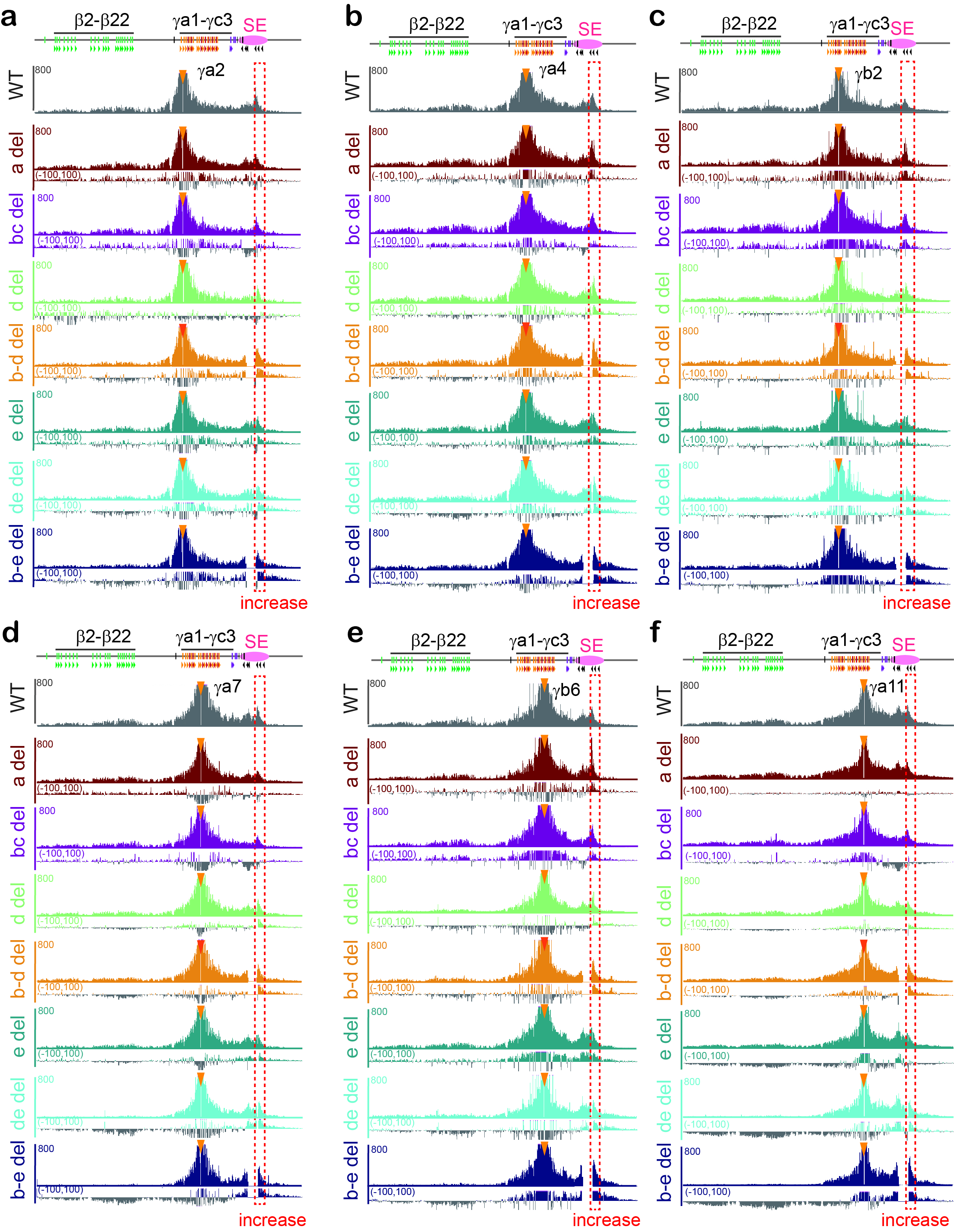
**

**Figure S11** Topology of spatial chromatin contacts between the *γ* clusters and the downstream super-enhancer. QHR-4C interaction profiles with a repertoire of promoter CBS elements of *γa2* (**a**), *γa4* (**b**), *γb2* (**c**), *γa7* (**d**), *γb6* (**e**), and *γa11* (**f**) as a viewpoint show increased interactions with the downstream super-enhancer.


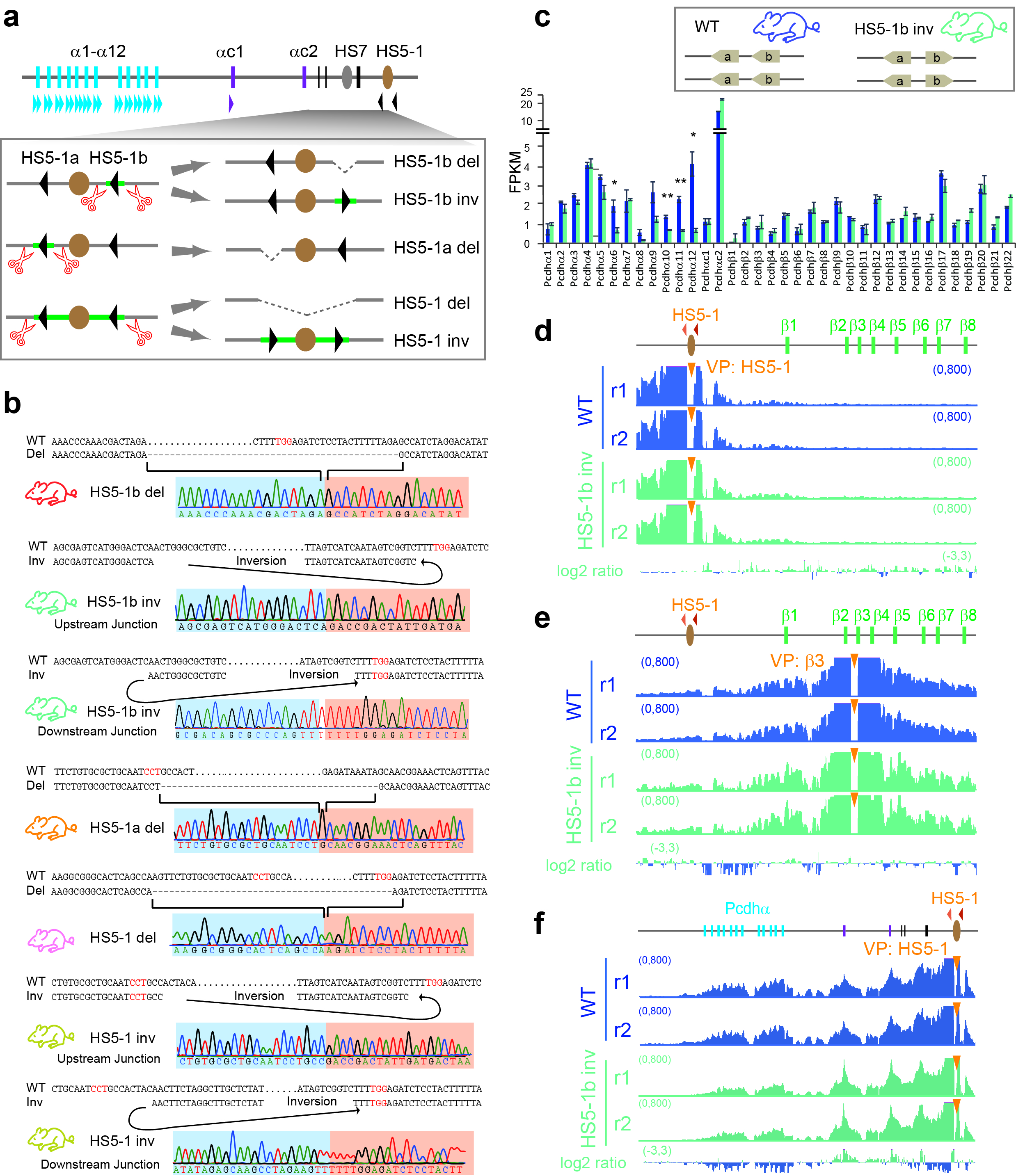


**Figure S12** Genotyping of the mouse lines of various *HS5-1* CBS deletions and inversions. **a** Schematics of the deletion and inversion mouse lines generated by CRISPR DNA-fragment editing. **b** Genotyping of CBS deletion or inversion mouse lines. The PAM sites are highlighted. **c** RNA-seq experiments of cortical tissues of mice with the CBS *HS5-1b* inversion. **d,e** QHR-4C showed no significant alteration of chromatin interactions between *HS5-1* and 5’ isoforms of the *Pcdhβ* cluster upon the CBS *HS5-1b* inversion. **f** QHR-4C experiments showed significant decrease of long-distance chromatin interactions *HS5-1* and the *Pcdhα* cluster. r1, r2: biological replicates 1 and 2.

**
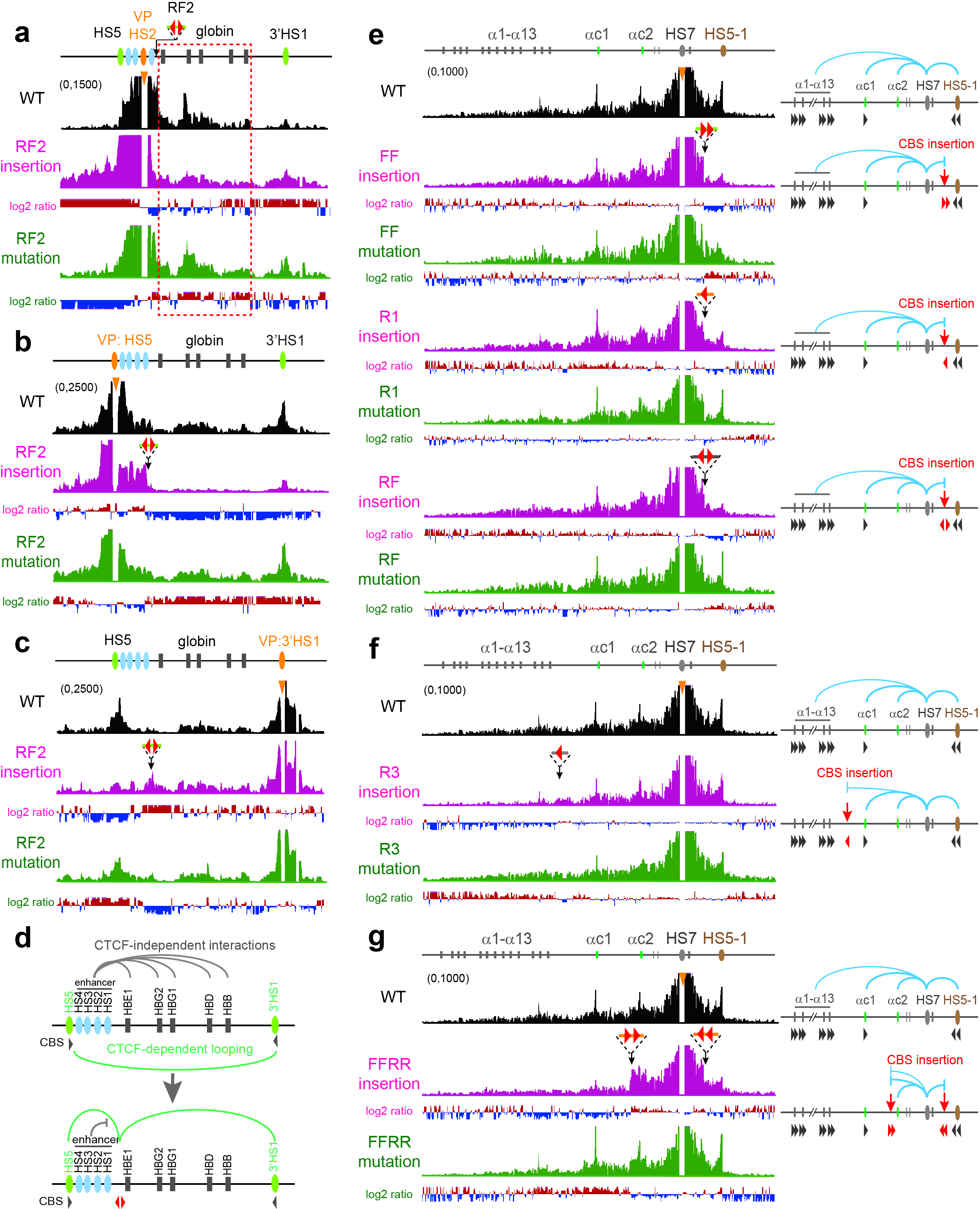
**

**Figure S13** Tandem CTCF sites function as insulators for enhancers with no CBS. **a-c** QHR-4C profiles with (**a**) enhancer *HS2*, (**b**) CBS *5’HS5*, or (**c**) CBS *3’HS1* as a viewpoint upon insertion of a divergent reverse-forward CBS pair in the *β-globin* cluster. **d** Schematic of the chromatin interactions upon insertion of a divergent reverse-forward CBS pair in the *β-globin* cluster. **e** QHR-4C profiles with *HS7* as a viewpoint revealed a significant decrease of chromatin looping interactions with *HS5-1* upon the insertion of tandem-forward, single-reverse, or a pair of reverse-forward CBS elements. Note a sharp decrease of interactions as shown by log2 ratio at the exact insertion site (probably because the bound CTCF blocks cohesin sliding at the insertion site) and the rescue upon the CBS mutations. **f** QHR-4C profiles with *HS7* as a viewpoint revealed a significant decrease of chromatin looping interactions with the alternate *Pcdhα* genes upon the insertion of a reverse CBS into the location between *Pcdh* *α13* and *αc1*. Note that CBS mutation rescues the blocking effects. **g** QHR-4C profiles with *HS7* as a viewpoint revealed a significant decrease of chromatin looping interactions beyond both upstream and downstream insertion sites. Note that CBS mutations rescue the blocking effect.
