# Supplementary material for: Tandem CTCF sites function as insulators to balance spatial chromatin contacts and topological enhancer-promoter selection
Source: Genome Biol. 2020 Mar 23;21:75. doi: 10.1186/s13059-020-01984-7 (PMC7087399; doi:10.1186/s13059-020-01984-7)
Supplement: Supplementary file 2 — Additional file 2: Table S1. Oligonucleotides in this study. Table S2. CRISPR deletion and inversion mice. Table S3. CRISPR single-cell clones. Table S4. Mapping statistics of the 5C data. Table S5. Pearson correlations between 5C replicates. [file 13059_2020_1984_MOESM2_ESM.docx]

**Additional file 2: Tables S1-S5**

**TABLE OF CONTENT**

1. **Table S1.** Oligonucleotides in this study.
2. **Table S2.** CRISPR deletion and inversion mice.
3. **Table S3.** CRISPR single-cell clones.
4. **Table S4.** Mapping statistics of the 5C data.
5. **Table S5.** Pearson correlations between 5C replicates.

**Table S1.** Oligonucleotides in this study.

Oligonucleotides for sgRNA constructs for transfection

| Name | Sequence | | |
| --- | --- | --- | --- |
| Pcdha-sgRNA1-F | accggttcctgctcctgaaacggc | | |
| Pcdha-sgRNA1-R | aaacgccgtttcaggagcaggaac | | |
| Pcdha-sgRNA2-F | accggaataggtatttctccgaag | | |
| Pcdha-sgRNA2-R | aaaccttcggagaaatacctattc | | |
| Pcdha-sgRNA3-F | accggggcgaggtgagcccacttt | | |
| Pcdha-sgRNA3-R | aaacaaagtgggctcacctcgccc | | |
| globin-sgRNA1-F | accgtttaggcacagtggctcccg | | |
| globin-sgRNA1-R | aaaccgggagccactgtgcctaaa | | |
| globin-sgRNA2-F | accgctcagttctagcactagagg | | |
| globin-sgRNA2-R | aaaccctctagtgctagaactgag | | |
| a12-CBSdel-sgRNA1-F | accggctaaaccgaaaagaacctc | | |
| a12-CBSdel-sgRNA1-R | aaacgaggttcttttcggtttagc | | |
| a12-CBSdel-sgRNA2-F | accgattccaatcattcacggaat | | |
| a12-CBSdel-sgRNA2-R | aaacattccgtgaatgattggaat | | |
| ac1-CBSdel-sgRNA1-F | accgagaccccagcccgctgctac | | |
| ac1-CBSdel-sgRNA1-R | aaacgtagcagcgggctggggtct | | |
| ac1-CBSdel-sgRNA2-F | accgagcctccagcctagcaagcc | | |
| ac1-CBSdel-sgRNA2-R | aaacggcttgctaggctggaggct | | |
|  |  | | |
| Oligonucleotides for sgRNA constructs for dCas9 experiments | | | |
| Name | Sequence | | |
| ma-sgRNA-F | accggcccccaggccgcccccttg | | |
| ma-sgRNA-R | aaaccaagggggcggcctgggggc | | |
| mb-sgRNA-F | accgattcagacagcagatggcgc | | |
| mb-sgRNA-R | aaacgcgccatctgctgtctgaat | | |
| mc-sgRNA-F | accgccactacgccacctgccggc | | |
| mc-sgRNA-R | aaacgccggcaggtggcgtagtgg | | |
| md-sgRNA-F | accgtccctgtgttagacaccagg | | |
| md-sgRNA-R | aaaccctggtgtctaacacaggga | | |
| me-sgRNA-F | accggctcaactgcgcacacaccg | | |
| me-sgRNA-R | aaaccggtgtgtgcgcagttgagc | | |
| gal4-sgRNA-F | accgaacgactagttaggcgtgta | | |
| gal4-sgRNA-R | aaactacacgcctaactagtcgtt | | |
|  |  | | |
| Oligonucleotides for CRISPR sgRNAs for microinjection | | | |
| Name | Sequence | | |
| CR-T7-mHS5-1-gRNA1-F | taatacgactcactataggagcctagaagttgtagtggcgttttagagctagaaatag | | |
| CR-T7-mHS5-1-gRNA2-F | taatacgactcactatagggtcatcaatagtcggtctttgttttagagctagaaatag | | |
| CR-T7-mHS5-1a-gRNA-F | taatacgactcactatagggcagctgtctcgccctctgcgttttagagctagaaatag | | |
| CR-T7-mHS5-1b-gRNA-F | taatacgactcactataggatcgaacaccaggtggcagagttttagagctagaaatag | | |
| M-A-sgRNA-F1 | taatacgactcactatagggttctctggccacaaggggggttttagagctagaaatag | | |
| M-BC-sgRNA-F1 | taatacgactcactatagggccggggccctctcttccgtgttttagagctagaaatag | | |
| M-BC-sgRNA-F2 | taatacgactcactatagggtgaaaccacgtaaacctaccgttttagagctagaaatag | | |
| M-D-sgRNA-F1 | taatacgactcactatagggagaggccagactggaattacgttttagagctagaaatag | | |
| M-D-sgRNA-F2 | taatacgactcactatagggaggcatacgcttctattccggttttagagctagaaatag | | |
| M-E-sgRNA-F1 | taatacgactcactatagggacggcagcgtgtcctctagttttagagctagaaatag | | |
| M-E-sgRNA-F2 | taatacgactcactatagggctaagagaggccgatacgttttagagctagaaatag | | |
| M-BCDE-sgRNA-F1 | taatacgactcactatagggacagacatagtcgctttgccgttttagagctagaaatag | | |
| M-BCDE-sgRNA-F2 | taatacgactcactataggggggctaagagaggccgatacgttttagagctagaaatag | | |
| gRNA-R3 | aaaagcaccgactcggtgcc | | |
|  |  | | |
| Oligonucleotides used for constructing the donor plasmids | | | |
| Name | Sequence | | |
| hHS51-donor-F1 | ttaaagtgttttctccattcctgg | | |
| hHS51-donor-R1 | gttgtgaccatgtgagcatgtaat | | |
| hHS51-donor-F2 | ttatgaacctatgctccatgtact | | |
| hHS51-donor-R2 | gtcttcttttggccagtgagaaat | | |
| hHS51-donor-F3 | tgtggaggtagctttactgtgac | | |
| hHS51-donor-R3 | agatgccaaattcaaagtgggac | | |
| hHS51-donor-F4 | taaggtttcccagaattcccacc | | |
| hHS51-donor-R4 | cctaaagtcaattctcctgatgcc | | |
| mRF2-F2 | gaatcttccttgcatttcccccac | | |
| mRF2-R2 | gtgcaggggaaaactctgtagg | | |
| mRF2-F1 | cttgcatttcccccacagtaatgat | | |
| mRF2-R1 | cagacacgtgagcaaggtctac | | |
| mHS51-CBS-F1 | tattgctctgccctgcgttc | | |
| mHS51-CBS-R1 | gttcaaagcgacttgaggtttcc | | |
| mHS51-CBS-F2 | tactgagcagccctgtatcc | | |
| mHS51-CBS-R2 | taggttattttcttgggtggtagtg | | |
| mCTCFbc-F1 | aaatgcatggctgctgtgtg | | |
| mCTCFbe-R1 | ggccctctgtttcctgtctc | | |
| mCTCFbc-F2 | tctgcatcaacgactgtggg | | |
| mCTCFbe-R2 | ggcccataagcctctgatagt | | |
| mHs51-Ctcfbc-R5 | ccaagaaaataacctattgctcccagaggcc | | |
| mCTCFbc-F3 | tgttactactgcttccagtg | | |
| mRF2-R3 | tgaaacgagtcacagctctgggga | | |
| mRF2-F3 | cagcctcctggtttagtccctg | | |
| Puc-hHS51-F2 | aaaacgacggccagtgaattcttatgaacctatgctccatg | | |
| Puc-hHS51-R4 | gaccatgattacgccaagcttcctaaagtcaattctcctgat | | |
| mRF2-S-F1 | gcattggttgtgatttgc | | |
| mRF2-S-F2 | tttcttccaggtttgcgg | | |
| mRF2-S-F3 | atctatatcccaaccgcat | | |
| mHS51-S-F1 | ttgtaaccaccactactctg | | |
| mHS51-S-F2 | caaagtcccagcactgagaa | | |
| mHS51-S-F4 | aggcagcaagtacttcatc | | |
| mRF2-R4 | tattgtttttcctcctatag | | |
| hHS51-donor-R5 | ggctggatctagcagccatattg | | |
| RF-hHS51-F1 | gctgctagatccagcccgctgtccgcatctgg | | |
| RF-R1 | ggaagtggtttttttgagagagactattgatgactaaaac | | |
| RF-hHS51-R1 | ttcctgctcctgaaacggtgtcgctgtccgtg | | |
| hHS51-donor-F5 | gtttcaggagcaggaacaagag | | |
| RF-F1 | tctcaaaaaaaccacttcctgacg | | |
| RF2-mut-R | ctcttcctctccacaatctttcagaattcgggtgtca | | |
| RF2-mut-F | atgctcatagccacaatttttaagcagagacttattc | | |
| Hglobin-donor-F5 | aggccactgaatttctactcca | | |
| hglobin-donor-R5 | agctgagctgctttttgtct | | |
| mRF2-J-R1 | acccacccattccataagaac | | |
| mRF2-J-F1 | tcttaccttaaccagacagggg | | |
| mHS51CTCF-J-R1 | attgcagcgcacagaacttg | | |
| mHS51CTCF-J-F1 | tggcagttacgaaccctagtc | | |
| RF-hHS51-mut-F1 | ttcctcatctgccaccatttttcaggtggatccacttt | | |
| RF-hHS51-mut-R1 | ttcctcatcgaacaccatttttcagatggaaccacttt | | |
| mHS51ctcf-mut-F1 | gaaaactgcagctgtctcgaaagatgctggttaaagatgt | | |
| mHS51ctcf-mut-R1 | gtaaggtccactacgaaaaatgccggctgggttatag | | |
| mHS51ctcf-mut-R2 | cgtaactgccagcgaaagatgctgtctgaatatc | | |
| mCTCFef-genotype-F3 | caggagctacgtatgcaggg | | |
| mCH-hHS51-F1 | gctgctagatccagcctgttactactgcttccagtg | | |
| mCH-hHS51-R1 | ccacccaagaaaataacctattgctcccagaggccctgag | | |
| mCH-hHS51-R2 | ttcctgctcctgaaactactgagcagccctgtatcc | | |
| Hglobin donor F1 | ggaagacaagatcctaggtttgga | | |
| Hglobin donor F2 | agatcctaggtttggaaagaacg | | |
| Hglobin donor R1 | agcctcaataagcttgcggt | | |
| Hglobin donor R2 | ctaaaagaagcccgggggag | | |
| Hglobin donor F3 | gcaggaagcctgtggctatt | | |
| Hglobin donor F4 | tctctggcaacttggagcac | | |
| Hglobin donor R3 | aggcagagccttgatgggat | | |
| Hglobin donor R4 | tggaagtgctaagcctgcat | | |
| PUC-globin-F | aaaacgacggccagtgaattcaagaacgtatttgatcca | | |
| globin-puc-F | ctagtgctagaactgagaag | | |
| PUC-globin--R | gaccatgattacgccaagcttagagctagttagccctcc | | |
| hglobin-donor-R5 | tgctgacacatccactgctat | | |
| mRF2-hglobin-F1 | ttgtgtgactgacctcggtcctggtttagtccctgggagc | | |
| mRF2-hglobin-R1 | tctcagttctagcactaggagtcacagctctggggaggta | | |
| R2-F1 | ggtgtcctgaagttcccagg | | |
| R2-F2 | ggaggcactgaaaaagtggc | | |
| R2-R1 | gtctaagtgcccaccagtcc | | |
| R2-R2 | gtccctccacagtggttgtc | | |
| R2-F1 | tgcttccctctactgtgtcct | | |
| R2-F2 | aagctagagctgtgctgagt | | |
| R2-R1 | agagggttagatgggggtgt | | |
| R2-R2 | ccaaacctgtgctgctcatt | | |
| R3-F1 | gggtaacccgacactcgttag | | |
| R3-F2 | cttgacagatggggaggctg | | |
| R3-R1 | aaagtcgggtacaggagcat | | |
| R3-R2 | cggtgagtacagaagcccag | | |
| R3-F1 | cccgcttctaacaaatgtccc | | |
| R3-F2 | tcttcccggaggaaagtgtt | | |
| R3-R1 | tggagaacatcatgggaacgc | | |
| R3-R2 | atcaaggtctgtagcccgag | | |
| a13c1-R3-F1 | cagttgccctccagacagtgctaaggctgtgctaaggctg | | |
| a13c1-R3-F2 | cccgggggtcatttctttatcctcttataacttcgtataatgtatgctatacgaagttatttggggttg | | |
| a13c1-R3-R1 | cccgggtctgaataggtatttctccgagagtgagtcttagggcagag | | |
| a13c1-R3-F3 | cagttgccctccagacagtgtaactgccttcctctggctg | | |
| a13c1-R3-R3 | cccgggtctgaataggtatttctccgcggcaacctggggtgcagga | | |
| a13c1-R3-F4 | cagttgccctccagacagtggctcattgaacagctagaag | | |
| a13c1-R3-R4 | cccgggtctgaataggtatttctccgggtgtgaatttctctgccca | | |
| ha13c1-geno-F1 | ccttcgatttccccccttcc | | |
| ha13c1-geno-R1 | aagatcatgccctttgcagc | | |
| ha13c1-geno-F2 | cggagcttaccatgaccgag | | |
| ha13c1-geno-R2 | tactcggtcatggtaagctcc | | |
| ha13c1-geno-F3 | ttagttgacaagttcatagggctaa | | |
| ha13c1-geno-R3 | tgtgaaaagacaacaccctca | | |
| ha13c1-geno-F4 | acaggtttagcacaatccaatc | | |
| ha13c1-geno-R4 | gaactcttacaactcaacaattg | | |
|  |  | | |
| Primers for screening CRISPR single-cell clones | | |  |
| Name | | Sequence |  |
| a13-ac1-F1 | | ttagttgacaagttcatagggctaa |  |
| a13-ac1-R1 | | tgtgaaaagacaacaccctca |  |
| a12-F1 | | gtgtaggttctggcctactgtat |  |
| a12-R1 | | ggatcgcttgcctgaggtatt |  |
| ac1-F1 | | gttagtgtctcggggcgtc |  |
| ac1-R1 | | cacgttccaccaacacgcta |  |
| ac1-F2 | | cctcagggcgttgtcctaga |  |
| ac1-R2 | | caacacgctacaccaacggt |  |
| pcdha-HS5-1-F1 | | ttaaagtgttttctccattcctgg |  |
| pcdha-HS5-1-R1 | | gttgtgaccatgtgagcatgtaat |  |
| pcdha-HS5-1-F2 | | tgtggaggtagctttactgtgac |  |
| pcdha-HS5-1-R2 | | agatgccaaattcaaagtgggac |  |
| LCR-globin-F1 | | ggaagacaagatcctaggtttgga |  |
| LCR-globin-R1 | | ctaaaagaagcccgggggag |  |
| LCR-globin-F2 | | tctctggcaacttggagcac |  |
| LCR-globin-R2 | | tggaagtgctaagcctgcat |  |
| ac2-F1 | | aaaagtctgcctgggtgtgg |  |
| ac2-R1 | | gccaacttcagcttttcagca |  |
| ac2-F2 | | gaaggtgggcgaggtgag |  |
| ac2-R2 | | acacaaattctcagatcaatcaaca |  |
|  | | |  |
| Primers for mouse genotyping | | |  |
| Name | | Sequence |  |
| aF | | caatggaggcaatcccttctg |  |
| aR | | gaacatgggctcagagactagg |  |
| bF | | gttcctttgtcaggtgaaaatctc |  |
| bR | | gggtggtagtgagggattattctag |  |
| cF | | ctctaaaaagtggttccatctgc |  |
| cR | | cttccagcactttcctcatcg |  |
| be-F7 | | acaggataatgggttctggagc |  |
| be-F5 | | agccagaccagcatagcaaat |  |
| be-R3 | | tccagattacgagctgagcg |  |
| M-A-DEL-JD-F1 | | ggactggctcaatcctcacattc |  |
| M-A-DEL-JD-R1 | | accgttctctggccacaatttt |  |
| M-A-WT-JD-R1 | | accgttctctggccacaagggg |  |
| M-BC-DEL-JD-F1 | | catgacgtctaggcctccaa |  |
| M-BC-DEL-JD-R1 | | gtgaaggatgcacaggaacagt |  |
| M-BC-WT-JD-R1 | | tcgctttgcctggcatagct |  |
| M-D-DEL-JD-F1 | | cagaggaggttgatccaggtat |  |
| M-D-DEL-JD-R1 | | tcccccaaacttcatggtcaat |  |
| M-D-WT-JD-F1 | | tctagatgaagaaggatcagctgc |  |
| M-D-WT-JD-R1 | | cattttgtgttgatgacgcagg |  |
| M-E-DEL-JD-F1 | | ttgtgaggaaaccaagtccct |  |
| M-E-DEL-JD-R1 | | tccagattacgagctgagcg |  |
| M-E-WT-JD-R1 | | gcacctaaaaaccggctgtg |  |
|  | |  |  |
| Primers for SNP identification | | |  |
| Name | | Sequence |  |
| a6-snp-F1 | | acatcttcacggtgtctgcg |  |
| a6-snp-F2 | | cgcgttgtcgagctacatttc |  |
| a6-snp-R1 | | tgattctccgccttacccatca |  |
| a12-snp-F1 | | acccacattccaatcattcacg |  |
| a12-snp-R1 | | aagtccttcgtctggatcgga |  |
| a12-snp-F2 | | ccacgtggtggcgaaagt |  |
| a12-snp-R2 | | gcgatgatgaggtacacgttaata |  |
| ac1-snp-F1 | | cgtcttgacctacgacctgg |  |
| ac1-snp-R1 | | atcccggccttctacttgga |  |
| ac1-snp-F2 | | tgcagttaagcagagggtgg |  |
| ac1-snp-R2 | | gagtgccttacacatctgttgc |  |
| ac2-snp-F1 | | tcctgaattgcgcttggaga |  |
| ac2-snp-R1 | | gcacccagatggttgatgcg |  |
| ac2-snp-F2 | | gagcgcattgatcgggagg |  |
| ac2-snp-R2 | | tccaaagccattcagtcgga |  |
| acon3-snp-F1 | | tcattgctgctacagaagtgct |  |
| acon3-snp-R1 | | caatcctgcagagctgaagttac |  |
|  | |  |  |

| Primers for 4C | |
| --- | --- |
| Name | Sequence |
| Adapter-U | gacgtgtgctcttccgatctgnnnnnnnn-nh2 |
| Adapter-L | p-cagatcggaagagcacacgtc-nh2 |
| biotin-HS5-1 | 5’ biotin-tgctttctcattccccgttg |
| qhr-HS5-1-F1 | aatgatacggcgaccaccgagatctacactctttccctacacgacgctcttccgatctttttggcggcgacaaattcg |
| qhr-HS5-1-F2 | aatgatacggcgaccaccgagatctacactctttccctacacgacgctcttccgatctgttttggcggcgacaaattcg |
| qhr-HS5-1-F3 | aatgatacggcgaccaccgagatctacactctttccctacacgacgctcttccgatctagttttggcggcgacaaattcg |
| qhr-HS5-1-F4 | aatgatacggcgaccaccgagatctacactctttccctacacgacgctcttccgatctcagttttggcggcgacaaattcg |
| qhr-HS5-1-F5 | aatgatacggcgaccaccgagatctacactctttccctacacgacgctcttccgatctcacgttttggcggcgacaaattcg |
| qhr-HS5-1-F6 | aatgatacggcgaccaccgagatctacactctttccctacacgacgctcttccgatctagacgttttggcggcgacaaattcg |
| qhr-HS5-1-F7 | aatgatacggcgaccaccgagatctacactctttccctacacgacgctcttccgatctgcgttttggcggcgacaaattcg |
| biotin-HS7 | 5’ biotin-cagcaggactgccaagagtt |
| qhr-HS7-F1 | aatgatacggcgaccaccgagatctacactctttccctacacgacgctcttccgatcttgactcagcctaaactccaggag |
| qhr-HS7-F2 | aatgatacggcgaccaccgagatctacactctttccctacacgacgctcttccgatctctgactcagcctaaactccaggag |
| qhr-HS7-F3 | aatgatacggcgaccaccgagatctacactctttccctacacgacgctcttccgatctgctgactcagcctaaactccaggag |
| biotin-a6 | 5’ biotin-ggattaataaattccggaacatacag |
| qhr-a6-F1 | aatgatacggcgaccaccgagatctacactctttccctacacgacgctcttccgatctacagtattttatctttatgcggaag |
| qhr-a6-F2 | aatgatacggcgaccaccgagatctacactctttccctacacgacgctcttccgatctgacagtattttatctttatgcggaag |
| qhr-a6-F3 | aatgatacggcgaccaccgagatctacactctttccctacacgacgctcttccgatctagacagtattttatctttatgcggaag |
| qhr-a6-F4 | aatgatacggcgaccaccgagatctacactctttccctacacgacgctcttccgatctcagacagtattttatctttatgcggaag |
| biotin-a12 | 5’ biotin-ccgcacccacattccaatca |
| qhr-a12-F1 | aatgatacggcgaccaccgagatctacactctttccctacacgacgctcttccgatcttccaatcattcacggaataggatc |
| qhr-a12-F2 | aatgatacggcgaccaccgagatctacactctttccctacacgacgctcttccgatctgtccaatcattcacggaataggatc |
| qhr-a12-F3 | aatgatacggcgaccaccgagatctacactctttccctacacgacgctcttccgatctagtccaatcattcacggaataggatc |
| qhr-a12-F4 | aatgatacggcgaccaccgagatctacactctttccctacacgacgctcttccgatctcagtccaatcattcacggaataggatc |
| biotin-mb3 | 5’ biotin-aaggagaagggcttcgtaatag |
| qhr-mb3-F1 | aatgatacggcgaccaccgagatctacactctttccctacacgacgctcttccgatctaatagccaatttagcgacggatc |
| qhr-mb3-F2 | aatgatacggcgaccaccgagatctacactctttccctacacgacgctcttccgatctctaatagccaatttagcgacggatc |
| biotin-mhs51 | 5’ biotin-gtggctttgttactctaggaacag |
| qhr-mhs51-F1 | aatgatacggcgaccaccgagatctacactctttccctacacgacgctcttccgatctggaggttaaagcaaagactaagatc |
| qhr-mhs51-F2 | aatgatacggcgaccaccgagatctacactctttccctacacgacgctcttccgatctatggaggttaaagcaaagactaagatc |
| biotin-mhs7 | 5’ biotin-aaatgattacaggcgcttccg |
| qhr-mhs7-F1 | aatgatacggcgaccaccgagatctacactctttccctacacgacgctcttccgatctctcccttgaactgttcctgatc |
| qhr-mhs7-F2 | aatgatacggcgaccaccgagatctacactctttccctacacgacgctcttccgatctatctcccttgaactgttcctgatc |
| biotin-mpcdha4 | 5’ biotin-cactgtgctggatgccaatg |
| qhr-mpcdha4-f | aatgatacggcgaccaccgagatctacactctttccctacacgacgctcttccgatctaatgctccagtttttgacagatc |
| biotin-mpcdha9 | 5’ biotin-gatatagttcgctgtttctcaggg |
| qhr-mpcdha9-f | aatgatacggcgaccaccgagatctacactctttccctacacgacgctcttccgatcttaggaagtagctacgttcggag |
| biotin-mpcdha12 | 5’ biotin-gcagtgaagagcgacatcatg |
| qhr-mpcdha12-F | aatgatacggcgaccaccgagatctacactctttccctacacgacgctcttccgatctcatgaatgtgtaagtgtagcgatc |
| biotin-mpcdhac1 | 5’ biotin-agcctaagcattagccagcac |
| qhr-mpcdhac1-F | aatgatacggcgaccaccgagatctacactctttccctacacgacgctcttccgatcttttcgcctggacatgggatc |
| biotin-3'HS1 | 5’ biotin-ccactaggggtcagaagtagttcat |
| qhr-3'HS1-F1 | aatgatacggcgaccaccgagatctacactctttccctacacgacgctcttccgatcttcttccctccctacttcagtgatg |
| biotin-hbg2 | 5’ biotin-aagatttttattggtatgctggtacag |
| qhr-hbg2-F1 | aatgatacggcgaccaccgagatctacactctttccctacacgacgctcttccgatctaggtgaacccaaattagggtctc |
| biotin-hs2 | 5’ biotin-cagttacccacacaggtgaaccc |
| qhr-hs2-F1 | aatgatacggcgaccaccgagatctacactctttccctacacgacgctcttccgatctggaatgtttctttcctctcaggatc |
| biotin-hs5 | 5’ biotin-ccagtcataagcacagactacgtg |
| qhr-hs5-F1 | aatgatacggcgaccaccgagatctacactctttccctacacgacgctcttccgatctgaaatgagaggagcaagaaagagg |
| qhr-m-se-a-F1 | aatgatacggcgaccaccgagatctacactctttccctacacgacgctcttccgatctgcccttacagtagtgtagaagatc |
| qhr-m-se-a-F2 | aatgatacggcgaccaccgagatctacactctttccctacacgacgctcttccgatctgagcccttacagtagtgtagaagatc |
| qhr-m-se-a-F3 | aatgatacggcgaccaccgagatctacactctttccctacacgacgctcttccgatctgacgcccttacagtagtgtagaagatc |
| qhr-m-se-a-F4 | aatgatacggcgaccaccgagatctacactctttccctacacgacgctcttccgatctgactgcccttacagtagtgtagaagatc |
| biotin-m-se-a | 5’ biotin-ccagtgcctccttgtgcatag |
| qhr-m-se-bc-f1 | aatgatacggcgaccaccgagatctacactctttccctacacgacgctcttccgatctgaggcttatgggccttacagatc |
| qhr-m-se-bc-f2 | aatgatacggcgaccaccgagatctacactctttccctacacgacgctcttccgatctacgaggcttatgggccttacagatc |
| qhr-m-se-bc-f3 | aatgatacggcgaccaccgagatctacactctttccctacacgacgctcttccgatctactgaggcttatgggccttacagatc |
| qhr-m-se-bc-f4 | aatgatacggcgaccaccgagatctacactctttccctacacgacgctcttccgatctactcgaggcttatgggccttacagatc |
| biotin-m-se-bc | 5’ biotin-aactatgctcagggcctctgg |
| qhr-m-se-d-F1 | aatgatacggcgaccaccgagatctacactctttccctacacgacgctcttccgatctacagcaggcagcagctgatc |
| qhr-m-se-d-F2 | aatgatacggcgaccaccgagatctacactctttccctacacgacgctcttccgatctgaacagcaggcagcagctgatc |
| qhr-m-se-d-F3 | aatgatacggcgaccaccgagatctacactctttccctacacgacgctcttccgatctgacacagcaggcagcagctgatc |
| qhr-m-se-d-F4 | aatgatacggcgaccaccgagatctacactctttccctacacgacgctcttccgatctgactacagcaggcagcagctgatc |
| biotin-m-se-d | 5’ biotin-tccctgccagctgagctaag |
| qhr-m-se-e-F1 | aatgatacggcgaccaccgagatctacactctttccctacacgacgctcttccgatctccagattctgaacccgacagatc |
| qhr-m-se-e-F2 | aatgatacggcgaccaccgagatctacactctttccctacacgacgctcttccgatctagccagattctgaacccgacagatc |
| qhr-m-se-e-F3 | aatgatacggcgaccaccgagatctacactctttccctacacgacgctcttccgatctagtccagattctgaacccgacagatc |
| qhr-m-se-e-F4 | aatgatacggcgaccaccgagatctacactctttccctacacgacgctcttccgatctagtaccagattctgaacccgacagatc |
| biotin-m-se-e | 5’ biotin-ttagaggacacgctgccgtc |
| qhr-m-se-f-F1 | aatgatacggcgaccaccgagatctacactctttccctacacgacgctcttccgatctggagcactgttgcttagagattctc |
| qhr-m-se-f-F2 | aatgatacggcgaccaccgagatctacactctttccctacacgacgctcttccgatctacggagcactgttgcttagagattctc |
| biotin-m-se-f | 5’ biotin-ctccatgtgccatctggtgg |
| qhr-m-b4-F | aatgatacggcgaccaccgagatctacactctttccctacacgacgctcttccgatctaagaaggtagcattcactttggatc |
| biotin-m-b4 | 5’ biotin-ccattaccgaatcagaacttacacc |
| qhr-m-b5-F | aatgatacggcgaccaccgagatctacactctttccctacacgacgctcttccgatcttgcagtttcctgtgcttttggatc |
| biotin-m-b5 | 5’ biotin-gtgggaaaattgcagaaatccatc |
| qhr-m-b6-F | aatgatacggcgaccaccgagatctacactctttccctacacgacgctcttccgatctatgctgaatgcttccccgaa |
| biotin-m-b6 | 5’ biotin-aggatttgtaaaacaaggggctc |
| qhr-m-b9-F1 | aatgatacggcgaccaccgagatctacactctttccctacacgacgctcttccgatcttcagagggaattgtcttttggaaa |
| qhr-m-b9-F2 | aatgatacggcgaccaccgagatctacactctttccctacacgacgctcttccgatctagtcagagggaattgtcttttggaaa |
| qhr-m-b9-F3 | aatgatacggcgaccaccgagatctacactctttccctacacgacgctcttccgatctagctcagagggaattgtcttttggaaa |
| qhr-m-b9-F4 | aatgatacggcgaccaccgagatctacactctttccctacacgacgctcttccgatctagcatcagagggaattgtcttttggaaa |
| biotin-m-b9 | 5’ biotin-gaacaaagttgcttgcttaaaatca |
| qhr-m-b10-F | aatgatacggcgaccaccgagatctacactctttccctacacgacgctcttccgatctgaagaagaaggtatttgagcgtgat |
| biotin-m-b10 | 5’ biotin-atatacccagaaagccactgaaagc |
| qhr-m-b13-F1 | aatgatacggcgaccaccgagatctacactctttccctacacgacgctcttccgatctgtcaccgggaacctgtacgatc |
| qhr-m-b13-F2 | aatgatacggcgaccaccgagatctacactctttccctacacgacgctcttccgatctatgtcaccgggaacctgtacgatc |
| qhr-m-b13-F3 | aatgatacggcgaccaccgagatctacactctttccctacacgacgctcttccgatctatcgtcaccgggaacctgtacgatc |
| qhr-m-b13-F4 | aatgatacggcgaccaccgagatctacactctttccctacacgacgctcttccgatctatcagtcaccgggaacctgtacgatc |
| biotin-m-b13 | 5’ biotin-aatacggaaccatttgctgctg |
| qhr-m-b16-F | aatgatacggcgaccaccgagatctacactctttccctacacgacgctcttccgatctaacaaactacatttgcagctggatc |
| biotin-m-b16 | 5’ biotin-cggagaggagctaaggtcatttcta |
| qhr-m-b17-F | aatgatacggcgaccaccgagatctacactctttccctacacgacgctcttccgatctactgtgagatagtgctgtcacgatc |
| biotin-m-b17 | 5’ biotin-gcataggcgagtgtgaaacctta |
| qhr-m-b18-F | aatgatacggcgaccaccgagatctacactctttccctacacgacgctcttccgatctgcttccacccctagccctaga |
| biotin-m-b18 | 5’ biotin-gtgagaatccagaaacaagtgctg |
| qhr-m-b20-F | aatgatacggcgaccaccgagatctacactctttccctacacgacgctcttccgatctctcctgaatgagcgactggatc |
| biotin-m-b20 | 5’ biotin-gaggccagggtagtgtcagatg |
| qhr-m-b21-F1 | aatgatacggcgaccaccgagatctacactctttccctacacgacgctcttccgatctttaaagatatggatgtgggtgatc |
| qhr-m-b21-F2 | aatgatacggcgaccaccgagatctacactctttccctacacgacgctcttccgatctgattaaagatatggatgtgggtgatc |
| biotin-m-b21 | 5’ biotin-tctgtgccagaagaaatggagac |
| qhr-m-b22-F | aatgatacggcgaccaccgagatctacactctttccctacacgacgctcttccgatcttcatctgagacagcaaggagtgatc |
| biotin-m-b22 | 5’ biotin-gctgaataagcaagacaggattttc |
| qhr-m-ga3-F | aatgatacggcgaccaccgagatctacactctttccctacacgacgctcttccgatctcggctggtttcaggcagatc |
| biotin-m-ga3 | 5’ biotin-accgcaggagctttgttgtg |
| qhr-m-gb1-F | aatgatacggcgaccaccgagatctacactctttccctacacgacgctcttccgatcttggggagtctcgccaaggatc |
| biotin-m-gb1 | 5’ biotin-cccatccactactccattccg |
| qhr-m-ga4-F1 | aatgatacggcgaccaccgagatctacactctttccctacacgacgctcttccgatctgcacatccagggagaagtgatc |
| qhr-m-ga4-F2 | aatgatacggcgaccaccgagatctacactctttccctacacgacgctcttccgatctctgcacatccagggagaagtgatc |
| qhr-m-ga4-F3 | aatgatacggcgaccaccgagatctacactctttccctacacgacgctcttccgatctggagcacatccagggagaagtgatc |
| qhr-m-ga4-F4 | aatgatacggcgaccaccgagatctacactctttccctacacgacgctcttccgatctggtcgcacatccagggagaagtgatc |
| biotin-m-ga4 | 5’ biotin-gatacttaatcccatccggtcca |
| qhr-m-ga6-F | aatgatacggcgaccaccgagatctacactctttccctacacgacgctcttccgatctaacaactcaccttttaaggcagatc |
| biotin-m-ga6 | 5’ biotin-ctaaaacattctccaccagaggatt |
| qhr-m-ga10-F | aatgatacggcgaccaccgagatctacactctttccctacacgacgctcttccgatctaaccgggtgaaacacactgg |
| biotin-m-ga10 | 5’ biotin-ggtcacagaaagaagaaaagaaccg |
| qhr-m-gb7-F1 | aatgatacggcgaccaccgagatctacactctttccctacacgacgctcttccgatctgcctttacttaccttccacggatc |
| qhr-m-gb7-F2 | aatgatacggcgaccaccgagatctacactctttccctacacgacgctcttccgatctacgcctttacttaccttccacggatc |
| biotin-m-gb7 | 5’ biotin-ctcttgtgggacaaagatgtaggac |
| qhr-m-gb8-F | aatgatacggcgaccaccgagatctacactctttccctacacgacgctcttccgatctaaagctcctcagaggtgggatc |
| biotin-m-gb8 | 5’ biotin-gaatttgactgttttccagtgttgc |
| qhr-m-ga12-F | aatgatacggcgaccaccgagatctacactctttccctacacgacgctcttccgatctaaagcagattctggttcaggtgatc |
| biotin-m-ga12 | 5’ biotin-actgtcttgctccaaggcttca |
| qhr-m-gb2-F | aatgatacggcgaccaccgagatctacactctttccctacacgacgctcttccgatctcctgttgcttttgttccacgg |
| biotin-m-gb2 | 5’ biotin-gtggctgcaggtactgtcgc |
| qhr-m-gb6-F1 | aatgatacggcgaccaccgagatctacactctttccctacacgacgctcttccgatctgtggaacattctgtattatgtcgtga |
| qhr-m-gb6-F2 | aatgatacggcgaccaccgagatctacactctttccctacacgacgctcttccgatctctgtggaacattctgtattatgtcgtga |
| qhr-m-gb6-F3 | aatgatacggcgaccaccgagatctacactctttccctacacgacgctcttccgatctctcgtggaacattctgtattatgtcgtga |
| qhr-m-gb6-F4 | aatgatacggcgaccaccgagatctacactctttccctacacgacgctcttccgatctcaagtggaacattctgtattatgtcgtga |
| biotin-m-gb6 | 5’ biotin-ggggttacagtggatacttatgtgc |
| qhr-m-ga2-F1 | aatgatacggcgaccaccgagatctacactctttccctacacgacgctcttccgatctggaggccaaagctggacagatc |
| qhr-m-ga2-F2 | aatgatacggcgaccaccgagatctacactctttccctacacgacgctcttccgatctacggaggccaaagctggacagatc |
| biotin-m-ga2 | 5’ biotin-ccagagcagaatgctggtcct |
| qhr-m-ga7-F1 | aatgatacggcgaccaccgagatctacactctttccctacacgacgctcttccgatctctctcagggtgtgagcgagatc |
| qhr-m-ga7-F2 | aatgatacggcgaccaccgagatctacactctttccctacacgacgctcttccgatctagctctcagggtgtgagcgagatc |
| biotin-m-ga7 | 5’ biotin-acacaactgggttctgaggca |
| qhr-m-ga11-f1 | aatgatacggcgaccaccgagatctacactctttccctacacgacgctcttccgatctacactcaccttttccgtggatc |
| qhr-m-ga11-f2 | aatgatacggcgaccaccgagatctacactctttccctacacgacgctcttccgatctgcacactcaccttttccgtggatc |
| biotin-m-ga11 | 5’ biotin-gtgtctgttgcggagaaaactgt |
| qhr-m-gc3-F1 | aatgatacggcgaccaccgagatctacactctttccctacacgacgctcttccgatctttccaccatcattcactatgagatc |
| qhr-m-gc3-F2 | aatgatacggcgaccaccgagatctacactctttccctacacgacgctcttccgatctccttccaccatcattcactatgagatc |
| biotin-m-gc3 | 5’ biotin-ggtgggagttttgcttctgct |
| P7-R1 | caagcagaagacggcatacgagatacatcggtgactggagttcagacgtgtgctcttccgatct |
| P7-R2 | caagcagaagacggcatacgagattggtcagtgactggagttcagacgtgtgctcttccgatct |
| P7-R3 | caagcagaagacggcatacgagatcactgtgtgactggagttcagacgtgtgctcttccgatct |
| P7-R4 | caagcagaagacggcatacgagatattggcgtgactggagttcagacgtgtgctcttccgatct |
| P7-R5 | caagcagaagacggcatacgagatgatctggtgactggagttcagacgtgtgctcttccgatct |
| P7-R6 | caagcagaagacggcatacgagattacaaggtgactggagttcagacgtgtgctcttccgatct |
| 4C-HS5-1-F1 | aatgatacggcgaccaccgagatctacactctttccctacacgacgctcttccgatctttttggcggcgacaaattcg |
| 4C-HS5-1-F2 | aatgatacggcgaccaccgagatctacactctttccctacacgacgctcttccgatctgttttggcggcgacaaattcg |
| 4C-HS5-1-F3 | aatgatacggcgaccaccgagatctacactctttccctacacgacgctcttccgatctagttttggcggcgacaaattcg |
| 4C-HS5-1-R1 | caagcagaagacggcatacgagattcaggtgtgactggagttcagacgtgtgctcttccgatctgaaaatctctgcagcgagt |
| 4C-HS5-1-R2 | caagcagaagacggcatacgagattggtcagtgactggagttcagacgtgtgctcttccgatcttgaaaatctctgcagcgagt |
| 4C-HS5-1-R3 | caagcagaagacggcatacgagatcactgtgtgactggagttcagacgtgtgctcttccgatctctgaaaatctctgcagcgagt |
| 4C-RF-F1 | aatgatacggcgaccaccgagatctacactctttccctacacgacgctcttccgatcttcaatagcattttcctcatctgc |
| 4C-RF-F2 | aatgatacggcgaccaccgagatctacactctttccctacacgacgctcttccgatctctcaatagcattttcctcatctgc |
| 4C-RF-R1 | caagcagaagacggcatacgagatacatcggtgactggagttcagacgtgtgctcttccgatctatggaaccactttttagagcacg |
| 4C-RF-R2 | caagcagaagacggcatacgagatcactgtgtgactggagttcagacgtgtgctcttccgatctgatggaaccactttttagagcacg |
| 4C-RRFFu-F1 | aatgatacggcgaccaccgagatctacactctttccctacacgacgctcttccgatctgtcctagatggctctaaaaagtagg |
| 4C-RRFFu-F2 | aatgatacggcgaccaccgagatctacactctttccctacacgacgctcttccgatctcgtcctagatggctctaaaaagtagg |
| 4C-RRFFu-r1 | caagcagaagacggcatacgagatacatcggtgactggagttcagacgtgtgctcttccgatctccagctccttgggatttcagt |
| 4C-RRFFu-r2 | caagcagaagacggcatacgagatcgtgatgtgactggagttcagacgtgtgctcttccgatctaccagctccttgggatttcagt |
| 4C-RRFFd-F1 | aatgatacggcgaccaccgagatctacactctttccctacacgacgctcttccgatctccccctctatctctggtattgt |
| 4C-RRFFd-F2 | aatgatacggcgaccaccgagatctacactctttccctacacgacgctcttccgatcttccccctctatctctggtattgt |
| 4C-RRFFd-F3 | aatgatacggcgaccaccgagatctacactctttccctacacgacgctcttccgatctatccccctctatctctggtattgt |
| 4C-RRFFd-r1 | caagcagaagacggcatacgagatacatcggtgactggagttcagacgtgtgctcttccgatctacagcttcgtaggacggaca |
| 4C-RRFFd-r2 | caagcagaagacggcatacgagatgcctaagtgactggagttcagacgtgtgctcttccgatctcacagcttcgtaggacggaca |
| 4C-RRFFd-r3 | caagcagaagacggcatacgagattggtcagtgactggagttcagacgtgtgctcttccgatctacagcttcgtaggacggaca |
| 4C-FFRRu-F1 | aatgatacggcgaccaccgagatctacactctttccctacacgacgctcttccgatcttcaagctggactagtattcctttg |
| 4C-FFRRu-F2 | aatgatacggcgaccaccgagatctacactctttccctacacgacgctcttccgatctatcaagctggactagtattcctttg |
| 4C-FFRRu-F3 | aatgatacggcgaccaccgagatctacactctttccctacacgacgctcttccgatctcgtcaagctggactagtattcctttg |
| 4C-FFRRu-r1 | caagcagaagacggcatacgagatacatcggtgactggagttcagacgtgtgctcttccgatctgatacagaaaagtctgcctggg |
| 4C-FFRRu-r2 | caagcagaagacggcatacgagatcactgtgtgactggagttcagacgtgtgctcttccgatctcgatacagaaaagtctgcctggg |
| 4C-FFRRu-r3 | caagcagaagacggcatacgagatgcctaagtgactggagttcagacgtgtgctcttccgatctgatacagaaaagtctgcctggg |

| **Table S2.** CRISPR deletion and inversion mice. | | |
| --- | --- | --- |
| Name | Analyzed mice | F0 mice with targeted modification |
| HS5-1 deletion | 42 | 26 |
| HS5-1 inversion | 35 | 6 |
| HS5-1a deletion | 11 | 1 |
| HS5-1b deletion | 9 | 1 |
| HS5-1b inversion | 23 | 2 |
| a deletion | 2 | 1 |
| bc deletion | 33 | 2 |
| d deletion | 14 | 3 |
| e deletion | 5 | 1 |
| de deletion | 16 | 3 |
| b-e deletion | 22 | 5 |
| Total | 212 | 51 |

**Table S3.** CRISPR single-cell clones.

| Name | Screened single-cell clones | Homozygous CRISPR clones |
| --- | --- | --- |
| F insertion | 83 | 3 |
| F mutation | 52 | 2 |
| FF insertion | 86 | 2 |
| FF mutation | 31 | 2 |
| R1 insertion | 64 | 2 |
| R1 mutation | 81 | 2 |
| R2 insertion | 72 | 2 |
| R2 mutation | 76 | 2 |
| R3 insertion | 43 | 2 |
| R3 mutation | 43 | 2 |
| ac1 CBS del | 37 | 8 |
| a12 CBS del | 95 | 21 |
| RF insertion | 115 | 5 |
| RF mutation | 70 | 5 |
| RRFF insertion | 99 | 3 |
| RRFF mutation | 276 | 3 |
| FFRR insertion (RR) | 74 | 2 |
| FFRR mutation (RR) | 68 | 5 |
| FFRR insertion (FF) | 71 | 3 |
| FFRR mutation (FF) | 32 | 2 |
| RF2 insertion | 275 | 1 |
| RF2 mutation | 105 | 1 |
| Total | 1,948 | 80 |

**Table S4.** Mapping statistics of the 5C data.

| Name | numRawReads | numQCPassedReads | side1Mapped | side2Mapped | bothSideMapped |
| --- | --- | --- | --- | --- | --- |
| WT1 | 2,086,393 | 2,086,393 | 2,013,333 | 2,013,234 | 1,951,615 |
|  |  | 100.00% | 96.50% | 96.49% | 93.54% |
| WT2 | 2,184,742 | 2,184,742 | 2,176,832 | 2,177,198 | 2,137,156 |
|  |  | 100.00% | 99.64% | 99.65% | 97.82% |
| HS5-1a del1 | 1,946,406 | 1,946,406 | 1,939,809 | 1,938,108 | 1,901,771 |
|  |  | 100.00% | 99.66% | 99.57% | 97.71% |
| HS5-1a del2 | 2,013,637 | 2,013,637 | 1,996,275 | 1,996,010 | 1,934,313 |
|  |  | 100.00% | 99.14% | 99.12% | 96.06% |
| HS5-1b del1 | 1,999,152 | 1,999,152 | 1,988,285 | 1,988,181 | 1,951,494 |
|  |  | 100.00% | 99.46% | 99.45% | 97.62% |
| HS5-1b del2 | 1,919,503 | 1,919,503 | 1,908,474 | 1,904,460 | 1,865,225 |
|  |  | 100.00% | 99.43% | 99.22% | 97.17% |
| HS5-1 del1 | 2,114,603 | 2,114,603 | 2,101,714 | 2,104,719 | 2,040,642 |
|  |  | 100.00% | 99.39% | 99.53% | 96.50% |
| HS5-1 del2 | 2,015,084 | 2,015,084 | 2,002,136 | 2,002,247 | 1,937,030 |
|  |  | 100.00% | 99.36% | 99.36% | 96.13% |
| HS5-1 inv1 | 1,900,123 | 1,900,123 | 1,892,202 | 1,890,938 | 1,853,703 |
|  |  | 100.00% | 99.58% | 99.52% | 97.56% |
| HS5-1 inv2 | 1,972,598 | 1,972,598 | 1,963,254 | 1,961,278 | 1,923,382 |
|  |  | 100.00% | 99.53% | 99.43% | 97.51% |
| BAC1 | 2,068,370 | 2,068,370 | 2,062,807 | 2,062,642 | 1,998,383 |
|  |  | 100.00% | 99.73% | 99.72% | 96.62% |
| BAC2 | 1,908,206 | 1,908,206 | 1,903,957 | 1,903,740 | 1,862,355 |
|  |  | 100.00% | 99.78% | 99.77% | 97.60% |

**Table S5.** Pearson correlations between 5C replicates.

| Rep 1 | Rep 2 | R^2^ |
| --- | --- | --- |
| WT1 | WT2 | 0.967081 |
| HS5-1a del1 | HS5-1a del2 | 0.987664 |
| HS5-1b del1 | HS5-1b del2 | 0.99251 |
| HS5-1 del1 | HS5-1 del2 | 0.981288 |
| HS5-1 inv1 | HS5-1 inv2 | 0.986553 |
| BAC1 | BAC2 | 0.826681 |
