# Supplementary material for: Tandem CTCF sites function as insulators to balance spatial chromatin contacts and topological enhancer-promoter selection
Source: Genome Biol. 2020 Mar 23;21:75. doi: 10.1186/s13059-020-01984-7 (PMC7087399; doi:10.1186/s13059-020-01984-7)
Supplement: Supplementary file 3 — Additional file 3: Review history. [file 13059_2020_1984_MOESM3_ESM.docx]

Review History

***This manuscript was previously reviewed at another journal. Our reviewer primarily assessed author responses to the previous round(s) of review.***

**First round of review**

**Reviewer 1**

**Are you able to assess all statistics in the manuscript, including the appropriateness of statistical tests used?**

Yes, and I have assessed the statistics in my report.

**Comments to author:**

This study provides a very meticulous perturbation analysis of CTCF sites in the Pcdh locus, primarily reporting data from 4C and some interesting 3D modelling results. I think these data deserve publication in Genome Biology with minor revisions, as it clearly doesn't make sense for such a bulk of evidence to be withheld from the chromosomal topology and gene regulation communities.

Main comments:

1. With regard to the previous rounds of reviews, I believe that many, if not all, of the outstanding issues could be adequately addressed by simply toning down the claims in the text or mentioning the limitations of the experiments and analyses more explicitly. I would recommend that the authors do just that.

2. I would personally like to see in the text a more explicit interpretation/hypothesis as to how exactly the insulators can promote long-range chromosomal interactions (as is both observed and predicted by the model).

Minor comments:

1. Line 51: It would be fair to also mention and cite studies showing that in some cases at least, TAD perturbations don't lead to gene expression changes.

2. I have an issue with the term "topological" regulation and its derivatives, such as "regulates non-topologically". I don't fully understand what the authors mean: topologically as opposed to "in cis" or as opposed to "in 1D"? Suggest rephrasing.

3. I am personally not a fan of verbless section headings, as it's unclear from them what the message is - only what was perturbed.

**Authors Response**

**Point-by-point responses to the reviewers’ comments:**

Reviewer #1: This study provides a very meticulous perturbation analysis of CTCF sites in the Pcdh locus, primarily reporting data from 4C and some interesting 3D modelling results. I think these data deserve publication in Genome Biology with minor revisions, as it clearly doesn't make sense for such a bulk of evidence to be withheld from the chromosomal topology and gene regulation communities.

*We appreciate these positive and decisive comments by your trusted Referee.*

Main comments:

1. With regard to the previous rounds of reviews, I believe that many, if not all, of the outstanding issues could be adequately addressed by simply toning down the claims in the text or mentioning the limitations of the experiments and analyses more explicitly. I would recommend that the authors do just that.

*Thanks for the suggestion. We have revised the following to tone down the claims in the text or mentioning the limitations more explicitly:*

*As suggested by editors, we have removed “genome” from the title. This reflects the fact that we only performed experiments on three loci of large gene clusters.*

*As stated above for the abstract, we added one sentence to indicate that our findings are based on loop extrusion and toned down the novelty.*

*For the section title on Page 5, in order to mention the limitation of our experiments in cellular model in vitro not in vivo, we have changed it from: “Exogenous CTCF sites as protocadherin insulators.” to “Exogenous directional CTCF sites function as protocadherin insulators in cellular model in vitro”.*

*On the 4th line from the bottom of Page 7, we have changed from: “even passed through the intervening convergent CBS sites” to: “presumably by cohesin sliding through the oncoming convergent CTCF sites”, to tone it down and acknowledge prior work on cohesin.*

*On top of Page 13, we have changed from: “Insulators for enhancers with no CTCF site” to “Insulators for Pcdh alpha and beta-globin enhancers with no CTCF site”, to indicate the restriction of our work on Pcdh and beta-globin.*

*On the bottom of Page 17, we added one sentence to show the limitation of our work: “However, since there are numerous gene clusters and hundreds of thousands CTCF sites in mammalian genomes, whether all tandem CTCF sites functions in a similar manner in vivo waits further studies.”*

2. I would personally like to see in the text a more explicit interpretation/hypothesis as to how exactly the insulators can promote long-range chromosomal interactions (as is both observed and predicted by the model).

*As stated above we have added a new Panel of Fig. 5n to help our interpretation/hypothesis. As for the text, we added the following paragraph to the bottom of Page 16 and top of the Page 17: “We posit a general mechanism for the Hulu model by which tandem directional CTCF sites function as topological insulators in the context of the cohesin “loop extrusion” (Fig. 5m,n). Because each CBS element has permeability for cohesin sliding [20], continuous active chromatin loop extrusion by cohesin in an ATP-dependent manner bridges inner convergent CTCF sites first (Fig. 5n). After sliding through the inner proximal CTCF sites, cohesin will then stall at the intermediate tandem CTCF sites (Fig. 5n). Finally, cohesin will reach the outer distal CTCF sites (Fig. 5n). Based on our experimental observation and mathematical prediction in the clustered Pcdh and Ig genes, the two “heads” of cohesin are stalled or anchored at CTCF sites in the two arrays of convergent tandem CTCF sites, resulting in long-range chromatin interactions between proximal-proximal CBS elements as well as between distal-distal CBS elements (Fig. 5m,n). In other words, two “heads” of cohesin complex anchor proximal-proximal or distal-distal CBSs through continuous active loop extrusion of chromatin fibers which are asymmetrically blocked by permeable CTCF insulators. The functional consequences of these interactions caused by tandem CTCF insulators are the decreased proximal chromatin interactions and increased distal chromatin interactions. Thus, tandem directional CTCF sites function as topological insulators to balance higher-order chromatin contacts and promoter choice, eliminating bias of spatial chromatin accessibilities between proximal and distal promoters by remote enhancers.”*

Minor comments:

1. Line 51: It would be fair to also mention and cite studies showing that in some cases at least, TAD perturbations don't lead to gene expression changes.

*Thanks for the suggestion, we added one sentence to the top of Page 4: “Recent TAD (topologically associated domain) perturbations by targeted degradation of CTCF or cohesin revealed that loss of chromatin loops genome wide differentially affect gene expression [21, 22].”*

2. I have an issue with the term "topological" regulation and its derivatives, such as "regulates non-topologically". I don't fully understand what the authors mean: topologically as opposed to "in cis" or as opposed to "in 1D"? Suggest rephrasing.

*Sorry for the confusion, we have removed the “non-topologically” throughout the text. In addition, we changed the second sentence on Page 10 from: “Finally, to confirm this topological regulation of the Pcdh beta and gamma promoters, we performed QHR-4C experiments with each of the super-enhancer CBS repertoire as a viewpoint” to “Finally, to confirm this spatial regulation of the Pcdh beta and gamma promoters, we performed QHR-4C experiments with each of the super-enhancer CBS repertoire as a viewpoint”.*

3. I am personally not a fan of verbless section headings, as it's unclear from them what the message is - only what was perturbed.

*Thanks and we have changed the following section headings:*

*At the beginning of the Results, we have changed the first section title from: “Exogenous CTCF sites as protocadherin insulators.” to “Exogenous directional CTCF sites function as protocadherin insulators in cellular model in vitro”.*

*On top of Page 8, we have changed the section title from: “Augmentation of distal promoter usage by CTCF insulators” to “CTCF insulators enhance distal promoter usage”.*

*Near the top of Page 11, we changed the section title from: “Endogenous CTCF sites as protocadherin insulators.” to: “Endogenous CTCF sites function as protocadherin insulators.”*

*The last section on Page 14, we changed the section title from: “Genome-wide CTCF sites as insulators” to: “Genome-wide CTCF sites function as insulators”.*
